# An Artificial Intelligence Model for Predicting Trauma Mortality Among Emergency Department Patients in South Korea: Retrospective Cohort Study

**DOI:** 10.2196/49283

**Published:** 2023-08-29

**Authors:** Seungseok Lee, Wu Seong Kang, Do Wan Kim, Sang Hyun Seo, Joongsuck Kim, Soon Tak Jeong, Dong Keon Yon, Jinseok Lee

**Affiliations:** 1 Department of Biomedical Engineering Kyung Hee University Yongin Republic of Korea; 2 Department of Trauma Surgery Jeju Regional Trauma Center Cheju Halla General Hospital Jeju Republic of Korea; 3 Department of Thoracic and Cardiovascular Surgery Chonnam National University Hospital Chonnam National University Medical School Gwangju Republic of Korea; 4 Department of Radiology Wonkwang University Hospital Iksan Republic of Korea; 5 Department of Physical Medicine and Rehabilitation Ansanhyo Hospital Ansan Republic of Korea; 6 Department of Pediatrics Kyung Hee University Medical Center Kyung Hee University College of Medicine Seoul Republic of Korea; 7 Center for Digital Health Medical Research Institute Kyung Hee University Medical Center Seoul Republic of Korea

**Keywords:** artificial intelligence, trauma, mortality prediction, international classification of disease, emergency department, ICD, model, models, mortality, predict, prediction, predictive, emergency, death, traumatic, nationwide, national, cohort, retrospective

## Abstract

**Background:**

Within the trauma system, the emergency department (ED) is the hospital’s first contact and is vital for allocating medical resources. However, there is generally limited information about patients that die in the ED.

**Objective:**

The aim of this study was to develop an artificial intelligence (AI) model to predict trauma mortality and analyze pertinent mortality factors for all patients visiting the ED.

**Methods:**

We used the Korean National Emergency Department Information System (NEDIS) data set (N=6,536,306), incorporating over 400 hospitals between 2016 and 2019. We included the International Classification of Disease 10th Revision (ICD-10) codes and chose the following input features to predict ED patient mortality: age, sex, intentionality, injury, emergent symptom, Alert/Verbal/Painful/Unresponsive (AVPU) scale, Korean Triage and Acuity Scale (KTAS), and vital signs. We compared three different feature set performances for AI input: all features (n=921), ICD-10 features (n=878), and features excluding ICD-10 codes (n=43). We devised various machine learning models with an ensemble approach via 5-fold cross-validation and compared the performance of each model with that of traditional prediction models. Lastly, we investigated explainable AI feature effects and deployed our final AI model on a public website, providing access to our mortality prediction results among patients visiting the ED.

**Results:**

Our proposed AI model with the all-feature set achieved the highest area under the receiver operating characteristic curve (AUROC) of 0.9974 (adaptive boosting [AdaBoost], AdaBoost + light gradient boosting machine [LightGBM]: Ensemble), outperforming other state-of-the-art machine learning and traditional prediction models, including extreme gradient boosting (AUROC=0.9972), LightGBM (AUROC=0.9973), ICD-based injury severity scores (AUC=0.9328 for the inclusive model and AUROC=0.9567 for the exclusive model), and KTAS (AUROC=0.9405). In addition, our proposed AI model outperformed a cutting-edge AI model designed for in-hospital mortality prediction (AUROC=0.7675) for all ED visitors. From the AI model, we also discovered that age and unresponsiveness (coma) were the top two mortality predictors among patients visiting the ED, followed by oxygen saturation, multiple rib fractures (ICD-10 code S224), painful response (stupor, semicoma), and lumbar vertebra fracture (ICD-10 code S320).

**Conclusions:**

Our proposed AI model exhibits remarkable accuracy in predicting ED mortality. Including the necessity for external validation, a large nationwide data set would provide a more accurate model and minimize overfitting. We anticipate that our AI-based risk calculator tool will substantially aid health care providers, particularly regarding triage and early diagnosis for trauma patients.

## Introduction

Trauma is the foremost cause of mortality worldwide, especially for those aged under 45 years [1]. Despite recent advances, trauma-related mortality remains a substantial challenge. In the trauma system, the emergency department (ED) is the hospital’s first contact and is vital for allocating medical resources [2]. Decision-making in the ED is crucial and determines further treatment or diagnosis [2,3]; thus, decisions should be made promptly and accurately to reduce the “golden hours” for treatment of a patient with severe trauma. Predicting ED mortality is critical for improving the trauma system and reducing individual, medical staff, and societal burdens. Injury severity, patient demographics, prehospital care, ED trauma care quality, and other complex and multifactorial elements that affect ED mortality pose challenges at gaining an in-depth understanding of the key factors contributing to ED mortality as a whole. Furthermore, ED mortality encompasses considerably more severe injuries than in-hospital mortality, and most patients that die in the ED may not receive additional diagnostic workups or treatments such as computed tomography (CT), angiography, or surgery. Due to hemodynamic instability, a complete workup is nearly impossible for unstable ED trauma patients. Therefore, ED mortality prediction depends on limited information, which could be facilitated by recent advancements in artificial intelligence (AI) technology.

Our research team previously developed two AI models for predicting in-hospital trauma patient mortality [4,5]; one utilizes Abbreviated Injury Scale (AIS) codes and the other incorporates the International Classification of Diseases, 10th revision (ICD-10) codes and other variables from the Korean National Emergency Department Information System (NEDIS) data set. Deceased ED patients were excluded from these models, as we assumed they may have received an insufficient workup or indicate an inaccurate diagnosis in previous studies. These AI models exhibited high accuracy for predicting in-hospital mortality but did not effectively learn from deceased ED patient data.

We deemed that ED patients who died before admission sustained severe injuries and that their information was insufficient compared to that available for patients who died after intensive care unit (ICU) or ward admission. We concluded that ED mortality should be predicted using a different patient data set than in-hospital mortality, and that the new model should also have alternative weights and input variables. Therefore, in this study, we developed AI models for predicting ED mortality in trauma patients using the NEDIS data set that was not incorporated into the previous AI models [4,5].

## Methods

### Patients and Data Set

The Korean National Emergency Medical Center has gathered NEDIS data from over 400 hospitals in Korea since 2016. This study employed the 2016 to 2019 NEDIS data set (data acquisition number N20212920825) to develop an AI model for predicting trauma mortality among all patients visiting the ED according to the Transparent Reporting of a Multivariable Model for Individual Prognosis or Diagnosis (TRIPOD) statement [6]. Data used for the AI model included those related to patients experiencing physical trauma (but not psychological) with an “S” or “T” diagnostic code from the ICD-10 (N=7,664,443); the S code represents trauma in a single body region, whereas T signifies trauma in multiple or unspecified regions. All ED patient data with an S or T diagnosis code were included, regardless of ward or ICU admittance or discharge.

**Figure 1:**
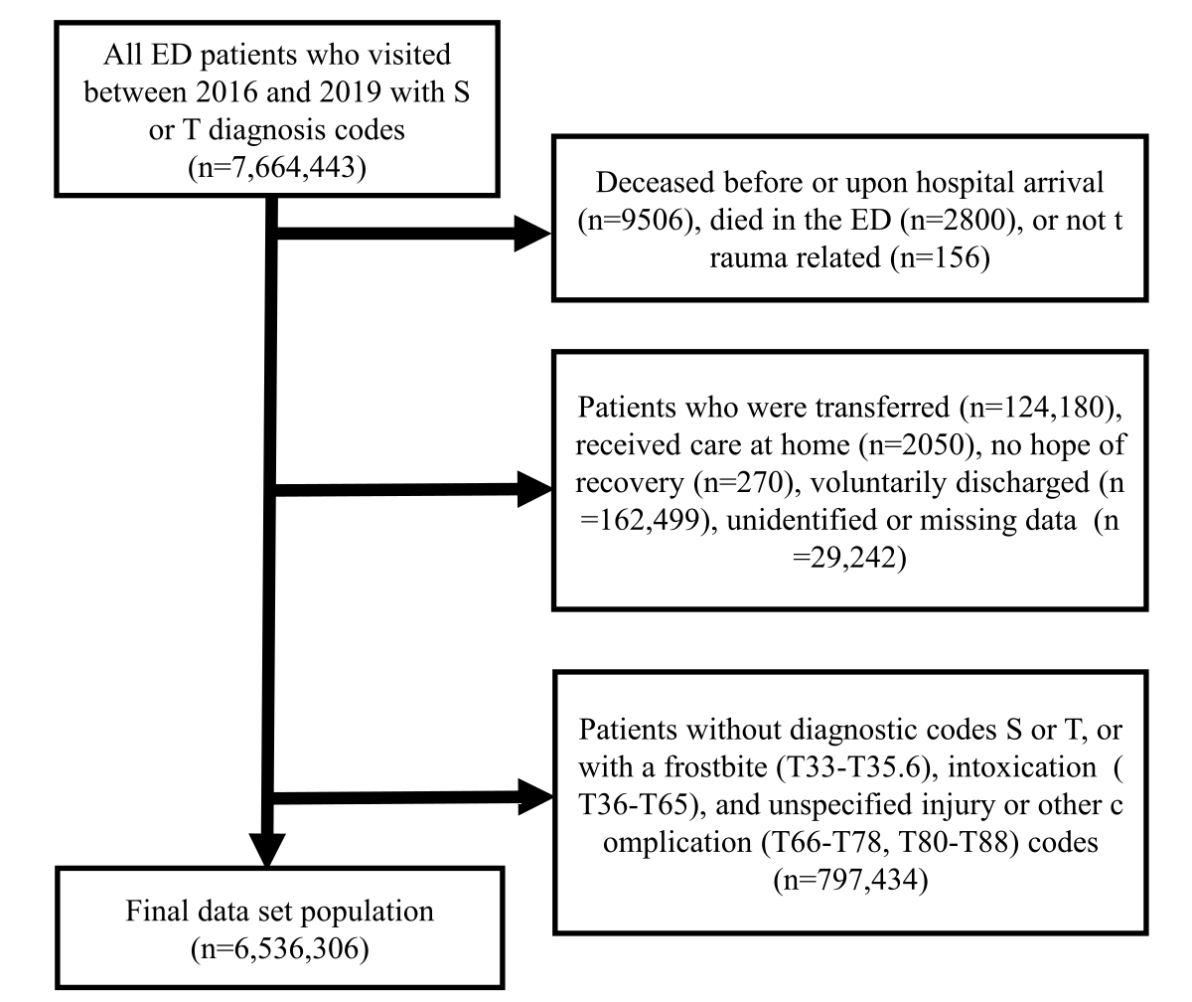
Patient selection process. ED: emergency department; S: International Classification of Diseases (ICD) code to signify trauma in a single body region; T: ICD code to signify trauma in multiple or unspecified regions.

Patient data with the following conditions were excluded: (1) patients who died before or upon hospital arrival (n=9506, 0.12%), who were regarded as dead-on-arrival and did not undergo any treatment or cardiopulmonary resuscitation; (2) ED patients who died due to severe conditions without further treatment, such as cardiac arrest without receiving cardiopulmonary resuscitation, despite being brought to the hospital alive (n=2800, 0.04%); (3) nontrauma patients (n=156, 0.002%); (4) patients transferred to another hospital from the ED (n=124,180, 1.62%); (5) patients with end-stage disease such as cancer who wished to receive care at home (n=2050, 0.03%); (6) patients who had to leave the hospital because there was no hope of recovery (n=270, 0.003%); (7) voluntarily discharged patients (n=162,499, 2.12%); (8) patients for whom it was difficult to record data due to being unidentifiably detained for criminal activity (n=29,242, 0.38%); (9) patients without S or T diagnostic codes, since all physical trauma receives an S or T diagnostic code; and (10) patients with frostbite (ICD-10 T33-T35.6), intoxication (ICD-10 T36-T65), or unspecified injury or complication diagnostic codes (ICD-10 T66-T78, T80-T88) (n=797,434, 10.4%). Our AI model was trained and tested using data from 6,536,306 patients. Figure 1 presents the patient selection process.

### AI Model Variables

This study followed Developing and Reporting Machine Learning Predictive Models in Biomedical Research guidelines [7]. We used the following 14 NEDIS data variables for AI model input: age; gender; intentionality; injury mechanism; emergent symptom; Alert, Verbal, Painful, Unresponsive (AVPU) scale; initial Korean Triage and Acuity Scale (KTAS); systolic blood pressure; diastolic blood pressure; pulse rate per minute; respiratory rate per minute; body temperature; oxygen saturation; and ICD-10 codes. Moreover, intentionality includes six categories: accidental/unintentional, self-harm/suicide, violence/assault, other specified, unspecified, and missing data. The injury mechanism comprises 16 categories: car accident, bike accident, motorcycle accident, other traffic accidents, unspecified traffic accident, fall, slipped, struck, firearm/cut/pierce, machine, fire/flames/heat, drowning, poisoning, choking/hanging, others, and unknown. Emergency and nonemergency are the two emergent symptom categories. The AVPU scale is a simplified version of the Glasgow Coma Scale (GCS) [8,9] and includes four categories: A, alert; V, verbal responsive (drowsy); P, painful response (stupor, semicoma); and U, unresponsive (coma).

The KTAS is a standardized triage tool that avoids complexity and ambiguity by employing five categories: Level 1, resuscitation; Level 2, emergent; Level 3, urgent; Level 4, less urgent; and Level 5, nonurgent. The KTAS was initially developed as an ED severity triage in 2012, based on the Canadian Triage and Acuity Scale [10]. According to NEDIS policy, a certified faculty member must conduct the initial KTAS within 2 minutes of ED admission. ICD-10 codes starting with S or T entail 865 categories. All AI model variables are summarized in Table S1 of Multimedia Appendix 1. The mortality of patients visiting the ED was defined as a patient with a dead result code or an indication of discharge with medical futility in NEDIS.

### Data Split and Cross-Validation

Training and testing data used in this study are detailed in Table S2 of Multimedia Appendix 1. Data from 6,536,306 patients were divided into training (n=5,229,008) and testing (n=1,307,252) data sets with an 8:2 ratio in a stratified fashion. Only the testing set was used to test our developed AI model. We first performed 5-fold cross-validation in the training data set to assess how the prediction results generalize to an independent data set. The training data set was randomly shuffled and stratified into five equal groups, of which four were selected for training and the remaining group was used for internal validation. This process was repeated five times by shifting the internal validation group.

### Handling Data Imbalance

The data were severely imbalanced as there were 6351 (0.1%) deceased patients recorded (Table S2 in Multimedia Appendix 1). To minimize the model bias toward a majority (ie, the survived patient group), we used the synthetic minority oversampling technique (SMOTE) [11] to upsample the deceased patient quantity and match the survived patient group. Next, we identified each group’s optimized weight values for the loss function during model training. We then iteratively learned demographic parity-based coefficients for the weight value search [12], providing a closed-form expression for the data weights. These two methods prevented bias toward survived patient data.

### Machine Learning Models

We used three feature sets to develop our AI model: the first set included all 921 NEDIS variables and 878 features from ICD-10 codes, the second set only used the 878 features from ICD-10 codes, and the third set utilized all features except those from ICD-10 codes. We applied eight machine learning models from each feature set: adaptive boosting (AdaBoost) [13], extreme gradient boosting (XGBoost) [14], light gradient boosting machine (LightGBM) [15], gradient boosting machine (GBM) [16], extremely random trees (ERT) [17], logistic regression (LR) [18], random forest (RF) [19], and deep neural network (DNN). We chose the best three among the eight models and applied an ensemble approach by considering all possible combinations. Finally, we evaluated the feature importance, listing features in the order they contributed to mortality prediction.

In decision tree approaches such as AdaBoost, XGBoost, and LightGBM, calculating feature importance values hinges on assessing the decrease in node impurity while factoring in the probability of reaching each node. Node impurity is determined using a well-established metric called the Gini index, which quantifies the impurity degree at a given node by measuring the extent to which a specific variable would be incorrectly classified if selected randomly. The impurity is evaluated by considering the weighted sum of each class’s squared probabilities within the node. This comprehensive methodology incorporates node probabilities, impurity measures, and feature importance calculations, allowing for a nuanced understanding of how different variables impact prediction outcomes and can offer valuable insights for decision-making and analysis.

Performance evaluations were based on 5-fold cross-validation using the following metrics: sensitivity, specificity, accuracy, balanced accuracy, and area under the receiver operating characteristic (ROC) curve (AUROC). Due to the significant data imbalance, we used balanced accuracy as the primary model evaluation metric.

The models used Python (version 3.7.13), NumPy (version 1.21.6), Pandas (version 1.3.5), Matplotlib (version 3.5.1), and Scikit-learn (version 1.0.2). All statistical analyses were performed with R software version 4.1.2 (R Foundation for Statistical Computing). Continuous variables are presented using the mean and standard deviation, while categorical data are presented using proportions. Statistical continuous data comparisons were performed using the Student *t*-test or the Mann-Whitney *U* test as appropriate. Similarly, proportions were compared through *χ*^2^ or Fisher exact tests as appropriate. A two-sided *P* value <.05 was considered statistically significant.

### Conventional Metrics

To further evaluate the performance of our AI model, we implemented ICD-10–based conventional metrics for comparison: inclusive survival risk ratio (SRR), exclusive SRR, and KTAS. The ICD-based Injury Severity Score (ICISS) utilizes the SRR to calculate survival probability [20]. SRRs can be quantified as the number of survived patients with a specific injury code divided by all patients with the same code. Patient survival probability was determined by multiplying all patient injury code SRRs [20]. The traditional ICISS was calculated as the survival probability product for up to 10 injuries [21]. There are two different SRR calculation approaches: inclusive and exclusive SRR. Inclusive SRR can be calculated for each injury without considering the associated injury. In contrast, exclusive SRR divides the number of survivors with an isolated specific injury by the total number of patients with only that injury. Thus, patients with multiple injuries were excluded from exclusive SRR calculations [20]. We used the survival probability determined from our previous study [5] because other studies [20,21] did not use ED mortality.

### Ethical Approval

The Institutional Review Board of Wonkwang University Hospital approved this study (WKUH 2019-11-004-001). The requirement for informed consent was waived due to the study’s observational nature and the deidentification of each patient.

## Results

### Patients

Table 1 shows a comparison of the main variables between the deceased and survived patients among all patients visiting the ED, whereas Table S3 of Multimedia Appendix 1 compares ICD-10 codes between deceased and survived patients (878 features).

**Table 1:** National Emergency Department Information System variable comparison between deceased and survived patients among all patients visiting the emergency department.

Variables			Deceased (n=6351)		Survived (n=6,529,909)		*P* value	
**Age (years), n (%)**								<.001
	<1	7 (0.1)		91,585 (1.4)				
	1-4	36 (0.6)		711,018 (10.9)				
	5-9	41 (0.6)		425,503 (6.5)				
	10-14	53 (0.8)		289,072 (4.4)				
	15-19	190 (3.0)		329,548 (5.0)				
	20-24	271 (4.3)		412,019 (6.3)				
	25-29	272 (4.3)		433,147 (6.6)				
	30-34	275 (4.3)		386,563 (5.9)				
	35-39	277 (4.3)		414,559 (6.3)				
	40-44	323 (5.1)		386,061 (5.9)				
	45-49	488 (7.7)		442,058 (6.8)				
	50-54	573 (9.0)		456,820 (7.0)				
	55-59	692 (10.9)		480,396 (7.4)				
	60-64	576 (9.1)		362,980 (5.6)				
	65-69	505 (8.0)		244,581 (3.7)				
	70-74	488 (7.7)		199,392 (3.1)				
	75-79	587 (9.2)		196,499 (3.0)				
	80-84	420 (6.6)		149,770 (2.3)				
	85-89	196 (3.1)		81,181 (1.2)				
	90-94	52 (0.8)		29,114 (0.4)				
	95-99	16 (0.3)		7025 (0.1)				
	100-104	12 (0.2)		840 (0)				
	105-109	0 (0)		111 (0)				
	110-114	0 (0)		42 (0)				
	115-119	1 (0)		24 (0)				
	≥120	0 (0)		1 (0)				
**Initial KTAS^a^, n (%)**								
	Level 1	5214 (82.1)		10,941 (0.2)		<.001		
	Level 2	795 (12.5)		134,003 (2.1)		<.001		
	Level 3	219 (3.4)		1,044,687 (16.0)		<.001		
	Level 4	49 (0.8)		4,211,165 (64.5)		<.001		
	Level 5	1 (0)		858,996 (13.2)		<.001		
	Not classified	0 (0)		227 (0)		.99		
	Missing data	73 (1.1)		269,890 (4.1)		<.001		
**Intentionality, n (%)**								
	Accidental, unintentional	4734 (74.5)		5,151,631 (78.9)		<.001		
	Suicide, intentional self-harm	399 (6.3)		28,347 (0.4)		<.001		
	Assault, violence	74 (1.2)		191,857 (2.9)		<.001		
	Other specified	217 (3.4)		21,874 (0.3)		<.001		
	Unspecified	707 (11.1)		82,701 (1.3)		<.001		
	Missing data	220 (3.5)		1,053,499 (16.1)		<.001		
**Injury mechanism, n (%)**								
	Car accident	1073 (16.9)		575,270 (8.8)		<.001		
	Bike accident	155 (2.4)		130,385 (2.0)		.01		
	Motorcycle accident	579 (9.1)		128,419 (2.0)		<.001		
	Traffic accident-pedestrian, train, airplane, ship, etc	1521 (23.9)		173,313 (2.7)		<.001		
	Traffic accident-unknown	32 (0.5)		769 (0.0)		<.001		
	Fall	1790 (28.2)		379,735 (5.8)		<.001		
	Slipped	80 (1.3)		1,087,804 (16.7)		<.001		
	Struck by person or object	233 (3.7)		1,173,585 (18.0)		<.001		
	Firearm, cut, or pierced	149 (2.3)		787,042 (12.1)		<.001		
	Machine	46 (0.7)		62,910 (1.0)		<.001		
	Fire, flames, or heat	29 (0.5)		175,797 (2.7)		<.001		
	Drowning or nearly drowning	16 (0.3)		546 (0.0)		<.001		
	Poisoning	6 (0.1)		7,639 (0.1)		.73		
	Choking, hanging	105 (1.7)		3,817 (0.1)		<.001		
	Others-rape, electric	62 (1.0)		684,468 (10.5)		<.001		
	Unknown	255 (4.0)		104,911 (1.6)		<.001		
	Missing data	220 (3.5)		1,053,499 (16.1)		<.001		
**Emergent symptom, n (%)**								
	Yes	6262 (98.6)		5,245,303 (80.3)		<.001		
	No	89 (1.4)		1,284,606 (19.7)		<.001		
	Unspecified	0 (0.0)		0 (0.0)				
**AVPU^b^ scale, n (%)**								
	Alert	364 (5.7)		5,340,780 (81.8)		<.001		
	Verbal response (drowsy)	238 (3.7)		41,488 (0.6)		<.001		
	Painful response (stupor, semicoma)	532 (8.3)		20,004 (0.3)		<.001		
	Unresponsive (coma)	4973 (78.3)		5,695 (0.1)		<.001		
	Unspecified response	244 (3.8)		1,121,942 (17.2)		<.001		
Male sex, n (%)			4446 (70.0)		3,839,715 (58.8)		<.001	
Systolic blood pressure, mean (SD)			125.98 (22.76)		132.39 (18.24)		<.001	
Diastolic blood pressure, mean (SD)			76.71 (13.57)		79.83 (11.29)		<.001	
Pulse rate per minute, mean (SD)			89.71 (15.50)		87.59 (15.92)		<.001	
Respiratory rate per minute, mean (SD)			20.07 (3.00)		19.94 (2.84)		.001	
Body temperature, mean (SD)			36.15 (0.93)		36.62 (0.40)		<.001	
Oxygen saturation, mean (SD)			94.86 (9.72)		98.08 (1.56)		<.001	

^a^KTAS: Korean Triage and Acuity Scale.

^c^AVPU: Alert/Verbal/Painful/Unresponsive.

### K-Fold Cross-Validation

Table 2 summarizes the 5-fold cross-validation results. The AdaBoost model with all 921 features achieved the highest balanced accuracy (0.9801) and AUROC (0.9973) values among the seven models: XGBoost, LightGBM, AdaBoost with XGBoost, AdaBoost with Light GBM, XGBoost with LightGBM, and the three models combined. In addition, the 921-feature model provided higher accuracy metrics than the 878-feature model with the ICD-10 codes and the 43-feature model excluding the ICD-10 codes. Interestingly, the model with all features except for the ICD-10 codes reached higher accuracy metrics than the 878-feature model with the ICD-10 codes.

These results substantiate that patient information, symptoms, and trauma causes predict mortality better than ICD-10 codes. We also compared the performance of traditional methods. Inclusive SRR, exclusive SRR, and KTAS exhibited lower balanced accuracies (0.9069, 0.9175, and 0.9619, respectively) and AUROCs (0.9345, 0.9554, and 0.9372, respectively).

**Table 2:** Five-fold cross-validation result comparison.

Model			Sensitivity, mean (SD)		Specificity, mean (SD)		Accuracy, mean (SD)		Balanced accuracy, mean (SD)		AUROC^a^, mean (SD)
**921 features (including ICD-10^b^)**											
	AdaBoost^c^	0.9713 (0.0060)		0.9890 (0.0040)		0.9801 (0.0035)		0.9801 (0.0035)		0.9973 (0.0005)	
	XGBoost^d^	0.9674 (0.0035)		0.9897 (0.0034)		0.9786 (0.0016)		0.9786 (0.0016)		0.9968 (0.0004)	
	LightGBM^e^	0.9678 (0.0034)		0.9898 (0.0034)		0.9788 (0.0015)		0.9788 (0.0015)		0.9968 (0.0005)	
	GBM^f^	0.7952 (0.0088)		0.9475 (0.0074)		0.8713 (0.0059)		0.8713 (0.0059)		0.9319 (0.0054)	
	ERT^g^	0.8944 (0.0106)		0.9248 (0.0110)		0.9095 (0.0063)		0.9095 (0.0063)		0.9542 (0.0051)	
	LR^h^	0.9514 (0.0052)		0.9933 (0.0030)		0.9723 (0.0026)		0.9723 (0.0026)		0.9717 (0.0027)	
	RF^i^	0.9310 (0.0116)		0.9667 (0.0086)		0.9488 (0.0206)		0.9488 (0.0116)		0.9872 (0.0034)	
	DNN^j^	0.9708 (0.0058)		0.9847 (0.0048)		0.9778 (0.0038)		0.9778 (0.0038)		0.9944 (0.0012)	
	AdaBoost+XGBoost	0.9675 (0.0036)		0.9899 (0.0034)		0.9787 (0.0016)		0.9787 (0.0016)		0.9970 (0.0005)	
	AdaBoost+LightGBM	0.9681 (0.0034)		0.9900 (0.0033)		0.9790 (0.0014)		0.9790 (0.0014)		0.9970 (0.0005)	
	XGBoost+LigtGBM	0.9675 (0.0036)		0.9899 (0.0034)		0.9787 (0.0016)		0.9787 (0.0016)		0.9968 (0.0004)	
	AdaBoost+XGBoost+LightGBM	0.9675 (0.0036)		0.9899 (0.0034)		0.9787 (0.0016)		0.9787 (0.0016)		0.9970 (0.0005)	
**878 features (ICD-10 only)**											
	AdaBoost	0.8261 (0.0073)		0.9429 (0.0070)		0.8845 (0.0053)		0.8845 (0.0053)		0.9448 (0.0056)	
	XGBoost	0.6801 (0.0172)		0.9722 (0.0065)		0.8261 (0.0095)		0.8261 (0.0095)		0.8929 (0.0051)	
	LightGBM	0.6877 (0.0140)		0.9717 (0.0071)		0.8297 (0.0072)		0.8297 (0.0072)		0.8939 (0.0056)	
	GBM	0.7952 (0.0088)		0.9475 (0.0074)		0.8713 (0.0059)		0.8713 (0.0059)		0.9319 (0.0054)	
	ERT	0.8944 (0.0106)		0.9248 (0.0110)		0.9096 (0.0063)		0.9096 (0.0063)		0.9542 (0.0051)	
	LR	0.7535 (0.0110)		0.9540 (0.0060)		0.8537 (0.0055)		0.8537 (0.0054)		0.9401 (0.0066)	
	RF	0.6615 (0.0424)		0.9724 (0.0125)		0.8169 (0.0185)		0.8169 (0.0185)		0.9265 (0.0070)	
	DNN	0.9329 (0.0158)		0.9788 (0.0126)		0.9559 (0.0059)		0.9559 (0.0059)		0.9867 (0.0023)	
	AdaBoost+XGBoost	0.6931 (0.0101)		0.9719 (0.0068)		0.8325 (0.0060)		0.8325 (0.0059)		0.9408 (0.0047)	
	AdaBoost+LightGBM	0.6960 (0.0124)		0.9715 (0.0070)		0.8337 (0.0068)		0.8337 (0.0068)		0.9408 (0.0048)	
	XGBoost+LigtGBM	0.6824 (0.0150)		0.9719 (0.0068)		0.8271 (0.0089)		0.8271 (0.0089)		0.8939 (0.0055)	
	AdaBoost+XGBoost+LightGBM	0.6908 (0.0104)		0.9718 (0.0070)		0.8312 (0.0063)		0.8313 (0.0063)		0.9405 (0.0048)	
**43 features (excluding ICD-10)**											
	AdaBoost	0.9707 (0.0050)		0.9854 (0.0062)		0.9781 (0.0020)		0.9781 (0.0020)		0.9965 (0.0007)	
	XGBoost	0.9658 (0.0040)		0.9889 (0.0039)		0.9773 (0.0014)		0.9773 (0.0014)		0.9960 (0.0005)	
	LightGBM	0.9661 (0.0040)		0.9887 (0.0041)		0.9774 (0.0013)		0.9774 (0.0013)		0.9961 (0.0004)	
	GBM	0.9729 (0.0036)		0.9858 (0.0054)		0.9793 (0.0021)		0.9793 (0.0021)		0.9965 (0.0006)	
	ERT	0.9712 (0.0041)		0.9828 (0.0052)		0.9770 (0.0024)		0.9770 (0.0024)		0.9937 (0.0011)	
	LR	0.9448 (0.0053)		0.9921 (0.0029)		0.9685 (0.0023)		0.9685 (0.0023)		0.9941 (0.0009)	
	RF	0.9079 (0.0089)		0.9503 (0.0107)		0.9291 (0.0061)		0.9291 (0.0062)		0.9818 (0.0018)	
	DNN	0.8805 (0.0482)		0.8903 (0.0465)		0.8854 (0.0104)		0.8854 (0.0104)		0.9424 (0.0050)	
	AdaBoost+XGBoost	0.9660 (0.0039)		0.9888 (0.0040)		0.9774 (0.0013)		0.9774 (0.0013)		0.9962 (0.0005)	
	AdaBoost+LightGBM	0.9661 (0.0039)		0.9890 (0.0041)		0.9775 (0.0012)		0.9775 (0.0012)		0.9962 (0.0005)	
	XGBoost+LigtGBM	0.9659 (0.0039)		0.9889 (0.0040)		0.9774 (0.0012)		0.9774 (0.0012)		0.9960 (0.0005)	
	AdaBoost+XGBoost+LightGBM	0.9661 (0.0039)		0.9891 (0.0041)		0.9776 (0.0013)		0.9776 (0.0013)		0.9961 (0.0005)	
**Traditional methods**											
	Inclusive SRR^k^	0.9271		0.8867		0.8867		0.9069		0.9345	
	Exclusive SRR	0.9250		0.9100		0.9100		0.9175		0.9554	
	KTAS^l^	0.9461		0.9778		0.9778		0.9619		0.9372	

^a^AUROC: area under the receiver operating characteristic curve.

^b^ICD-10: International Classification of Disease 10th Revision.

^c^AdaBoost: adaptive boosting.

^d^XGBoost: extreme gradient boosting.

^e^LightGBM: light gradient boosting machine.

^f^GBM: gradient boosting machine.

^g^ERT: extremely random trees.

^h^LR: logistic regression.

^i^RF: random forest.

^j^DNN: deep neural network.

^k^SRR: survival risk ratio.

^l^KTAS: Korean Triage and Acuity Scale.

### Ranked Feature Importance: Explainable AI

**Figure 2:**
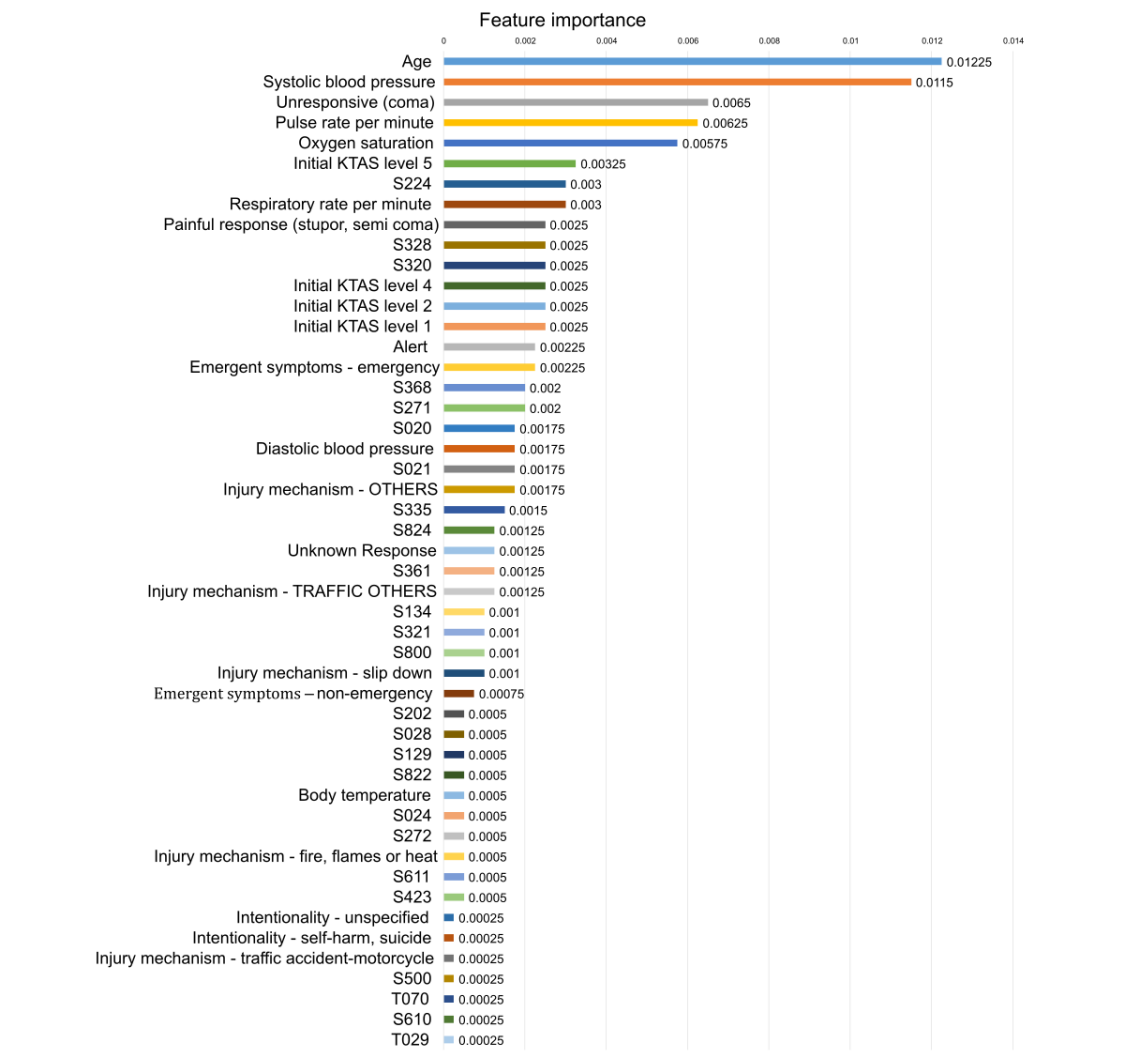
Ranked normalized feature importance from the selected AdaBoost model. KTAS: Korean Triage and Acuity Scale. See Table S5 in Multimedia Appendix 1 for the definition of "S" and "T" International Classification of Diseases 10th revision codes.

Next, we conducted a feature importance analysis to confirm each feature’s contribution. Figure 2 ranks the normalized feature importance from the AdaBoost model, which showed the best performance. Age and systolic blood pressure were the top two mortality predictors among visiting ED patients, followed by unresponsiveness (coma), pulse rate per minute, oxygen saturation, KTAS Level 5, S224 (multiple rib fractures), respiratory rate per minute and painful response (stupor and semicoma). Interestingly, only 49 among the 921 features had nonzero importance values, indicating that the other 872 features did not contribute to mortality prediction. Table S4 in Multimedia Appendix 1 summarizes the total ranked normalized feature importance values.

### Testing Data

**Figure 3:**
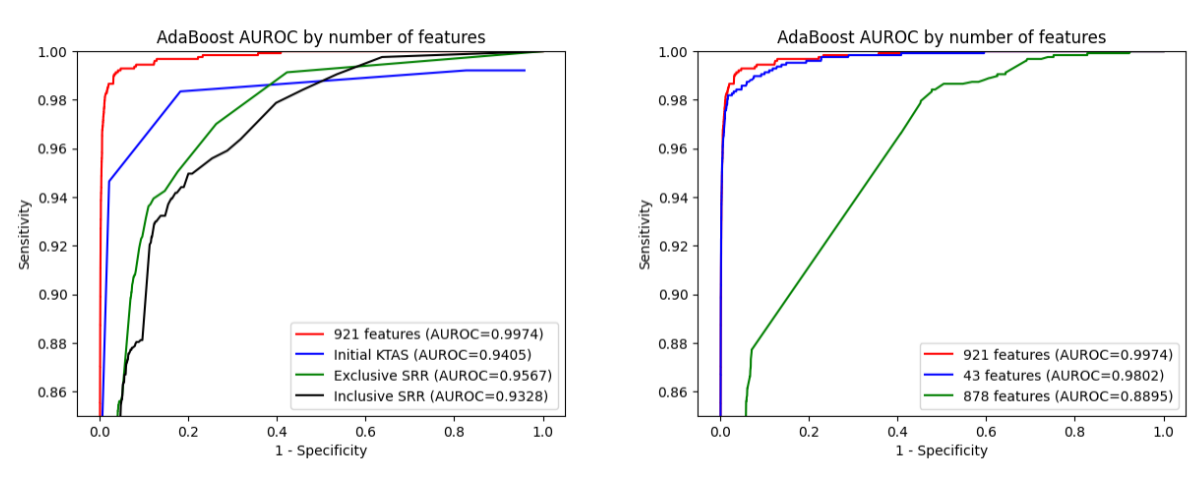
Receiver operating characteristic curves for the (left) selected adaptive boosting (AdaBoost) and three traditional models and (right) relative AdaBoost features. AUROC: area under the receiver operating characteristic curve; ICD-10: International Classification of Diseases 10th revision; KTAS: Korean Triage and Acuity Scale; SRR: survival risk ratio.

Table 3 summarizes the results from the isolated testing data set results (n=1,307,252). The testing data results corroborate that the 921-feature AdaBoost model achieved the highest balanced accuracy (0.9813) and AUROC (0.9974) values among the seven models: XGBoost, LightGBM, AdaBoost with XGBoost, AdaBoost with Light GBM, XGBoost with LightGBM, and the three-model combination. Similar to the cross-validation results, the 921-feature model provided higher accuracy metrics than the 878-feature model with ICD-10 codes and the 43-feature model excluding ICD-10 codes. Furthermore, our selected model also performed better than traditional inclusive SRR, exclusive SRR, and KTAS methods (see Table S5 in Multimedia Appendix 1). The similarity between the cross-validation and testing data results denotes minimal overfitting or underfitting. Figure 3 depicts the ROC curve comparison, including comparison of the selected AdaBoost model and the three traditional models (left) and the selected AdaBoost model relative to the 921-, 878-, and 43-feature models (right).

Finally, we compared the performance of the cutting-edge AI model, as it was designed for in-hospital mortality predictions based on the NEDIS data set [5]. The in-hospital mortality model’s balanced accuracy and AUROC values were lower, regardless of the feature set. Notably, the in-hospital mortality model with all 921 features obtained 0.9614 balanced accuracy and 0.9929 AUROC values, and the model with 43 features excluding ICD-10 yielded a 0.9648 balanced accuracy and 0.9923 AUROC. Interestingly, when using the in-hospital mortality model with only 878 features and the ICD-10 codes, the values significantly dropped to a 0.6298 balanced accuracy and 0.7675 AUROC.

**Table 3:** Model prediction comparisons with the test data set.

Model		Sensitivity	Specificity	Accuracy	Balanced accuracy	AUROC^a^
**921 features (including ICD-10^b^)**						
	AdaBoost^c^	0.9739	0.9888	0.9887	0.9813	0.9974
	XGBoost^d^	0.9689	0.9888	0.9888	0.9789	0.9972
	LightGBM^e^	0.9701	0.9887	0.9887	0.9794	0.9973
	GBM^f^	0.7954	0.9506	0.9504	0.8730	0.9350
	ERT^g^	0.9010	0.9270	0.9270	0.9140	0.9580
	LR^h^	0.9584	0.9922	0.9922	0.9753	0.9952
	RF^i^	0.9433	0.9537	0.9538	0.9476	0.9848
	DNN^j^	0.9694	0.9845	0.9845	0.9770	0.9931
	AdaBoost+XGBoost	0.9700	0.9889	0.9889	0.9794	0.9973
	AdaBoost+LightGBM	0.9702	0.9888	0.9888	0.9795	0.9974
	XGBoost+LigtGBM	0.9694	0.9888	0.9888	0.9791	0.9973
	AdaBoost+XGBoost+LightGBM	0.9698	0.9888	0.9888	0.9793	0.9973
	In-hospital mortality AI^k^ [5]	0.9468	0.9761	0.9760	0.9614	0.9929
**878 features (ICD-10 only)**						
	AdaBoost	0.8313	0.9476	0.9475	0.8895	0.9485
	XGBoost	0.6933	0.9751	0.9748	0.8342	0.8943
	LightGBM	0.6995	0.9745	0.9742	0.8370	0.8953
	GBM	0.9730	0.9774	0.9774	0.9752	0.9958
	ERT	0.9739	0.9810	0.9810	0.9775	0.9938
	LR	0.7623	0.9558	0.9557	0.8591	0.9437
	RF	0.6583	0.9740	0.9737	0.8161	0.9313
	DNN	0.8809	0.8959	0.8959	0.8884	0.9438
	AdaBoost+XGBoost	0.7046	0.9747	0.9744	0.8397	0.9452
	AdaBoost+LightGBM	0.7057	0.9744	0.9741	0.8400	0.9452
	XGBoost+LigtGBM	0.6950	0.9748	0.9745	0.8349	0.8954
	AdaBoost+XGBoost+LightGBM	0.7024	0.9746	0.9743	0.8385	0.9450
	In-hospital mortality AI [5]	0.2838	0.9751	0.9751	0.6298	0.7675
**43 features (excluding ICD-10)**						
	AdaBoost	0.9743	0.9862	0.9862	0.9802	0.9965
	XGBoost	0.9684	0.9886	0.9886	0.9785	0.9965
	LightGBM	0.9691	0.9884	0.9884	0.9788	0.9966
	GBM	0.7954	0.9506	0.9504	0.8730	0.9350
	ERT	0.9010	0.9270	0.9270	0.9140	0.9580
	LR	0.9519	0.9906	0.9906	0.9713	0.9935
	RF	0.9040	0.9492	0.9492	0.9266	0.9806
	DNN	0.9345	0.9784	0.9783	0.9565	0.9860
	AdaBoost+XGBoost	0.9687	0.9887	0.9887	0.9787	0.9966
	AdaBoost+LightGBM	0.9693	0.9886	0.9885	0.9789	0.9966
	XGBoost+LigtGBM	0.9690	0.9885	0.9885	0.9788	0.9965
	AdaBoost+XGBoost+LightGBM	0.96914	0.9886	0.9885	0.9788	0.9966
	In-hospital mortality AI [5]	0.9441	0.9855	0.9855	0.9648	0.9923
**Traditional methods**						
	Inclusive SRR^l^	0.8716	0.9389	0.9388	0.9052	0.9328
	Exclusive SRR	0.9070	0.9227	0.9227	0.9149	0.9567
	KTAS^m^	0.9465	0.9777	0.9777	0.9621	0.9405

^a^AUROC: area under the receiver operating characteristic curve.

^b^ICD-10: International Classification of Disease 10th revision.

^c^AdaBoost: adaptive boosting.

^d^XGBoost: extreme gradient boosting.

^e^LightGBM: light gradient boosting machine.

^f^GBM: gradient boosting machine.

^g^ERT: extremely random trees.

^h^LR: logistic regression.

^i^RF: random forest.

^j^DNN: deep neural network.

^k^AI: artificial intelligence.

^l^SRR: survival risk ratio.

^m^KTAS: Korean Triage and Acuity Scale.

### AI-Driven Public Website Deployment

We launched our final AI model on a public website [22] to allow access to the mortality prediction results among visiting ED patients. Figure S1A in Multimedia Appendix 1 displays the web interface for entering information. A user inputs age, gender, intentionality, injury mechanism, emergent symptoms, AVPU scale, initial KTAS, systolic blood pressure, diastolic blood pressure, pulse rate per minute, respiratory rate per minute, body temperature, oxygen saturation, and ICD-10 codes. For ICD-10 codes, a user can input multiple codes with commas (eg, S072, S224, T083). After entering information into the web app, the user can obtain the mortality prediction results (see Figure S1B in Multimedia Appendix 1 for an example). The prediction results also include the mortality probability.

### Towards Comprehensive Model

**Figure 4:**
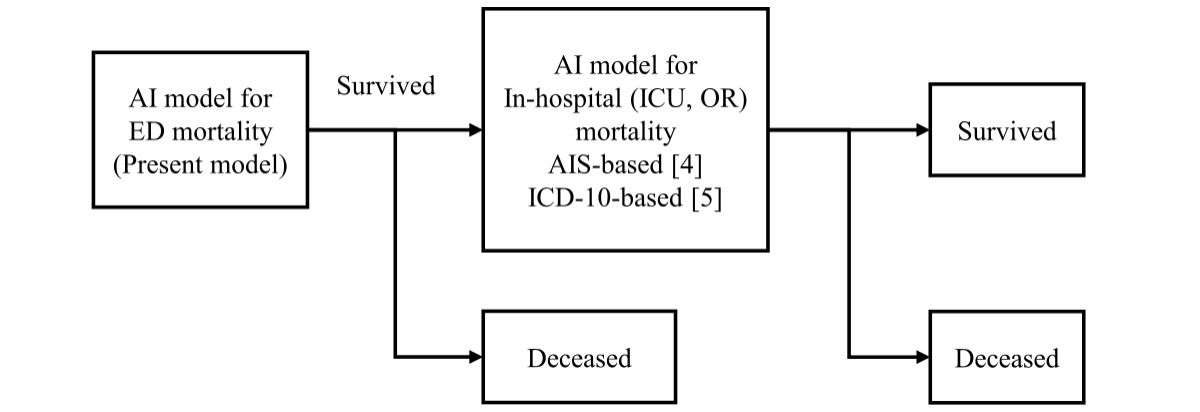
A new pipeline for predicting emergency department (ED) and in-hospital mortality [4,5]. AI: artificial intelligence; AIS: Abbreviated Injury Scale; ICD-10: International Classification for Diseases 10th revision; ICU: intensive care unit; OR: operating room.

Our previous study [5] revealed that blood pressure, heart rate, body temperature, and other vital signs weakened the previous AI model’s performance, whereas incorporating vital signs strengthened our present AI model. This observation implies that ED and in-hospital mortality patients exhibit differing data distributions. Patients who died after ICU or ward admission and not in the ED were labeled survivors in the present AI model; therefore, the present AI model predicts a more severe mortality type. In future studies, we plan to design a more comprehensive model incorporating both in-hospital and ED mortality. We propose a new pipeline for predicting ED and in-hospital mortality based on our present AI model and the previous two models [4,5] (Figure 4).

## Discussion

### Principal Findings

This study developed an AI model that accurately predicts mortality among visiting ED patients. Our final AI model achieved substantially high metrics of 0.9738 sensitivity, 0.9888 specificity, 0.9887 accuracy, 0.9813 balanced accuracy, and 0.9974 AUROC. In addition, the proposed AI model outperformed traditional inclusive SRR, exclusive SRR, and KTAS models and the previously developed in-hospital mortality prediction AI model [5]. We identified several significant mortality predictors through the feature importance analysis, including age, AVPU scale, multiple rib fractures, lumbar vertebra fractures, and KTAS Level 2. Furthermore, we devised a risk calculator leveraging our AI model, demonstrating its substantial clinical potential for triage and diagnosis.

We compared AdaBoost, XGBoost, LightGBM, GBM, ERT, LR, RF, and DNN models. After determining that AdaBoost, XGBoost, and LightGBM models were the top three, these models were combined as an ensemble approach. During the evaluation, we considered balanced accuracy as the primary metric due to the data imbalance. Using the best three single models included the boosting algorithm principle, another ensemble approach. Boosting incorporates the stagewise addition method, where multiple weak models are trained and combined into one stronger model. Specifically, the AdaBoost grows decision trees as weak models and adds penalties or weights to the incorrectly predicted samples. After each prediction stage, this process assigns higher weight values to the misclassified samples.

Although XGBoost and LightGBM also utilize boosting algorithms, they differ slightly from AdaBoost regarding the gradient boosting algorithm. The gradient boosting algorithm adjusts the new prediction model using the previous model’s residual errors. Thus, XGBoost computes the residuals and builds decision trees by selecting features, finding optimal splits, estimating leaf node values, and applying regularization. The predictions are updated and the residuals are recalculated at each iteration. LightGBM resembles XGBoost in many aspects, but it has a faster execution rate and maintains high accuracy levels with gradient-based one-side sampling (GOSS) and exclusive feature bundling. In contrast, XGBoost uses a presorted and histogram-based algorithm for computing the best split with GOSS in LightGBM.

We also assessed GBM during the initial single-model evaluation. Both LightGBM and GBM models are founded on gradient boosting frameworks but differ in tree construction, feature discretization, gradient computation, and memory usage. Notably, each machine learning model performed better under different data characteristics. For instance, LR may work well with a linear association between features and a target, whereas a decision tree is more effective for nonlinear relationships. Other models with similar GBM and LightGBM operations can also produce different prediction outcomes due to slightly different tree constructions and data characteristics. Therefore, we evaluated various machine learning models to identify the best-fit solution for a particular problem type: predicting trauma mortality for all patients visiting the ED.

Finally, we enhanced prediction performance by considering all possible combinations based on the three best models. In our final ensemble model, we found the optimal hyperparameters with a maximum depth of 6, 0.01 learning rate, and 400 tree estimators. AdaBoost’s optimal hyperparameters were 400 tree estimators, 0.1 learning rate, a maximum depth of 1, and balanced class weights. Comparatively, LightGBM’s optimal hyperparameters were a maximum depth of 3, 0.01 learning rate, and 400 tree estimators.

Our AI model has several clinical practice advantages. First, our proposed model best predicts severe trauma patients with wounds that may not survive admission or surgery, with most unable to undergo CT or magnetic resonance imaging. Moreover, our feature importance analysis indicates different prominent diagnostic codes than the previous AI model for predicting in-hospital mortality. This finding implies that ED mortality patients have distinct clinical data distributions from those with in-hospital mortality. We also used the present test data set to assess the previous in-hospital mortality AI model [5]. We discovered that the current model was superior, likely due to our current training data set’s ED mortality specificity. Second, we did not use AIS codes requiring an expert or exact diagnoses such as CT or surgery; thus, it is not a time-consuming process. All variables in our AI model are available promptly after a portable X-ray or point-of-care ultrasound. Finally, our AI model’s excellent performance would efficiently allocate medical resources. As our risk calculator tool is accessible through a mobile web app, clinicians can utilize it without time or location limitations.

We used 5,229,008 patient data sets for model training and cross-validation and 1,307,252 data sets for evaluation. To our knowledge, this is the most extensive study on developing an AI model for predicting mortality in trauma patients visiting the ED by incorporating ICD-10 codes and other clinical variables; such a large data set contributes to establishing generalization. In addition, we used SMOTE and optimized class weight search techniques to minimize an imbalanced data distribution. These techniques resolved the overfitting issue by reducing the cross-validation and testing data result difference.

Our team previously developed two AI models for predicting in-hospital mortality from various input features [4,5]. In our first study [4], we rearranged AIS codes relative to 46 anatomical regions, which was considerably more differentiated than the Injury Severity Score (ISS) system’s conventional six regions. We anticipated that the AI model would provide more appropriate weights for each anatomical organ such as the pancreas, rib, or liver. Our second study [5] used the NEDIS data set without any AIS information. Therefore, we used ICD-10 codes, procedure codes, KTAS, and other clinical variables.

We excluded ED mortality patients in both studies as they may have received insufficient diagnostic workup. Postmortem CT or autopsy is not popular in South Korea. Patients discharged from the ED and returning home may also undergo insufficient diagnosis compared to admitted patients. Additionally, some patients may die after ICU or ward admission. We defined these patients as not indicative of ED mortality. ED mortality patients may have more severe injuries that prohibit admittance. Thus, we postulated that ED mortality patients must be predicted using alternative features.

Two recent systematic reviews compared machine learning models for predicting mortality and decision support [23,24]. Zhang et al [24] discussed six studies using machine learning models based on the national database. However, their training sets ranged from 12,640 to 799,233 patients [25-30]. Our model incorporated data from over 6.5 million patients, establishing this as the largest cohort study. Moreover, our model achieved the highest performance (AUROC of 0.99) compared to previous studies with less accurate performance (AUROC of 0.89 to 0.95) [25-30]. Kim et al [26] introduced a neural network model using 408,316 patients from the National Trauma Database with a primarily ED mortality outcome. They reported an AUROC of 0.86 for the neural network using age, systolic blood pressure, respiration rate, heart rate, and GCS or simplified consciousness scores [26] and an AUROC of 0.93 when incorporating ISS. Our present outcome (AUROC of 0.99) dramatically outperforms that of the previous study. Furthermore, the ICD-10 code is more practical than the ISS regarding ED because ISS requires an expert such as a trauma coordinator, whereas any clinician can determine an ICD-10 code. In a validation study including 934,053 patients from the American College of Surgeons Trauma Quality Improvement Program database, Maure et al [29] reported an AUROC of 0.93 in penetrating injury and 0.88 in blunt injury for predicting mortality. However, they also excluded ED mortality, similar to our previous AI models [4,5].

### Limitations and Future Work

Several limitations of this study are acknowledged. First, this is a retrospective study despite the substantial quantity of patient data. Therefore, a further prospective study is needed to avoid potential selection and survival bias. Second, we did not perform external validation. Our study only used training data derived from patients in South Korea; thus, it is unclear whether our model could be adapted to other countries. In future work, we plan to conduct an external validation study using data from another country to develop a global version of the model. Third, our primary outcome was ED mortality, not in-hospital or overall mortality. Since our previous AI model [5] excluded patients with ED mortality, our two AI models would help clinicians predict various mortality types. Fourth, some data were provided as categorical variables in NEDIS, such as age. However, a sufficiently large data set may enable us to overcome this issue. Fifth, NEDIS did not include AIS or ISS, as we could not locate this information. In future work, we plan to develop an ensemble model incorporating our previously developed AIS code–based AI model [4].

### Conclusions

Our proposed AI model for predicting ED mortality achieved exceptionally high accuracy. This model is derived from a population-based data set in South Korea and provides better insight into trauma care and systems, complementing our previous AI models [4,5]. In future studies, we must consider data from various ethnic groups and integrate our previous AI models.

## Appendix

Multimedia Appendix 1 Variables included in the artificial intelligence prediction model (Table S1); summary of data sets (Table S2); ICD-10 codes between deceased and alive patients (Table S3); normalized feature importance of AdaBoost model (Table S4); survival risk ratio of ICD-10 codes (Table S5); screenshots of the web app for mortality prediction in trauma patients (Figure S1).

## Abbreviations

AdaBoost: adaptive boosting
AI: artificial intelligence
AIS: Abbreviated Injury Scale
AUROC: area under the receiver operating characteristic curve
AVPU: Alert, Verbal, Painful, Unresponsive
CT: computed tomography
DNN: deep neural network
ED: emergency department
ERT: extremely random trees
GBM: gradient boosting machine
GCS: Glasgow Coma Scale
GOSS: gradient-based one-side sampling
ICD-10: International Classification of Diseases 10th revision
ICISS: International Classification of Diseases Severity Score
ICU: intensive care unit
ISS: Injury Severity Score
KTAS: Korean Triage and Acuity Scale
LightGBM: light gradient boosting machine
LR: logistic regression
NEDIS: National Emergency Department Information System
RF: random forest
ROC: receiver operating characteristic
SMOTE: synthetic minority oversampling technique
SRR: survival risk ratio
TRIPOD: Transparent Reporting of a Multivariable Model for Individual Prognosis or Diagnosis
XGBoost: extreme gradient boosting

## Data Availability

The data are not publicly available due to National Emergency Department Information System (NEDIS) policy restrictions and belong to Korea’s National Emergency Medical Center (NEMC). The NEMC provides researchers with a deidentified NEDIS data set for nonprofit academic research. Details of accessing the raw data and guide are available at the NEMC website [31].
