# Supplementary material for: An Artificial Intelligence Model for Predicting Trauma Mortality Among Emergency Department Patients in South Korea: Retrospective Cohort Study
Source: J Med Internet Res. 2023 Aug 29;25:e49283. doi: 10.2196/49283 (PMC10498319; doi:10.2196/49283)
Supplement: Multimedia Appendix 1 [file jmir_v25i1e49283_app1.docx]

**Multimedia Appendix 1**

**Table S1. National Emergency Department Information System (NEDIS) variables for the artificial intelligence (AI) model**

| Value (n) | Variables | Type | Description |
| --- | --- | --- | --- |
| 1 | Age | 26 categories | 5-year-old unit |
| 2 | Gender | 2 categories | M: male  F: female |
| 3 | Intentionality | 5 categories | 1: accidental, unintentional  2: self-harm, suicide  3: violence, assault  4: other specified  5: unspecified |
| 4 | Injury mechanism | 16 categories | 1: car accident  2: bike accident  3: motorcycle accident-  4: other traffic accidents  5: unspecified traffic accident  6: fall  7: slipped  8: struck  9: firearm, cut, or pierced  10: machine  11: fire, flames, or heat  12: drowning  13: poisoning  14: choking, hanging  15: others  16: unknown |
| 5 | Emergent symptom | 2 categories | Y: emergency  N: non-emergency |
| 6 | AVPU^a^ scale | 4 categories | A: alert  V: verbal response (drowsy)  P: painful response (semicoma)  U: unresponsive (coma)  N: unknown |
| 7 | Initial KTAS^b^ | 7 categories | Level 1 (resuscitation)  Level 2 (emergency)  Level 3 (urgency)  Level 4 (less urgency)  Level 5 (nonurgency)  Not Classified  Missing Data |
| 8 | Systolic blood pressure | Numerical | Numerical |
| 9 | Diastolic blood pressure | Numerical | Numerical |
| 10 | Pulse rate per minute | Numerical | Numerical |
| 11 | Respiratory rate per minute | Numerical | Numerical |
| 12 | Body temperature | Numerical | Numerical |
| 13 | Oxygen saturation | Numerical | Numerical |
| 14 | Diagnostic code at discharge | 865 categories | ICD-10^c^ codes with S^d^ or T^e^ |
|  |  |  |  |
|  |  |  |  |

^a^AVPU: Alert/Verbal/Painful/Unresponsive.

^b^KTAS: Korean Triage and Acuity Scale.

^c^ICD-10: International Classification of Disease 10th Revision.

^d^Represents trauma in a single body region.

^e^Represents trauma in multiple or unspecified regions.

| **Table S2. Summary of training, validation, and testing data sets** | | | |
| --- | --- | --- | --- |
|  | **Deceased group** | **Survived group** | **Total** |
| **Training data** | 5,081 | 5,223,927 | 5,229,008 |
| **Testing data** | 1,270 | 1,305,982 | 1,307,252 |
| **Total** | 6,351 | 6,529,909 | 6,536,260 |

| **Table S3. Comparison of ICD-10 between deceased and survived patients** | | | | |
| --- | --- | --- | --- | --- |
| **no** | **ICD-10 code** | **Deceased (N=6351)** | **Survived (N=6529955)** | **p** |
| 1 | S000 | 20 (0.3%) | 113837 (1.7%) |  |
| 2 | S001 | 2 (0.0%) | 31275 (0.5%) |  |
| 3 | S002 | 0 (0.0%) | 10130 (0.2%) |  |
| 4 | S003 | 0 (0.0%) | 34883 (0.5%) |  |
| 5 | S004 | 0 (0.0%) | 9833 (0.2%) |  |
| 6 | S005 | 0 (0.0%) | 22355 (0.3%) |  |
| 7 | S007 | 1 (0.0%) | 2552 (0.0%) |  |
| 8 | S008 | 74 (1.2%) | 281647 (4.3%) |  |
| 9 | S009 | 49 (0.8%) | 93763 (1.4%) |  |
| 10 | S010 | 191 (3.0%) | 254572 (3.9%) |  |
| 11 | S011 | 13 (0.2%) | 133636 (2.0%) |  |
| 12 | S012 | 1 (0.0%) | 21804 (0.3%) |  |
| 13 | S013 | 11 (0.2%) | 26226 (0.4%) |  |
| 14 | S014 | 17 (0.3%) | 44731 (0.7%) |  |
| 15 | S015 | 8 (0.1%) | 122968 (1.9%) |  |
| 16 | S017 | 16 (0.3%) | 4098 (0.1%) |  |
| 17 | S018 | 137 (2.2%) | 366836 (5.6%) |  |
| 18 | S019 | 25 (0.4%) | 38859 (0.6%) |  |
| 19 | S020 | 361 (5.7%) | 11189 (0.2%) |  |
| 20 | S021 | 307 (4.8%) | 12610 (0.2%) |  |
| 21 | S022 | 44 (0.7%) | 77696 (1.2%) |  |
| 22 | S023 | 36 (0.6%) | 23660 (0.4%) |  |
| 23 | S024 | 112 (1.8%) | 26361 (0.4%) |  |
| 24 | S025 | 6 (0.1%) | 22031 (0.3%) |  |
| 25 | S026 | 114 (1.8%) | 11447 (0.2%) |  |
| 26 | S027 | 102 (1.6%) | 1261 (0.0%) |  |
| 27 | S028 | 186 (2.9%) | 29085 (0.4%) |  |
| 28 | S029 | 488 (7.7%) | 12663 (0.2%) |  |
| 29 | S030 | 1 (0.0%) | 7674 (0.1%) |  |
| 30 | S031 | 0 (0.0%) | 14 (0.0%) |  |
| 31 | S032 | 4 (0.1%) | 19878 (0.3%) |  |
| 32 | S033 | 0 (0.0%) | 4 (0.0%) |  |
| 33 | S034 | 0 (0.0%) | 1097 (0.0%) |  |
| 34 | S035 | 0 (0.0%) | 317 (0.0%) |  |
| 35 | S040 | 0 (0.0%) | 316 (0.0%) |  |
| 36 | S041 | 0 (0.0%) | 37 (0.0%) |  |
| 37 | S042 | 0 (0.0%) | 19 (0.0%) |  |
| 38 | S043 | 0 (0.0%) | 36 (0.0%) |  |
| 39 | S044 | 0 (0.0%) | 9 (0.0%) |  |
| 40 | S045 | 0 (0.0%) | 204 (0.0%) |  |
| 41 | S046 | 0 (0.0%) | 27 (0.0%) |  |
| 42 | S048 | 0 (0.0%) | 4 (0.0%) |  |
| 43 | S049 | 0 (0.0%) | 8 (0.0%) |  |
| 44 | S050 | 0 (0.0%) | 51321 (0.8%) |  |
| 45 | S051 | 6 (0.1%) | 31105 (0.5%) |  |
| 46 | S052 | 0 (0.0%) | 489 (0.0%) |  |
| 47 | S053 | 1 (0.0%) | 2427 (0.0%) |  |
| 48 | S054 | 0 (0.0%) | 157 (0.0%) |  |
| 49 | S055 | 0 (0.0%) | 683 (0.0%) |  |
| 50 | S056 | 0 (0.0%) | 507 (0.0%) |  |
| 51 | S057 | 0 (0.0%) | 64 (0.0%) |  |
| 52 | S058 | 0 (0.0%) | 7514 (0.1%) |  |
| 53 | S059 | 1 (0.0%) | 8179 (0.1%) |  |
| 54 | S060 | 225 (3.5%) | 553627 (8.5%) |  |
| 55 | S061 | 31 (0.5%) | 187 (0.0%) |  |
| 56 | S062 | 142 (2.2%) | 16951 (0.3%) |  |
| 57 | S063 | 53 (0.8%) | 6762 (0.1%) |  |
| 58 | S064 | 46 (0.7%) | 11406 (0.2%) |  |
| 59 | S065 | 494 (7.8%) | 53818 (0.8%) |  |
| 60 | S066 | 429 (6.8%) | 20949 (0.3%) |  |
| 61 | S067 | 6 (0.1%) | 24 (0.0%) |  |
| 62 | S068 | 172 (2.7%) | 7629 (0.1%) |  |
| 63 | S069 | 391 (6.2%) | 921 (0.0%) |  |
| 64 | S070 | 6 (0.1%) | 450 (0.0%) |  |
| 65 | S071 | 10 (0.2%) | 23 (0.0%) |  |
| 66 | S078 | 8 (0.1%) | 48 (0.0%) |  |
| 67 | S079 | 4 (0.1%) | 40 (0.0%) |  |
| 68 | S080 | 4 (0.1%) | 882 (0.0%) |  |
| 69 | S081 | 0 (0.0%) | 77 (0.0%) |  |
| 70 | S088 | 0 (0.0%) | 8 (0.0%) |  |
| 71 | S089 | 0 (0.0%) | 8 (0.0%) |  |
| 72 | S090 | 2 (0.0%) | 36 (0.0%) |  |
| 73 | S091 | 0 (0.0%) | 5124 (0.1%) |  |
| 74 | S092 | 0 (0.0%) | 3070 (0.0%) |  |
| 75 | S097 | 16 (0.3%) | 43 (0.0%) |  |
| 76 | S098 | 42 (0.7%) | 2095 (0.0%) |  |
| 77 | S099 | 76 (1.2%) | 46114 (0.7%) |  |
| 78 | S100 | 2 (0.0%) | 781 (0.0%) |  |
| 79 | S101 | 1 (0.0%) | 2304 (0.0%) |  |
| 80 | S107 | 0 (0.0%) | 169 (0.0%) |  |
| 81 | S108 | 9 (0.1%) | 9857 (0.2%) |  |
| 82 | S109 | 16 (0.3%) | 16889 (0.3%) |  |
| 83 | S110 | 5 (0.1%) | 73 (0.0%) |  |
| 84 | S111 | 0 (0.0%) | 7 (0.0%) |  |
| 85 | S112 | 0 (0.0%) | 75 (0.0%) |  |
| 86 | S117 | 3 (0.0%) | 223 (0.0%) |  |
| 87 | S118 | 7 (0.1%) | 1181 (0.0%) |  |
| 88 | S119 | 19 (0.3%) | 2645 (0.0%) |  |
| 89 | S120 | 14 (0.2%) | 613 (0.0%) |  |
| 90 | S121 | 24 (0.4%) | 1479 (0.0%) |  |
| 91 | S122 | 32 (0.5%) | 2714 (0.0%) |  |
| 92 | S127 | 34 (0.5%) | 1056 (0.0%) |  |
| 93 | S128 | 5 (0.1%) | 256 (0.0%) |  |
| 94 | S129 | 119 (1.9%) | 6246 (0.1%) |  |
| 95 | S130 | 1 (0.0%) | 380 (0.0%) |  |
| 96 | S131 | 64 (1.0%) | 892 (0.0%) |  |
| 97 | S132 | 0 (0.0%) | 11 (0.0%) |  |
| 98 | S133 | 0 (0.0%) | 6 (0.0%) |  |
| 99 | S134 | 30 (0.5%) | 361329 (5.5%) |  |
| 100 | S135 | 0 (0.0%) | 28 (0.0%) |  |
| 101 | S136 | 2 (0.0%) | 26876 (0.4%) |  |
| 102 | S140 | 2 (0.0%) | 562 (0.0%) |  |
| 103 | S141 | 26 (0.4%) | 4077 (0.1%) |  |
| 104 | S142 | 5 (0.1%) | 210 (0.0%) |  |
| 105 | S143 | 0 (0.0%) | 202 (0.0%) |  |
| 106 | S144 | 0 (0.0%) | 5 (0.0%) |  |
| 107 | S146 | 1 (0.0%) | 80 (0.0%) |  |
| 108 | S150 | 7 (0.1%) | 26 (0.0%) |  |
| 109 | S151 | 2 (0.0%) | 25 (0.0%) |  |
| 110 | S152 | 2 (0.0%) | 14 (0.0%) |  |
| 111 | S153 | 0 (0.0%) | 17 (0.0%) |  |
| 112 | S157 | 0 (0.0%) | 11 (0.0%) |  |
| 113 | S158 | 2 (0.0%) | 115 (0.0%) |  |
| 114 | S159 | 4 (0.1%) | 127 (0.0%) |  |
| 115 | S160 | 1 (0.0%) | 113 (0.0%) |  |
| 116 | S170 | 6 (0.1%) | 14 (0.0%) |  |
| 117 | S178 | 1 (0.0%) | 2 (0.0%) |  |
| 118 | S179 | 1 (0.0%) | 16 (0.0%) |  |
| 119 | S180 | 0 (0.0%) | 3 (0.0%) |  |
| 120 | S190 | 0 (0.0%) | 4 (0.0%) |  |
| 121 | S197 | 2 (0.0%) | 13 (0.0%) |  |
| 122 | S198 | 3 (0.0%) | 278 (0.0%) |  |
| 123 | S199 | 17 (0.3%) | 986 (0.0%) |  |
| 124 | S200 | 0 (0.0%) | 171 (0.0%) |  |
| 125 | S201 | 0 (0.0%) | 84 (0.0%) |  |
| 126 | S202 | 125 (2.0%) | 188093 (2.9%) |  |
| 127 | S203 | 4 (0.1%) | 1305 (0.0%) |  |
| 128 | S204 | 0 (0.0%) | 1043 (0.0%) |  |
| 129 | S207 | 0 (0.0%) | 108 (0.0%) |  |
| 130 | S208 | 7 (0.1%) | 5987 (0.1%) |  |
| 131 | S210 | 0 (0.0%) | 108 (0.0%) |  |
| 132 | S211 | 17 (0.3%) | 1229 (0.0%) |  |
| 133 | S212 | 3 (0.0%) | 1313 (0.0%) |  |
| 134 | S217 | 2 (0.0%) | 46 (0.0%) |  |
| 135 | S218 | 3 (0.0%) | 252 (0.0%) |  |
| 136 | S219 | 20 (0.3%) | 1066 (0.0%) |  |
| 137 | S220 | 97 (1.5%) | 29318 (0.4%) |  |
| 138 | S221 | 18 (0.3%) | 1626 (0.0%) |  |
| 139 | S222 | 60 (0.9%) | 10360 (0.2%) |  |
| 140 | S223 | 127 (2.0%) | 48949 (0.7%) |  |
| 141 | S224 | 1734 (27.3%) | 70763 (1.1%) |  |
| 142 | S225 | 73 (1.1%) | 408 (0.0%) |  |
| 143 | S228 | 3 (0.0%) | 393 (0.0%) |  |
| 144 | S229 | 4 (0.1%) | 269 (0.0%) |  |
| 145 | S230 | 0 (0.0%) | 22 (0.0%) |  |
| 146 | S231 | 4 (0.1%) | 83 (0.0%) |  |
| 147 | S232 | 0 (0.0%) | 21 (0.0%) |  |
| 148 | S233 | 0 (0.0%) | 15952 (0.2%) |  |
| 149 | S234 | 0 (0.0%) | 9395 (0.1%) |  |
| 150 | S235 | 0 (0.0%) | 3729 (0.1%) |  |
| 151 | S240 | 0 (0.0%) | 20 (0.0%) |  |
| 152 | S241 | 1 (0.0%) | 155 (0.0%) |  |
| 153 | S242 | 0 (0.0%) | 33 (0.0%) |  |
| 154 | S244 | 0 (0.0%) | 7 (0.0%) |  |
| 155 | S245 | 0 (0.0%) | 1 (0.0%) |  |
| 156 | S246 | 1 (0.0%) | 6 (0.0%) |  |
| 157 | S250 | 63 (1.0%) | 286 (0.0%) |  |
| 158 | S251 | 2 (0.0%) | 19 (0.0%) |  |
| 159 | S252 | 2 (0.0%) | 4 (0.0%) |  |
| 160 | S253 | 0 (0.0%) | 10 (0.0%) |  |
| 161 | S254 | 6 (0.1%) | 26 (0.0%) |  |
| 162 | S255 | 2 (0.0%) | 32 (0.0%) |  |
| 163 | S257 | 2 (0.0%) | 0 (0.0%) |  |
| 164 | S258 | 7 (0.1%) | 62 (0.0%) |  |
| 165 | S259 | 5 (0.1%) | 40 (0.0%) |  |
| 166 | S260 | 55 (0.9%) | 161 (0.0%) |  |
| 167 | S268 | 25 (0.4%) | 367 (0.0%) |  |
| 168 | S269 | 55 (0.9%) | 302 (0.0%) |  |
| 169 | S270 | 391 (6.2%) | 7373 (0.1%) |  |
| 170 | S271 | 572 (9.0%) | 6862 (0.1%) |  |
| 171 | S272 | 768 (12.1%) | 5764 (0.1%) |  |
| 172 | S273 | 249 (3.9%) | 6022 (0.1%) |  |
| 173 | S274 | 3 (0.0%) | 24 (0.0%) |  |
| 174 | S275 | 16 (0.3%) | 167 (0.0%) |  |
| 175 | S276 | 1 (0.0%) | 47 (0.0%) |  |
| 176 | S277 | 5 (0.1%) | 35 (0.0%) |  |
| 177 | S278 | 38 (0.6%) | 520 (0.0%) |  |
| 178 | S279 | 11 (0.2%) | 70 (0.0%) |  |
| 179 | S280 | 22 (0.3%) | 10 (0.0%) |  |
| 180 | S281 | 1 (0.0%) | 17 (0.0%) |  |
| 181 | S290 | 0 (0.0%) | 54 (0.0%) |  |
| 182 | S297 | 12 (0.2%) | 12 (0.0%) |  |
| 183 | S298 | 3 (0.0%) | 198 (0.0%) |  |
| 184 | S299 | 35 (0.6%) | 851 (0.0%) |  |
| 185 | S300 | 23 (0.4%) | 94374 (1.4%) |  |
| 186 | S301 | 81 (1.3%) | 50775 (0.8%) |  |
| 187 | S302 | 1 (0.0%) | 6113 (0.1%) |  |
| 188 | S307 | 0 (0.0%) | 247 (0.0%) |  |
| 189 | S308 | 4 (0.1%) | 10091 (0.2%) |  |
| 190 | S309 | 4 (0.1%) | 1742 (0.0%) |  |
| 191 | S310 | 11 (0.2%) | 4225 (0.1%) |  |
| 192 | S311 | 27 (0.4%) | 3454 (0.1%) |  |
| 193 | S312 | 0 (0.0%) | 1541 (0.0%) |  |
| 194 | S313 | 3 (0.0%) | 1633 (0.0%) |  |
| 195 | S314 | 1 (0.0%) | 1466 (0.0%) |  |
| 196 | S315 | 1 (0.0%) | 272 (0.0%) |  |
| 197 | S317 | 2 (0.0%) | 139 (0.0%) |  |
| 198 | S318 | 22 (0.3%) | 1502 (0.0%) |  |
| 199 | S320 | 112 (1.8%) | 60725 (0.9%) |  |
| 200 | S321 | 61 (1.0%) | 5147 (0.1%) |  |
| 201 | S322 | 1 (0.0%) | 6348 (0.1%) |  |
| 202 | S323 | 36 (0.6%) | 2251 (0.0%) |  |
| 203 | S324 | 43 (0.7%) | 4813 (0.1%) |  |
| 204 | S325 | 115 (1.8%) | 6977 (0.1%) |  |
| 205 | S327 | 63 (1.0%) | 2355 (0.0%) |  |
| 206 | S328 | 969 (15.3%) | 17476 (0.3%) |  |
| 207 | S330 | 0 (0.0%) | 266 (0.0%) |  |
| 208 | S331 | 0 (0.0%) | 209 (0.0%) |  |
| 209 | S332 | 2 (0.0%) | 101 (0.0%) |  |
| 210 | S333 | 3 (0.0%) | 139 (0.0%) |  |
| 211 | S334 | 3 (0.0%) | 94 (0.0%) |  |
| 212 | S335 | 4 (0.1%) | 271104 (4.2%) |  |
| 213 | S336 | 3 (0.0%) | 421 (0.0%) |  |
| 214 | S337 | 0 (0.0%) | 16384 (0.3%) |  |
| 215 | S340 | 0 (0.0%) | 55 (0.0%) |  |
| 216 | S341 | 1 (0.0%) | 443 (0.0%) |  |
| 217 | S342 | 1 (0.0%) | 57 (0.0%) |  |
| 218 | S343 | 0 (0.0%) | 16 (0.0%) |  |
| 219 | S344 | 0 (0.0%) | 10 (0.0%) |  |
| 220 | S345 | 0 (0.0%) | 23 (0.0%) |  |
| 221 | S346 | 0 (0.0%) | 3 (0.0%) |  |
| 222 | S348 | 0 (0.0%) | 17 (0.0%) |  |
| 223 | S350 | 2 (0.0%) | 69 (0.0%) |  |
| 224 | S351 | 12 (0.2%) | 92 (0.0%) |  |
| 225 | S352 | 17 (0.3%) | 413 (0.0%) |  |
| 226 | S353 | 6 (0.1%) | 134 (0.0%) |  |
| 227 | S354 | 7 (0.1%) | 181 (0.0%) |  |
| 228 | S355 | 7 (0.1%) | 132 (0.0%) |  |
| 229 | S357 | 4 (0.1%) | 26 (0.0%) |  |
| 230 | S358 | 10 (0.2%) | 235 (0.0%) |  |
| 231 | S359 | 7 (0.1%) | 271 (0.0%) |  |
| 232 | S360 | 73 (1.1%) | 3675 (0.1%) |  |
| 233 | S361 | 157 (2.5%) | 7024 (0.1%) |  |
| 234 | S362 | 10 (0.2%) | 589 (0.0%) |  |
| 235 | S363 | 3 (0.0%) | 77 (0.0%) |  |
| 236 | S364 | 4 (0.1%) | 631 (0.0%) |  |
| 237 | S365 | 2 (0.0%) | 257 (0.0%) |  |
| 238 | S366 | 0 (0.0%) | 160 (0.0%) |  |
| 239 | S367 | 19 (0.3%) | 110 (0.0%) |  |
| 240 | S368 | 176 (2.8%) | 3231 (0.0%) |  |
| 241 | S369 | 47 (0.7%) | 704 (0.0%) |  |
| 242 | S370 | 59 (0.9%) | 3043 (0.0%) |  |
| 243 | S371 | 0 (0.0%) | 90 (0.0%) |  |
| 244 | S372 | 16 (0.3%) | 555 (0.0%) |  |
| 245 | S373 | 4 (0.1%) | 1855 (0.0%) |  |
| 246 | S374 | 0 (0.0%) | 11 (0.0%) |  |
| 247 | S375 | 0 (0.0%) | 2 (0.0%) |  |
| 248 | S376 | 0 (0.0%) | 22 (0.0%) |  |
| 249 | S377 | 5 (0.1%) | 32 (0.0%) |  |
| 250 | S378 | 11 (0.2%) | 470 (0.0%) |  |
| 251 | S379 | 2 (0.0%) | 119 (0.0%) |  |
| 252 | S380 | 0 (0.0%) | 59 (0.0%) |  |
| 253 | S381 | 0 (0.0%) | 25 (0.0%) |  |
| 254 | S382 | 0 (0.0%) | 32 (0.0%) |  |
| 255 | S383 | 0 (0.0%) | 1 (0.0%) |  |
| 256 | S390 | 2 (0.0%) | 194 (0.0%) |  |
| 257 | S396 | 8 (0.1%) | 40 (0.0%) |  |
| 258 | S397 | 6 (0.1%) | 29 (0.0%) |  |
| 259 | S398 | 1 (0.0%) | 254 (0.0%) |  |
| 260 | S399 | 18 (0.3%) | 3476 (0.1%) |  |
| 261 | S400 | 11 (0.2%) | 138850 (2.1%) |  |
| 262 | S407 | 1 (0.0%) | 307 (0.0%) |  |
| 263 | S408 | 5 (0.1%) | 4905 (0.1%) |  |
| 264 | S409 | 0 (0.0%) | 1292 (0.0%) |  |
| 265 | S410 | 4 (0.1%) | 1876 (0.0%) |  |
| 266 | S411 | 8 (0.1%) | 5808 (0.1%) |  |
| 267 | S417 | 1 (0.0%) | 546 (0.0%) |  |
| 268 | S418 | 0 (0.0%) | 193 (0.0%) |  |
| 269 | S420 | 178 (2.8%) | 46519 (0.7%) |  |
| 270 | S421 | 85 (1.3%) | 9475 (0.1%) |  |
| 271 | S422 | 31 (0.5%) | 20094 (0.3%) |  |
| 272 | S423 | 178 (2.8%) | 17448 (0.3%) |  |
| 273 | S424 | 14 (0.2%) | 21047 (0.3%) |  |
| 274 | S427 | 2 (0.0%) | 75 (0.0%) |  |
| 275 | S428 | 6 (0.1%) | 474 (0.0%) |  |
| 276 | S429 | 1 (0.0%) | 881 (0.0%) |  |
| 277 | S430 | 5 (0.1%) | 25239 (0.4%) |  |
| 278 | S431 | 0 (0.0%) | 1730 (0.0%) |  |
| 279 | S432 | 0 (0.0%) | 92 (0.0%) |  |
| 280 | S433 | 0 (0.0%) | 1946 (0.0%) |  |
| 281 | S434 | 0 (0.0%) | 33417 (0.5%) |  |
| 282 | S435 | 0 (0.0%) | 2385 (0.0%) |  |
| 283 | S436 | 0 (0.0%) | 79 (0.0%) |  |
| 284 | S437 | 0 (0.0%) | 8867 (0.1%) |  |
| 285 | S440 | 0 (0.0%) | 18 (0.0%) |  |
| 286 | S441 | 0 (0.0%) | 12 (0.0%) |  |
| 287 | S442 | 0 (0.0%) | 62 (0.0%) |  |
| 288 | S443 | 0 (0.0%) | 154 (0.0%) |  |
| 289 | S444 | 0 (0.0%) | 14 (0.0%) |  |
| 290 | S445 | 0 (0.0%) | 4 (0.0%) |  |
| 291 | S447 | 0 (0.0%) | 4 (0.0%) |  |
| 292 | S448 | 0 (0.0%) | 15 (0.0%) |  |
| 293 | S449 | 0 (0.0%) | 29 (0.0%) |  |
| 294 | S450 | 0 (0.0%) | 24 (0.0%) |  |
| 295 | S451 | 0 (0.0%) | 77 (0.0%) |  |
| 296 | S452 | 0 (0.0%) | 24 (0.0%) |  |
| 297 | S453 | 0 (0.0%) | 67 (0.0%) |  |
| 298 | S457 | 0 (0.0%) | 4 (0.0%) |  |
| 299 | S458 | 1 (0.0%) | 96 (0.0%) |  |
| 300 | S459 | 1 (0.0%) | 73 (0.0%) |  |
| 301 | S460 | 0 (0.0%) | 1145 (0.0%) |  |
| 302 | S461 | 0 (0.0%) | 129 (0.0%) |  |
| 303 | S462 | 0 (0.0%) | 173 (0.0%) |  |
| 304 | S463 | 0 (0.0%) | 110 (0.0%) |  |
| 305 | S467 | 1 (0.0%) | 189 (0.0%) |  |
| 306 | S468 | 0 (0.0%) | 439 (0.0%) |  |
| 307 | S469 | 0 (0.0%) | 433 (0.0%) |  |
| 308 | S470 | 0 (0.0%) | 87 (0.0%) |  |
| 309 | S480 | 0 (0.0%) | 117 (0.0%) |  |
| 310 | S481 | 0 (0.0%) | 23 (0.0%) |  |
| 311 | S489 | 2 (0.0%) | 54 (0.0%) |  |
| 312 | S490 | 0 (0.0%) | 3 (0.0%) |  |
| 313 | S497 | 0 (0.0%) | 28 (0.0%) |  |
| 314 | S498 | 0 (0.0%) | 278 (0.0%) |  |
| 315 | S499 | 0 (0.0%) | 2337 (0.0%) |  |
| 316 | S500 | 4 (0.1%) | 104712 (1.6%) |  |
| 317 | S501 | 4 (0.1%) | 37671 (0.6%) |  |
| 318 | S507 | 0 (0.0%) | 1381 (0.0%) |  |
| 319 | S508 | 4 (0.1%) | 14560 (0.2%) |  |
| 320 | S509 | 1 (0.0%) | 5985 (0.1%) |  |
| 321 | S510 | 9 (0.1%) | 15435 (0.2%) |  |
| 322 | S517 | 2 (0.0%) | 3106 (0.0%) |  |
| 323 | S518 | 10 (0.2%) | 12838 (0.2%) |  |
| 324 | S519 | 13 (0.2%) | 24557 (0.4%) |  |
| 325 | S520 | 31 (0.5%) | 16753 (0.3%) |  |
| 326 | S521 | 14 (0.2%) | 12993 (0.2%) |  |
| 327 | S522 | 11 (0.2%) | 4139 (0.1%) |  |
| 328 | S523 | 10 (0.2%) | 6052 (0.1%) |  |
| 329 | S524 | 25 (0.4%) | 4293 (0.1%) |  |
| 330 | S525 | 18 (0.3%) | 76818 (1.2%) |  |
| 331 | S526 | 17 (0.3%) | 15322 (0.2%) |  |
| 332 | S527 | 8 (0.1%) | 663 (0.0%) |  |
| 333 | S528 | 15 (0.2%) | 8360 (0.1%) |  |
| 334 | S529 | 27 (0.4%) | 3556 (0.1%) |  |
| 335 | S530 | 0 (0.0%) | 44606 (0.7%) |  |
| 336 | S531 | 3 (0.0%) | 43433 (0.7%) |  |
| 337 | S532 | 0 (0.0%) | 89 (0.0%) |  |
| 338 | S533 | 0 (0.0%) | 135 (0.0%) |  |
| 339 | S534 | 0 (0.0%) | 18203 (0.3%) |  |
| 340 | S540 | 0 (0.0%) | 192 (0.0%) |  |
| 341 | S541 | 0 (0.0%) | 115 (0.0%) |  |
| 342 | S542 | 0 (0.0%) | 331 (0.0%) |  |
| 343 | S543 | 0 (0.0%) | 26 (0.0%) |  |
| 344 | S547 | 0 (0.0%) | 6 (0.0%) |  |
| 345 | S548 | 0 (0.0%) | 28 (0.0%) |  |
| 346 | S549 | 0 (0.0%) | 139 (0.0%) |  |
| 347 | S550 | 0 (0.0%) | 67 (0.0%) |  |
| 348 | S551 | 0 (0.0%) | 162 (0.0%) |  |
| 349 | S552 | 0 (0.0%) | 111 (0.0%) |  |
| 350 | S557 | 0 (0.0%) | 10 (0.0%) |  |
| 351 | S558 | 0 (0.0%) | 473 (0.0%) |  |
| 352 | S559 | 0 (0.0%) | 258 (0.0%) |  |
| 353 | S560 | 0 (0.0%) | 175 (0.0%) |  |
| 354 | S561 | 0 (0.0%) | 712 (0.0%) |  |
| 355 | S562 | 0 (0.0%) | 626 (0.0%) |  |
| 356 | S563 | 0 (0.0%) | 249 (0.0%) |  |
| 357 | S564 | 0 (0.0%) | 1197 (0.0%) |  |
| 358 | S565 | 0 (0.0%) | 361 (0.0%) |  |
| 359 | S567 | 0 (0.0%) | 308 (0.0%) |  |
| 360 | S568 | 0 (0.0%) | 940 (0.0%) |  |
| 361 | S570 | 0 (0.0%) | 127 (0.0%) |  |
| 362 | S578 | 0 (0.0%) | 116 (0.0%) |  |
| 363 | S579 | 2 (0.0%) | 169 (0.0%) |  |
| 364 | S580 | 0 (0.0%) | 59 (0.0%) |  |
| 365 | S581 | 1 (0.0%) | 18 (0.0%) |  |
| 366 | S589 | 2 (0.0%) | 67 (0.0%) |  |
| 367 | S590 | 0 (0.0%) | 7 (0.0%) |  |
| 368 | S597 | 0 (0.0%) | 49 (0.0%) |  |
| 369 | S598 | 0 (0.0%) | 250 (0.0%) |  |
| 370 | S599 | 3 (0.0%) | 3294 (0.1%) |  |
| 371 | S600 | 0 (0.0%) | 101075 (1.5%) |  |
| 372 | S601 | 0 (0.0%) | 15030 (0.2%) |  |
| 373 | S602 | 7 (0.1%) | 137372 (2.1%) |  |
| 374 | S607 | 0 (0.0%) | 1822 (0.0%) |  |
| 375 | S608 | 5 (0.1%) | 42712 (0.7%) |  |
| 376 | S609 | 0 (0.0%) | 17787 (0.3%) |  |
| 377 | S610 | 4 (0.1%) | 389718 (6.0%) |  |
| 378 | S611 | 1 (0.0%) | 41040 (0.6%) |  |
| 379 | S617 | 4 (0.1%) | 10260 (0.2%) |  |
| 380 | S618 | 10 (0.2%) | 73375 (1.1%) |  |
| 381 | S619 | 13 (0.2%) | 88489 (1.4%) |  |
| 382 | S620 | 0 (0.0%) | 2682 (0.0%) |  |
| 383 | S621 | 3 (0.0%) | 4997 (0.1%) |  |
| 384 | S622 | 0 (0.0%) | 2284 (0.0%) |  |
| 385 | S623 | 5 (0.1%) | 22028 (0.3%) |  |
| 386 | S624 | 1 (0.0%) | 1069 (0.0%) |  |
| 387 | S625 | 1 (0.0%) | 8255 (0.1%) |  |
| 388 | S626 | 6 (0.1%) | 70522 (1.1%) |  |
| 389 | S627 | 1 (0.0%) | 2193 (0.0%) |  |
| 390 | S628 | 22 (0.3%) | 10502 (0.2%) |  |
| 391 | S630 | 2 (0.0%) | 1455 (0.0%) |  |
| 392 | S631 | 2 (0.0%) | 8370 (0.1%) |  |
| 393 | S632 | 0 (0.0%) | 72 (0.0%) |  |
| 394 | S633 | 0 (0.0%) | 235 (0.0%) |  |
| 395 | S634 | 0 (0.0%) | 853 (0.0%) |  |
| 396 | S635 | 0 (0.0%) | 42742 (0.7%) |  |
| 397 | S636 | 1 (0.0%) | 25479 (0.4%) |  |
| 398 | S637 | 0 (0.0%) | 5701 (0.1%) |  |
| 399 | S640 | 0 (0.0%) | 116 (0.0%) |  |
| 400 | S641 | 0 (0.0%) | 135 (0.0%) |  |
| 401 | S642 | 0 (0.0%) | 122 (0.0%) |  |
| 402 | S643 | 0 (0.0%) | 244 (0.0%) |  |
| 403 | S644 | 0 (0.0%) | 1157 (0.0%) |  |
| 404 | S647 | 0 (0.0%) | 13 (0.0%) |  |
| 405 | S648 | 0 (0.0%) | 110 (0.0%) |  |
| 406 | S649 | 0 (0.0%) | 142 (0.0%) |  |
| 407 | S650 | 0 (0.0%) | 155 (0.0%) |  |
| 408 | S651 | 1 (0.0%) | 209 (0.0%) |  |
| 409 | S652 | 0 (0.0%) | 589 (0.0%) |  |
| 410 | S653 | 0 (0.0%) | 176 (0.0%) |  |
| 411 | S654 | 0 (0.0%) | 760 (0.0%) |  |
| 412 | S655 | 0 (0.0%) | 9268 (0.1%) |  |
| 413 | S657 | 0 (0.0%) | 15 (0.0%) |  |
| 414 | S658 | 1 (0.0%) | 1716 (0.0%) |  |
| 415 | S659 | 0 (0.0%) | 988 (0.0%) |  |
| 416 | S660 | 0 (0.0%) | 460 (0.0%) |  |
| 417 | S661 | 0 (0.0%) | 3585 (0.1%) |  |
| 418 | S662 | 0 (0.0%) | 2147 (0.0%) |  |
| 419 | S663 | 0 (0.0%) | 10365 (0.2%) |  |
| 420 | S664 | 0 (0.0%) | 271 (0.0%) |  |
| 421 | S665 | 0 (0.0%) | 1176 (0.0%) |  |
| 422 | S666 | 0 (0.0%) | 489 (0.0%) |  |
| 423 | S667 | 0 (0.0%) | 508 (0.0%) |  |
| 424 | S668 | 1 (0.0%) | 1802 (0.0%) |  |
| 425 | S669 | 1 (0.0%) | 2701 (0.0%) |  |
| 426 | S670 | 0 (0.0%) | 7635 (0.1%) |  |
| 427 | S678 | 2 (0.0%) | 1272 (0.0%) |  |
| 428 | S680 | 1 (0.0%) | 936 (0.0%) |  |
| 429 | S681 | 0 (0.0%) | 6308 (0.1%) |  |
| 430 | S682 | 0 (0.0%) | 663 (0.0%) |  |
| 431 | S683 | 1 (0.0%) | 502 (0.0%) |  |
| 432 | S684 | 0 (0.0%) | 63 (0.0%) |  |
| 433 | S688 | 2 (0.0%) | 186 (0.0%) |  |
| 434 | S689 | 1 (0.0%) | 213 (0.0%) |  |
| 435 | S690 | 0 (0.0%) | 5 (0.0%) |  |
| 436 | S697 | 1 (0.0%) | 178 (0.0%) |  |
| 437 | S698 | 0 (0.0%) | 913 (0.0%) |  |
| 438 | S699 | 3 (0.0%) | 19102 (0.3%) |  |
| 439 | S700 | 14 (0.2%) | 77173 (1.2%) |  |
| 440 | S701 | 5 (0.1%) | 28270 (0.4%) |  |
| 441 | S707 | 2 (0.0%) | 568 (0.0%) |  |
| 442 | S708 | 3 (0.0%) | 4722 (0.1%) |  |
| 443 | S709 | 3 (0.0%) | 1719 (0.0%) |  |
| 444 | S710 | 1 (0.0%) | 1712 (0.0%) |  |
| 445 | S711 | 11 (0.2%) | 12523 (0.2%) |  |
| 446 | S717 | 1 (0.0%) | 340 (0.0%) |  |
| 447 | S718 | 3 (0.0%) | 154 (0.0%) |  |
| 448 | S720 | 82 (1.3%) | 40993 (0.6%) |  |
| 449 | S721 | 57 (0.9%) | 42456 (0.7%) |  |
| 450 | S722 | 17 (0.3%) | 2508 (0.0%) |  |
| 451 | S723 | 149 (2.3%) | 10731 (0.2%) |  |
| 452 | S724 | 27 (0.4%) | 5938 (0.1%) |  |
| 453 | S727 | 22 (0.3%) | 191 (0.0%) |  |
| 454 | S728 | 43 (0.7%) | 2937 (0.0%) |  |
| 455 | S729 | 175 (2.8%) | 16231 (0.2%) |  |
| 456 | S730 | 23 (0.4%) | 5539 (0.1%) |  |
| 457 | S731 | 0 (0.0%) | 9577 (0.1%) |  |
| 458 | S740 | 0 (0.0%) | 52 (0.0%) |  |
| 459 | S741 | 0 (0.0%) | 13 (0.0%) |  |
| 460 | S747 | 0 (0.0%) | 2 (0.0%) |  |
| 461 | S748 | 0 (0.0%) | 11 (0.0%) |  |
| 462 | S749 | 0 (0.0%) | 7 (0.0%) |  |
| 463 | S750 | 7 (0.1%) | 117 (0.0%) |  |
| 464 | S751 | 2 (0.0%) | 56 (0.0%) |  |
| 465 | S752 | 0 (0.0%) | 5 (0.0%) |  |
| 466 | S757 | 1 (0.0%) | 6 (0.0%) |  |
| 467 | S758 | 0 (0.0%) | 192 (0.0%) |  |
| 468 | S759 | 2 (0.0%) | 214 (0.0%) |  |
| 469 | S760 | 0 (0.0%) | 196 (0.0%) |  |
| 470 | S761 | 0 (0.0%) | 1322 (0.0%) |  |
| 471 | S762 | 0 (0.0%) | 149 (0.0%) |  |
| 472 | S763 | 0 (0.0%) | 329 (0.0%) |  |
| 473 | S764 | 1 (0.0%) | 882 (0.0%) |  |
| 474 | S767 | 0 (0.0%) | 202 (0.0%) |  |
| 475 | S770 | 0 (0.0%) | 13 (0.0%) |  |
| 476 | S771 | 2 (0.0%) | 81 (0.0%) |  |
| 477 | S772 | 1 (0.0%) | 15 (0.0%) |  |
| 478 | S780 | 1 (0.0%) | 1 (0.0%) |  |
| 479 | S781 | 0 (0.0%) | 13 (0.0%) |  |
| 480 | S789 | 3 (0.0%) | 6 (0.0%) |  |
| 481 | S790 | 0 (0.0%) | 1 (0.0%) |  |
| 482 | S797 | 0 (0.0%) | 16 (0.0%) |  |
| 483 | S798 | 1 (0.0%) | 146 (0.0%) |  |
| 484 | S799 | 1 (0.0%) | 1438 (0.0%) |  |
| 485 | S800 | 11 (0.2%) | 213753 (3.3%) |  |
| 486 | S801 | 13 (0.2%) | 79338 (1.2%) |  |
| 487 | S807 | 3 (0.0%) | 2557 (0.0%) |  |
| 488 | S808 | 3 (0.0%) | 28585 (0.4%) |  |
| 489 | S809 | 2 (0.0%) | 6681 (0.1%) |  |
| 490 | S810 | 22 (0.3%) | 45520 (0.7%) |  |
| 491 | S817 | 6 (0.1%) | 2617 (0.0%) |  |
| 492 | S818 | 17 (0.3%) | 21533 (0.3%) |  |
| 493 | S819 | 21 (0.3%) | 34553 (0.5%) |  |
| 494 | S820 | 14 (0.2%) | 18246 (0.3%) |  |
| 495 | S821 | 44 (0.7%) | 11938 (0.2%) |  |
| 496 | S822 | 169 (2.7%) | 18714 (0.3%) |  |
| 497 | S823 | 72 (1.1%) | 15529 (0.2%) |  |
| 498 | S824 | 42 (0.7%) | 15146 (0.2%) |  |
| 499 | S825 | 30 (0.5%) | 9212 (0.1%) |  |
| 500 | S826 | 8 (0.1%) | 24756 (0.4%) |  |
| 501 | S827 | 18 (0.3%) | 303 (0.0%) |  |
| 502 | S828 | 55 (0.9%) | 19512 (0.3%) |  |
| 503 | S829 | 30 (0.5%) | 2134 (0.0%) |  |
| 504 | S830 | 0 (0.0%) | 1401 (0.0%) |  |
| 505 | S831 | 0 (0.0%) | 1020 (0.0%) |  |
| 506 | S832 | 0 (0.0%) | 1471 (0.0%) |  |
| 507 | S833 | 0 (0.0%) | 57 (0.0%) |  |
| 508 | S834 | 0 (0.0%) | 2816 (0.0%) |  |
| 509 | S835 | 0 (0.0%) | 2520 (0.0%) |  |
| 510 | S836 | 1 (0.0%) | 37966 (0.6%) |  |
| 511 | S837 | 0 (0.0%) | 1045 (0.0%) |  |
| 512 | S840 | 0 (0.0%) | 32 (0.0%) |  |
| 513 | S841 | 0 (0.0%) | 117 (0.0%) |  |
| 514 | S842 | 0 (0.0%) | 11 (0.0%) |  |
| 515 | S848 | 0 (0.0%) | 17 (0.0%) |  |
| 516 | S849 | 0 (0.0%) | 15 (0.0%) |  |
| 517 | S850 | 0 (0.0%) | 378 (0.0%) |  |
| 518 | S851 | 0 (0.0%) | 107 (0.0%) |  |
| 519 | S852 | 0 (0.0%) | 8 (0.0%) |  |
| 520 | S853 | 0 (0.0%) | 11 (0.0%) |  |
| 521 | S854 | 0 (0.0%) | 5 (0.0%) |  |
| 522 | S855 | 0 (0.0%) | 14 (0.0%) |  |
| 523 | S857 | 1 (0.0%) | 15 (0.0%) |  |
| 524 | S858 | 0 (0.0%) | 742 (0.0%) |  |
| 525 | S859 | 0 (0.0%) | 576 (0.0%) |  |
| 526 | S860 | 0 (0.0%) | 7735 (0.1%) |  |
| 527 | S861 | 0 (0.0%) | 1150 (0.0%) |  |
| 528 | S862 | 0 (0.0%) | 467 (0.0%) |  |
| 529 | S863 | 0 (0.0%) | 279 (0.0%) |  |
| 530 | S867 | 2 (0.0%) | 583 (0.0%) |  |
| 531 | S868 | 0 (0.0%) | 1768 (0.0%) |  |
| 532 | S869 | 2 (0.0%) | 2422 (0.0%) |  |
| 533 | S870 | 1 (0.0%) | 338 (0.0%) |  |
| 534 | S878 | 12 (0.2%) | 372 (0.0%) |  |
| 535 | S880 | 2 (0.0%) | 26 (0.0%) |  |
| 536 | S881 | 2 (0.0%) | 49 (0.0%) |  |
| 537 | S889 | 4 (0.1%) | 218 (0.0%) |  |
| 538 | S890 | 0 (0.0%) | 1 (0.0%) |  |
| 539 | S897 | 1 (0.0%) | 54 (0.0%) |  |
| 540 | S898 | 0 (0.0%) | 453 (0.0%) |  |
| 541 | S899 | 5 (0.1%) | 5298 (0.1%) |  |
| 542 | S900 | 4 (0.1%) | 53663 (0.8%) |  |
| 543 | S901 | 0 (0.0%) | 36846 (0.6%) |  |
| 544 | S902 | 0 (0.0%) | 14015 (0.2%) |  |
| 545 | S903 | 1 (0.0%) | 82233 (1.3%) |  |
| 546 | S907 | 0 (0.0%) | 905 (0.0%) |  |
| 547 | S908 | 3 (0.0%) | 19591 (0.3%) |  |
| 548 | S909 | 0 (0.0%) | 6846 (0.1%) |  |
| 549 | S910 | 12 (0.2%) | 12506 (0.2%) |  |
| 550 | S911 | 1 (0.0%) | 24965 (0.4%) |  |
| 551 | S912 | 0 (0.0%) | 7902 (0.1%) |  |
| 552 | S913 | 8 (0.1%) | 58796 (0.9%) |  |
| 553 | S917 | 0 (0.0%) | 1536 (0.0%) |  |
| 554 | S920 | 12 (0.2%) | 15732 (0.2%) |  |
| 555 | S921 | 4 (0.1%) | 2965 (0.0%) |  |
| 556 | S922 | 1 (0.0%) | 7389 (0.1%) |  |
| 557 | S923 | 6 (0.1%) | 32449 (0.5%) |  |
| 558 | S924 | 1 (0.0%) | 11498 (0.2%) |  |
| 559 | S925 | 1 (0.0%) | 22385 (0.3%) |  |
| 560 | S927 | 4 (0.1%) | 818 (0.0%) |  |
| 561 | S929 | 7 (0.1%) | 4863 (0.1%) |  |
| 562 | S930 | 3 (0.0%) | 1612 (0.0%) |  |
| 563 | S931 | 0 (0.0%) | 2034 (0.0%) |  |
| 564 | S932 | 0 (0.0%) | 1036 (0.0%) |  |
| 565 | S933 | 0 (0.0%) | 664 (0.0%) |  |
| 566 | S934 | 2 (0.0%) | 189776 (2.9%) |  |
| 567 | S935 | 0 (0.0%) | 6373 (0.1%) |  |
| 568 | S936 | 0 (0.0%) | 24495 (0.4%) |  |
| 569 | S940 | 0 (0.0%) | 2 (0.0%) |  |
| 570 | S941 | 0 (0.0%) | 6 (0.0%) |  |
| 571 | S942 | 0 (0.0%) | 23 (0.0%) |  |
| 572 | S943 | 0 (0.0%) | 22 (0.0%) |  |
| 573 | S947 | 0 (0.0%) | 7 (0.0%) |  |
| 574 | S948 | 0 (0.0%) | 26 (0.0%) |  |
| 575 | S949 | 0 (0.0%) | 35 (0.0%) |  |
| 576 | S950 | 0 (0.0%) | 81 (0.0%) |  |
| 577 | S951 | 0 (0.0%) | 86 (0.0%) |  |
| 578 | S952 | 0 (0.0%) | 97 (0.0%) |  |
| 579 | S957 | 0 (0.0%) | 8 (0.0%) |  |
| 580 | S958 | 0 (0.0%) | 595 (0.0%) |  |
| 581 | S959 | 0 (0.0%) | 553 (0.0%) |  |
| 582 | S960 | 0 (0.0%) | 428 (0.0%) |  |
| 583 | S961 | 0 (0.0%) | 1187 (0.0%) |  |
| 584 | S962 | 0 (0.0%) | 263 (0.0%) |  |
| 585 | S967 | 0 (0.0%) | 163 (0.0%) |  |
| 586 | S968 | 0 (0.0%) | 808 (0.0%) |  |
| 587 | S969 | 0 (0.0%) | 1043 (0.0%) |  |
| 588 | S970 | 1 (0.0%) | 139 (0.0%) |  |
| 589 | S971 | 0 (0.0%) | 663 (0.0%) |  |
| 590 | S978 | 1 (0.0%) | 783 (0.0%) |  |
| 591 | S980 | 2 (0.0%) | 45 (0.0%) |  |
| 592 | S981 | 0 (0.0%) | 179 (0.0%) |  |
| 593 | S982 | 0 (0.0%) | 44 (0.0%) |  |
| 594 | S983 | 0 (0.0%) | 44 (0.0%) |  |
| 595 | S984 | 0 (0.0%) | 34 (0.0%) |  |
| 596 | S990 | 0 (0.0%) | 6 (0.0%) |  |
| 597 | S997 | 0 (0.0%) | 61 (0.0%) |  |
| 598 | S998 | 0 (0.0%) | 657 (0.0%) |  |
| 599 | S999 | 4 (0.1%) | 8808 (0.1%) |  |
| 600 | T000 | 0 (0.0%) | 4212 (0.1%) |  |
| 601 | T001 | 0 (0.0%) | 65 (0.0%) |  |
| 602 | T002 | 0 (0.0%) | 58 (0.0%) |  |
| 603 | T003 | 0 (0.0%) | 103 (0.0%) |  |
| 604 | T006 | 0 (0.0%) | 67 (0.0%) |  |
| 605 | T008 | 1 (0.0%) | 1853 (0.0%) |  |
| 606 | T009 | 561 (8.8%) | 186912 (2.9%) |  |
| 607 | T010 | 1 (0.0%) | 175 (0.0%) |  |
| 608 | T011 | 0 (0.0%) | 31 (0.0%) |  |
| 609 | T012 | 3 (0.0%) | 769 (0.0%) |  |
| 610 | T013 | 2 (0.0%) | 242 (0.0%) |  |
| 611 | T016 | 0 (0.0%) | 35 (0.0%) |  |
| 612 | T018 | 0 (0.0%) | 30 (0.0%) |  |
| 613 | T019 | 31 (0.5%) | 5740 (0.1%) |  |
| 614 | T020 | 0 (0.0%) | 5 (0.0%) |  |
| 615 | T021 | 5 (0.1%) | 75 (0.0%) |  |
| 616 | T022 | 0 (0.0%) | 6 (0.0%) |  |
| 617 | T023 | 1 (0.0%) | 15 (0.0%) |  |
| 618 | T024 | 0 (0.0%) | 6 (0.0%) |  |
| 619 | T025 | 5 (0.1%) | 19 (0.0%) |  |
| 620 | T026 | 2 (0.0%) | 5 (0.0%) |  |
| 621 | T027 | 5 (0.1%) | 13 (0.0%) |  |
| 622 | T028 | 2 (0.0%) | 157 (0.0%) |  |
| 623 | T029 | 62 (1.0%) | 237 (0.0%) |  |
| 624 | T030 | 0 (0.0%) | 605 (0.0%) |  |
| 625 | T031 | 0 (0.0%) | 2076 (0.0%) |  |
| 626 | T032 | 0 (0.0%) | 112 (0.0%) |  |
| 627 | T033 | 0 (0.0%) | 150 (0.0%) |  |
| 628 | T034 | 0 (0.0%) | 15 (0.0%) |  |
| 629 | T038 | 0 (0.0%) | 307 (0.0%) |  |
| 630 | T039 | 0 (0.0%) | 485 (0.0%) |  |
| 631 | T040 | 0 (0.0%) | 3 (0.0%) |  |
| 632 | T041 | 3 (0.0%) | 11 (0.0%) |  |
| 633 | T042 | 2 (0.0%) | 45 (0.0%) |  |
| 634 | T043 | 2 (0.0%) | 82 (0.0%) |  |
| 635 | T044 | 1 (0.0%) | 5 (0.0%) |  |
| 636 | T047 | 2 (0.0%) | 1 (0.0%) |  |
| 637 | T048 | 1 (0.0%) | 6 (0.0%) |  |
| 638 | T049 | 4 (0.1%) | 32 (0.0%) |  |
| 639 | T050 | 0 (0.0%) | 7 (0.0%) |  |
| 640 | T051 | 0 (0.0%) | 20 (0.0%) |  |
| 641 | T052 | 0 (0.0%) | 1 (0.0%) |  |
| 642 | T053 | 0 (0.0%) | 1 (0.0%) |  |
| 643 | T054 | 0 (0.0%) | 3 (0.0%) |  |
| 644 | T055 | 3 (0.0%) | 3 (0.0%) |  |
| 645 | T058 | 0 (0.0%) | 1 (0.0%) |  |
| 646 | T059 | 1 (0.0%) | 22 (0.0%) |  |
| 647 | T060 | 4 (0.1%) | 33 (0.0%) |  |
| 648 | T061 | 0 (0.0%) | 86 (0.0%) |  |
| 649 | T062 | 0 (0.0%) | 7 (0.0%) |  |
| 650 | T063 | 0 (0.0%) | 6 (0.0%) |  |
| 651 | T064 | 0 (0.0%) | 25 (0.0%) |  |
| 652 | T065 | 7 (0.1%) | 11 (0.0%) |  |
| 653 | T068 | 135 (2.1%) | 2543 (0.0%) |  |
| 654 | T070 | 226 (3.6%) | 2577 (0.0%) |  |
| 655 | T080 | 16 (0.3%) | 4416 (0.1%) |  |
| 656 | T081 | 0 (0.0%) | 15 (0.0%) |  |
| 657 | T090 | 4 (0.1%) | 1956 (0.0%) |  |
| 658 | T091 | 4 (0.1%) | 579 (0.0%) |  |
| 659 | T092 | 1 (0.0%) | 9822 (0.2%) |  |
| 660 | T093 | 13 (0.2%) | 2077 (0.0%) |  |
| 661 | T094 | 0 (0.0%) | 44 (0.0%) |  |
| 662 | T095 | 0 (0.0%) | 21 (0.0%) |  |
| 663 | T098 | 0 (0.0%) | 57 (0.0%) |  |
| 664 | T099 | 5 (0.1%) | 369 (0.0%) |  |
| 665 | T100 | 2 (0.0%) | 444 (0.0%) |  |
| 666 | T101 | 5 (0.1%) | 25 (0.0%) |  |
| 667 | T110 | 0 (0.0%) | 1977 (0.0%) |  |
| 668 | T111 | 1 (0.0%) | 1138 (0.0%) |  |
| 669 | T112 | 0 (0.0%) | 418 (0.0%) |  |
| 670 | T113 | 0 (0.0%) | 11 (0.0%) |  |
| 671 | T114 | 0 (0.0%) | 4 (0.0%) |  |
| 672 | T115 | 0 (0.0%) | 62 (0.0%) |  |
| 673 | T116 | 3 (0.0%) | 40 (0.0%) |  |
| 674 | T118 | 0 (0.0%) | 27 (0.0%) |  |
| 675 | T119 | 0 (0.0%) | 848 (0.0%) |  |
| 676 | T120 | 5 (0.1%) | 385 (0.0%) |  |
| 677 | T121 | 4 (0.1%) | 87 (0.0%) |  |
| 678 | T130 | 1 (0.0%) | 3449 (0.1%) |  |
| 679 | T131 | 4 (0.1%) | 3082 (0.0%) |  |
| 680 | T132 | 2 (0.0%) | 825 (0.0%) |  |
| 681 | T133 | 0 (0.0%) | 5 (0.0%) |  |
| 682 | T134 | 0 (0.0%) | 19 (0.0%) |  |
| 683 | T135 | 0 (0.0%) | 730 (0.0%) |  |
| 684 | T136 | 7 (0.1%) | 50 (0.0%) |  |
| 685 | T138 | 0 (0.0%) | 29 (0.0%) |  |
| 686 | T139 | 3 (0.0%) | 1386 (0.0%) |  |
| 687 | T140 | 77 (1.2%) | 170395 (2.6%) |  |
| 688 | T141 | 31 (0.5%) | 139264 (2.1%) |  |
| 689 | T142 | 9 (0.1%) | 1713 (0.0%) |  |
| 690 | T143 | 0 (0.0%) | 3334 (0.1%) |  |
| 691 | T144 | 0 (0.0%) | 83 (0.0%) |  |
| 692 | T145 | 6 (0.1%) | 277 (0.0%) |  |
| 693 | T146 | 1 (0.0%) | 7408 (0.1%) |  |
| 694 | T147 | 3 (0.0%) | 709 (0.0%) |  |
| 695 | T148 | 7 (0.1%) | 3670 (0.1%) |  |
| 696 | T149 | 16 (0.3%) | 968 (0.0%) |  |
| 697 | T150 | 0 (0.0%) | 15329 (0.2%) |  |
| 698 | T151 | 0 (0.0%) | 6010 (0.1%) |  |
| 699 | T158 | 0 (0.0%) | 1070 (0.0%) |  |
| 700 | T159 | 0 (0.0%) | 22457 (0.3%) |  |
| 701 | T160 | 0 (0.0%) | 19886 (0.3%) |  |
| 702 | T170 | 0 (0.0%) | 2480 (0.0%) |  |
| 703 | T171 | 0 (0.0%) | 23964 (0.4%) |  |
| 704 | T172 | 3 (0.0%) | 71783 (1.1%) |  |
| 705 | T173 | 0 (0.0%) | 5774 (0.1%) |  |
| 706 | T174 | 3 (0.0%) | 324 (0.0%) |  |
| 707 | T175 | 0 (0.0%) | 413 (0.0%) |  |
| 708 | T178 | 6 (0.1%) | 311 (0.0%) |  |
| 709 | T179 | 28 (0.4%) | 912 (0.0%) |  |
| 710 | T180 | 0 (0.0%) | 4454 (0.1%) |  |
| 711 | T181 | 0 (0.0%) | 15997 (0.2%) |  |
| 712 | T182 | 0 (0.0%) | 6289 (0.1%) |  |
| 713 | T183 | 0 (0.0%) | 646 (0.0%) |  |
| 714 | T184 | 0 (0.0%) | 352 (0.0%) |  |
| 715 | T185 | 0 (0.0%) | 519 (0.0%) |  |
| 716 | T188 | 0 (0.0%) | 537 (0.0%) |  |
| 717 | T189 | 3 (0.0%) | 24426 (0.4%) |  |
| 718 | T190 | 0 (0.0%) | 60 (0.0%) |  |
| 719 | T191 | 0 (0.0%) | 46 (0.0%) |  |
| 720 | T192 | 0 (0.0%) | 2302 (0.0%) |  |
| 721 | T193 | 0 (0.0%) | 35 (0.0%) |  |
| 722 | T198 | 0 (0.0%) | 77 (0.0%) |  |
| 723 | T199 | 0 (0.0%) | 124 (0.0%) |  |
| 724 | T200 | 3 (0.0%) | 3300 (0.1%) |  |
| 725 | T201 | 0 (0.0%) | 5046 (0.1%) |  |
| 726 | T202 | 2 (0.0%) | 13374 (0.2%) |  |
| 727 | T203 | 3 (0.0%) | 168 (0.0%) |  |
| 728 | T204 | 0 (0.0%) | 54 (0.0%) |  |
| 729 | T205 | 0 (0.0%) | 94 (0.0%) |  |
| 730 | T206 | 0 (0.0%) | 73 (0.0%) |  |
| 731 | T207 | 0 (0.0%) | 6 (0.0%) |  |
| 732 | T210 | 0 (0.0%) | 1694 (0.0%) |  |
| 733 | T211 | 0 (0.0%) | 2402 (0.0%) |  |
| 734 | T212 | 2 (0.0%) | 11099 (0.2%) |  |
| 735 | T213 | 2 (0.0%) | 232 (0.0%) |  |
| 736 | T214 | 0 (0.0%) | 20 (0.0%) |  |
| 737 | T215 | 0 (0.0%) | 19 (0.0%) |  |
| 738 | T216 | 0 (0.0%) | 19 (0.0%) |  |
| 739 | T220 | 0 (0.0%) | 2502 (0.0%) |  |
| 740 | T221 | 0 (0.0%) | 2298 (0.0%) |  |
| 741 | T222 | 0 (0.0%) | 14073 (0.2%) |  |
| 742 | T223 | 2 (0.0%) | 236 (0.0%) |  |
| 743 | T224 | 0 (0.0%) | 44 (0.0%) |  |
| 744 | T225 | 0 (0.0%) | 27 (0.0%) |  |
| 745 | T226 | 0 (0.0%) | 65 (0.0%) |  |
| 746 | T227 | 0 (0.0%) | 10 (0.0%) |  |
| 747 | T230 | 0 (0.0%) | 10877 (0.2%) |  |
| 748 | T231 | 0 (0.0%) | 10580 (0.2%) |  |
| 749 | T232 | 1 (0.0%) | 48319 (0.7%) |  |
| 750 | T233 | 1 (0.0%) | 689 (0.0%) |  |
| 751 | T234 | 0 (0.0%) | 87 (0.0%) |  |
| 752 | T235 | 0 (0.0%) | 83 (0.0%) |  |
| 753 | T236 | 0 (0.0%) | 189 (0.0%) |  |
| 754 | T237 | 0 (0.0%) | 16 (0.0%) |  |
| 755 | T240 | 0 (0.0%) | 3545 (0.1%) |  |
| 756 | T241 | 0 (0.0%) | 4031 (0.1%) |  |
| 757 | T242 | 1 (0.0%) | 25938 (0.4%) |  |
| 758 | T243 | 2 (0.0%) | 431 (0.0%) |  |
| 759 | T244 | 0 (0.0%) | 30 (0.0%) |  |
| 760 | T245 | 0 (0.0%) | 31 (0.0%) |  |
| 761 | T246 | 0 (0.0%) | 122 (0.0%) |  |
| 762 | T247 | 0 (0.0%) | 18 (0.0%) |  |
| 763 | T250 | 0 (0.0%) | 2669 (0.0%) |  |
| 764 | T251 | 0 (0.0%) | 2021 (0.0%) |  |
| 765 | T252 | 0 (0.0%) | 16335 (0.3%) |  |
| 766 | T253 | 0 (0.0%) | 279 (0.0%) |  |
| 767 | T254 | 0 (0.0%) | 39 (0.0%) |  |
| 768 | T255 | 0 (0.0%) | 14 (0.0%) |  |
| 769 | T256 | 0 (0.0%) | 30 (0.0%) |  |
| 770 | T257 | 0 (0.0%) | 17 (0.0%) |  |
| 771 | T260 | 0 (0.0%) | 665 (0.0%) |  |
| 772 | T261 | 0 (0.0%) | 1909 (0.0%) |  |
| 773 | T262 | 0 (0.0%) | 9 (0.0%) |  |
| 774 | T263 | 0 (0.0%) | 262 (0.0%) |  |
| 775 | T264 | 0 (0.0%) | 604 (0.0%) |  |
| 776 | T265 | 0 (0.0%) | 266 (0.0%) |  |
| 777 | T266 | 0 (0.0%) | 3059 (0.0%) |  |
| 778 | T267 | 0 (0.0%) | 33 (0.0%) |  |
| 779 | T268 | 1 (0.0%) | 137 (0.0%) |  |
| 780 | T269 | 0 (0.0%) | 409 (0.0%) |  |
| 781 | T270 | 1 (0.0%) | 94 (0.0%) |  |
| 782 | T271 | 2 (0.0%) | 53 (0.0%) |  |
| 783 | T272 | 2 (0.0%) | 176 (0.0%) |  |
| 784 | T273 | 6 (0.1%) | 677 (0.0%) |  |
| 785 | T274 | 0 (0.0%) | 10 (0.0%) |  |
| 786 | T277 | 0 (0.0%) | 3 (0.0%) |  |
| 787 | T280 | 0 (0.0%) | 219 (0.0%) |  |
| 788 | T281 | 2 (0.0%) | 29 (0.0%) |  |
| 789 | T282 | 0 (0.0%) | 8 (0.0%) |  |
| 790 | T283 | 0 (0.0%) | 46 (0.0%) |  |
| 791 | T284 | 0 (0.0%) | 16 (0.0%) |  |
| 792 | T285 | 1 (0.0%) | 68 (0.0%) |  |
| 793 | T286 | 0 (0.0%) | 103 (0.0%) |  |
| 794 | T287 | 1 (0.0%) | 109 (0.0%) |  |
| 795 | T289 | 0 (0.0%) | 5 (0.0%) |  |
| 796 | T290 | 2 (0.0%) | 804 (0.0%) |  |
| 797 | T291 | 0 (0.0%) | 164 (0.0%) |  |
| 798 | T292 | 1 (0.0%) | 656 (0.0%) |  |
| 799 | T293 | 2 (0.0%) | 105 (0.0%) |  |
| 800 | T294 | 0 (0.0%) | 6 (0.0%) |  |
| 801 | T295 | 0 (0.0%) | 2 (0.0%) |  |
| 802 | T296 | 0 (0.0%) | 3 (0.0%) |  |
| 803 | T300 | 9 (0.1%) | 37967 (0.6%) |  |
| 804 | T301 | 0 (0.0%) | 3956 (0.1%) |  |
| 805 | T302 | 2 (0.0%) | 18995 (0.3%) |  |
| 806 | T303 | 5 (0.1%) | 181 (0.0%) |  |
| 807 | T304 | 0 (0.0%) | 1454 (0.0%) |  |
| 808 | T305 | 0 (0.0%) | 5 (0.0%) |  |
| 809 | T306 | 0 (0.0%) | 10 (0.0%) |  |
| 810 | T307 | 0 (0.0%) | 3 (0.0%) |  |
| 811 | T310 | 0 (0.0%) | 5281 (0.1%) |  |
| 812 | T311 | 0 (0.0%) | 406 (0.0%) |  |
| 813 | T312 | 0 (0.0%) | 123 (0.0%) |  |
| 814 | T313 | 0 (0.0%) | 127 (0.0%) |  |
| 815 | T314 | 0 (0.0%) | 61 (0.0%) |  |
| 816 | T315 | 3 (0.0%) | 41 (0.0%) |  |
| 817 | T316 | 0 (0.0%) | 47 (0.0%) |  |
| 818 | T317 | 1 (0.0%) | 28 (0.0%) |  |
| 819 | T318 | 1 (0.0%) | 29 (0.0%) |  |
| 820 | T319 | 3 (0.0%) | 31 (0.0%) |  |
| 821 | T320 | 0 (0.0%) | 6 (0.0%) |  |
| 822 | T322 | 0 (0.0%) | 2 (0.0%) |  |
| 823 | T326 | 0 (0.0%) | 1 (0.0%) |  |
| 824 | T790 | 1 (0.0%) | 6 (0.0%) |  |
| 825 | T791 | 1 (0.0%) | 22 (0.0%) |  |
| 826 | T792 | 4 (0.1%) | 41 (0.0%) |  |
| 827 | T793 | 0 (0.0%) | 906 (0.0%) |  |
| 828 | T794 | 556 (8.8%) | 529 (0.0%) |  |
| 829 | T795 | 1 (0.0%) | 3 (0.0%) |  |
| 830 | T796 | 2 (0.0%) | 1047 (0.0%) |  |
| 831 | T797 | 74 (1.2%) | 861 (0.0%) |  |
| 832 | T798 | 0 (0.0%) | 29 (0.0%) |  |
| 833 | T799 | 1 (0.0%) | 15 (0.0%) |  |
| 834 | T900 | 0 (0.0%) | 69 (0.0%) |  |
| 835 | T901 | 0 (0.0%) | 50 (0.0%) |  |
| 836 | T902 | 0 (0.0%) | 130 (0.0%) |  |
| 837 | T903 | 0 (0.0%) | 1 (0.0%) |  |
| 838 | T904 | 0 (0.0%) | 32 (0.0%) |  |
| 839 | T905 | 2 (0.0%) | 67 (0.0%) |  |
| 840 | T908 | 0 (0.0%) | 5 (0.0%) |  |
| 841 | T909 | 0 (0.0%) | 34 (0.0%) |  |
| 842 | T910 | 0 (0.0%) | 41 (0.0%) |  |
| 843 | T911 | 0 (0.0%) | 222 (0.0%) |  |
| 844 | T912 | 0 (0.0%) | 78 (0.0%) |  |
| 845 | T913 | 0 (0.0%) | 66 (0.0%) |  |
| 846 | T914 | 0 (0.0%) | 2 (0.0%) |  |
| 847 | T915 | 0 (0.0%) | 2 (0.0%) |  |
| 848 | T920 | 0 (0.0%) | 8 (0.0%) |  |
| 849 | T921 | 0 (0.0%) | 15 (0.0%) |  |
| 850 | T922 | 0 (0.0%) | 21 (0.0%) |  |
| 851 | T923 | 0 (0.0%) | 50 (0.0%) |  |
| 852 | T925 | 0 (0.0%) | 13 (0.0%) |  |
| 853 | T926 | 0 (0.0%) | 6 (0.0%) |  |
| 854 | T929 | 0 (0.0%) | 1 (0.0%) |  |
| 855 | T930 | 0 (0.0%) | 33 (0.0%) |  |
| 856 | T931 | 0 (0.0%) | 23 (0.0%) |  |
| 857 | T932 | 0 (0.0%) | 17 (0.0%) |  |
| 858 | T933 | 0 (0.0%) | 74 (0.0%) |  |
| 859 | T934 | 0 (0.0%) | 6 (0.0%) |  |
| 860 | T935 | 0 (0.0%) | 15 (0.0%) |  |
| 861 | T936 | 0 (0.0%) | 7 (0.0%) |  |
| 862 | T938 | 0 (0.0%) | 1 (0.0%) |  |
| 863 | T939 | 0 (0.0%) | 3 (0.0%) |  |
| 864 | T940 | 0 (0.0%) | 1 (0.0%) |  |
| 865 | T941 | 0 (0.0%) | 5 (0.0%) |  |
| 866 | T950 | 0 (0.0%) | 28 (0.0%) |  |
| 867 | T951 | 0 (0.0%) | 7 (0.0%) |  |
| 868 | T952 | 0 (0.0%) | 80 (0.0%) |  |
| 869 | T953 | 0 (0.0%) | 29 (0.0%) |  |
| 870 | T954 | 0 (0.0%) | 2 (0.0%) |  |
| 871 | T958 | 0 (0.0%) | 9 (0.0%) |  |
| 872 | T959 | 0 (0.0%) | 33 (0.0%) |  |
| 873 | T960 | 0 (0.0%) | 80 (0.0%) |  |
| 874 | T970 | 0 (0.0%) | 87 (0.0%) |  |
| 875 | T980 | 0 (0.0%) | 48 (0.0%) |  |
| 876 | T981 | 0 (0.0%) | 14 (0.0%) |  |
| 877 | T982 | 0 (0.0%) | 1 (0.0%) |  |
| 878 | T983 | 0 (0.0%) | 3 (0.0%) |  |
| ICD-10, international classification of disease 10th edition | | | | |

| **Table S4. Ranked normalized feature importance values from AdaBoost** | | |
| --- | --- | --- |
| **Rank** | **Name** | **Value** |
| 1 | Age | 0.01225 |
| 2 | Systolic blood pressure | 0.0115 |
| 3 | Unresponsive (coma) | 0.0065 |
| 4 | Pulse rate per minute | 0.00625 |
| 5 | Oxygen saturation | 0.00575 |
| 6 | Initial KTAS level 5 | 0.00325 |
| 7 | S224 | 0.003 |
| 8 | Respiratory rate per minute | 0.003 |
| 9 | Painful response (stupor, semi coma) | 0.0025 |
| 10 | S328 | 0.0025 |
| 11 | S320 | 0.0025 |
| 12 | Initial KTAS level 4 | 0.0025 |
| 13 | Initial KTAS level 2 | 0.0025 |
| 14 | Initial KTAS level 1 | 0.0025 |
| 15 | Alert | 0.00225 |
| 16 | Emergent symptoms - emergency | 0.00225 |
| 17 | S368 | 0.002 |
| 18 | S271 | 0.002 |
| 19 | S020 | 0.00175 |
| 20 | Diastolic blood pressure | 0.00175 |
| 21 | S021 | 0.00175 |
| 22 | Injury mechanism - OTHERS | 0.00175 |
| 23 | S335 | 0.0015 |
| 24 | S824 | 0.00125 |
| 25 | Unknown Response | 0.00125 |
| 26 | S361 | 0.00125 |
| 27 | Injury mechanism - TRAFFIC OTHERS | 0.00125 |
| 28 | S134 | 0.001 |
| 29 | S321 | 0.001 |
| 30 | S800 | 0.001 |
| 31 | Injury mechanism - slip down | 0.001 |
| 32 | Emergent symptoms – non-emergency | 0.00075 |
| 33 | S202 | 0.0005 |
| 34 | S028 | 0.0005 |
| 35 | S129 | 0.0005 |
| 36 | S822 | 0.0005 |
| 37 | Body temperature | 0.0005 |
| 38 | S024 | 0.0005 |
| 39 | S272 | 0.0005 |
| 40 | Injury mechanism - fire, flames or heat | 0.0005 |
| 41 | S611 | 0.0005 |
| 42 | S423 | 0.0005 |
| 43 | Intentionality - unspecified | 0.00025 |
| 44 | Intentionality - self-harm, suicide | 0.00025 |
| 45 | Injury mechanism - traffic accident-motorcycle | 0.00025 |
| 46 | S500 | 0.00025 |
| 47 | T070 | 0.00025 |
| 48 | S610 | 0.00025 |
| 49 | T029 | 0.00025 |
| KTAS, Korean triage and acuity scale; | | |

| **Table S5. Survival risk ratio of ICD-10 for calculation of ICISS** | | | | |  |  |  |  |
| --- | --- | --- | --- | --- | --- | --- | --- | --- |
| **Inclusive SRR** | | | | |  |  |  |  |
| **Code** | **Name** | **Total**  **(N)** | **Survivor**  **(N)** | **Inclusive**  **SRR (%)** |  |  |  |  |
| S00 | Superficial injury of head | 600421 | 599805 | 99.90 |  |  |  |  |
| S001 | Contusion of eyelid and periocular area | 31277 | 31254 | 99.93 |  |  |  |  |
| S002 | Other superficial injuries of eyelid and periocular area | 10130 | 10119 | 99.89 |  |  |  |  |
| S003 | Superficial injury of nose | 34883 | 34846 | 99.89 |  |  |  |  |
| S004 | Superficial injury of ear | 9833 | 9821 | 99.88 |  |  |  |  |
| S005 | Superficial injury of lip and oral cavity | 22355 | 22334 | 99.91 |  |  |  |  |
| S007 | Multiple superficial injuries of head | 2553 | 2548 | 99.80 |  |  |  |  |
| S008 | Superficial injury of other parts of head | 281721 | 281425 | 99.89 |  |  |  |  |
| S009 | Superficial injury of head, part unspecified | 93812 | 93712 | 99.89 |  |  |  |  |
| S01 | Open wound of head | 1014149 | 1013167 | 99.90 |  |  |  |  |
| S010 | Open wound of scalp | 254763 | 254514 | 99.90 |  |  |  |  |
| S011 | Open wound of eyelid and periocular area | 133649 | 133511 | 99.90 |  |  |  |  |
| S012 | Open wound of nose | 21805 | 21791 | 99.94 |  |  |  |  |
| S013 | Open wound of ear | 26237 | 26213 | 99.91 |  |  |  |  |
| S014 | Open wound of cheek and temporomandibular area | 44748 | 44706 | 99.91 |  |  |  |  |
| S015 | Open wound of lip | 122976 | 122857 | 99.90 |  |  |  |  |
| S017 | Multiple open wounds of head | 4114 | 4106 | 99.81 |  |  |  |  |
| S018 | Open wound of other parts of head | 366973 | 366615 | 99.90 |  |  |  |  |
| S019 | Open wound of head, part unspecified | 38884 | 38854 | 99.92 |  |  |  |  |
| S02 | Fracture of skull and facial bones | 229759 | 229554 | 99.91 |  |  |  |  |
| S020 | Fracture of vault of skull | 11550 | 11538 | 99.90 |  |  |  |  |
| S021 | Fracture of base of skull | 12917 | 12900 | 99.87 |  |  |  |  |
| S022 | Fracture of nasal bones | 77740 | 77669 | 99.91 |  |  |  |  |
| S023 | Fracture of orbital floor | 23696 | 23671 | 99.89 |  |  |  |  |
| S024 | Fracture of malar and maxillary bones | 26473 | 26453 | 99.92 |  |  |  |  |
| S025 | Fracture of tooth | 22037 | 22026 | 99.95 |  |  |  |  |
| S026 | Fracture of mandible | 11561 | 11548 | 99.89 |  |  |  |  |
| S027 | Multiple fractures involving skull and facial bones | 1363 | 1362 | 99.93 |  |  |  |  |
| S028 | Fractures of other skull and facial bones | 29271 | 29247 | 99.92 |  |  |  |  |
| S029 | Fracture of skull and facial bones, part unspecified | 13151 | 13140 | 99.92 |  |  |  |  |
| S03 | Dislocation, sprain and strain of joints and ligaments of head | 28989 | 28968 | 99.93 |  |  |  |  |
| S030 | Dislocation of jaw | 7675 | 7669 | 99.92 |  |  |  |  |
| S031 | Dislocation of septal cartilage of nose | 14 | 14 | 100.00 |  |  |  |  |
| S032 | Dislocation of tooth | 19882 | 19869 | 99.93 |  |  |  |  |
| S033 | Dislocation of other and unspecified parts of head | 4 | 4 | 100.00 |  |  |  |  |
| S034 | Sprain and strain of jaw | 1097 | 1096 | 99.91 |  |  |  |  |
| S035 | Sprain and strain of joints and ligaments of other and unspecified parts of head | 317 | 316 | 99.68 |  |  |  |  |
| S04 | Injury of cranial nerve | 660 | 660 | 100.00 |  |  |  |  |
| S040 | Injury of optic nerve and pathways | 316 | 316 | 100.00 |  |  |  |  |
| S041 | Injury of oculomotor nerve | 37 | 37 | 100.00 |  |  |  |  |
| S042 | Injury of trochlear nerve | 19 | 19 | 100.00 |  |  |  |  |
| S043 | Injury of trigeminal nerve | 36 | 36 | 100.00 |  |  |  |  |
| S044 | Injury of abducent nerve | 9 | 9 | 100.00 |  |  |  |  |
| S045 | Injury of facial nerve | 204 | 204 | 100.00 |  |  |  |  |
| S046 | Injury of acoustic nerve | 27 | 27 | 100.00 |  |  |  |  |
| S047 | Injury of accessory nerve | 0 | 0 | 0.00 |  |  |  |  |
| S048 | Injury of other cranial nerves | 4 | 4 | 100.00 |  |  |  |  |
| S049 | Injury of unspecified cranial nerve | 8 | 8 | 100.00 |  |  |  |  |
| S05 | Injury of eye and orbit | 102454 | 102365 | 99.91 |  |  |  |  |
| S050 | Injury of conjunctiva and corneal abrasion without mention of foreign body | 51321 | 51276 | 99.91 |  |  |  |  |
| S051 | Contusion of eyeball and orbital tissues | 31111 | 31080 | 99.90 |  |  |  |  |
| S052 | Ocular laceration and rupture with prolapse or loss of intraocular tissue | 489 | 489 | 100.00 |  |  |  |  |
| S053 | Ocular laceration without prolapse or loss of intraocular tissue | 2428 | 2427 | 99.96 |  |  |  |  |
| S054 | Penetrating wound of orbit with or without foreign body | 157 | 157 | 100.00 |  |  |  |  |
| S055 | Penetrating wound of eyeball with foreign body | 683 | 682 | 99.85 |  |  |  |  |
| S056 | Penetrating wound of eyeball without foreign body | 507 | 507 | 100.00 |  |  |  |  |
| S057 | Avulsion of eye | 64 | 64 | 100.00 |  |  |  |  |
| S058 | Other injuries of eye and orbit | 7514 | 7508 | 99.92 |  |  |  |  |
| S059 | Injury of eye and orbit, unspecified | 8180 | 8175 | 99.94 |  |  |  |  |
| S06 | Intracranial injury | 674263 | 673616 | 99.90 |  |  |  |  |
| S060 | Concussion | 553852 | 553315 | 99.90 |  |  |  |  |
| S061 | Traumatic cerebral oedema | 218 | 218 | 100.00 |  |  |  |  |
| S062 | Diffuse brain injury | 17093 | 17080 | 99.92 |  |  |  |  |
| S063 | Focal brain injury | 6815 | 6810 | 99.93 |  |  |  |  |
| S064 | Epidural haemorrhage | 11452 | 11445 | 99.94 |  |  |  |  |
| S065 | Traumatic subdural haemorrhage | 54312 | 54255 | 99.90 |  |  |  |  |
| S066 | Traumatic subarachnoid haemorrhage | 21378 | 21359 | 99.91 |  |  |  |  |
| S067 | Intracranial injury with prolonged coma | 30 | 30 | 100.00 |  |  |  |  |
| S068 | Other intracranial injuries | 7801 | 7794 | 99.91 |  |  |  |  |
| S069 | Intracranial injury, unspecified | 1312 | 1310 | 99.85 |  |  |  |  |
| S07 | Crushing injury of head | 589 | 588 | 99.83 |  |  |  |  |
| S070 | Crushing injury of face | 456 | 456 | 100.00 |  |  |  |  |
| S071 | Crushing injury of skull | 33 | 33 | 100.00 |  |  |  |  |
| S078 | Crushing injury of other parts of head | 56 | 55 | 98.21 |  |  |  |  |
| S079 | Crushing injury of head, part unspecified | 44 | 44 | 100.00 |  |  |  |  |
| S08 | Traumatic amputation of part of head | 979 | 978 | 99.90 |  |  |  |  |
| S080 | Avulsion of scalp | 886 | 886 | 100.00 |  |  |  |  |
| S081 | Traumatic amputation of ear | 77 | 77 | 100.00 |  |  |  |  |
| S088 | Traumatic amputation of other parts of head | 8 | 8 | 100.00 |  |  |  |  |
| S089 | Traumatic amputation of unspecified part of head | 8 | 7 | 87.50 |  |  |  |  |
| S09 | Other and unspecified injuries of head | 56618 | 56564 | 99.90 |  |  |  |  |
| S090 | Injury of blood vessels of head, NEC | 38 | 38 | 100.00 |  |  |  |  |
| S091 | Injury of muscle and tendon of head | 5124 | 5119 | 99.90 |  |  |  |  |
| S092 | Traumatic rupture of ear drum | 3070 | 3068 | 99.93 |  |  |  |  |
| S097 | Multiple injuries of head | 59 | 59 | 100.00 |  |  |  |  |
| S098 | Other specified injuries of head | 2137 | 2134 | 99.86 |  |  |  |  |
| S099 | Unspecified injury of head | 46190 | 46146 | 99.90 |  |  |  |  |
| S10 | Superficial injury of neck | 30028 | 30009 | 99.94 |  |  |  |  |
| S100 | Contusion of throat | 783 | 783 | 100.00 |  |  |  |  |
| S101 | Other and unspecified superficial injuries of throat | 2305 | 2303 | 99.91 |  |  |  |  |
| S107 | Multiple superficial injuries of neck | 169 | 169 | 100.00 |  |  |  |  |
| S108 | Superficial injury of other parts of neck | 9866 | 9861 | 99.95 |  |  |  |  |
| S109 | Superficial injury of neck, part unspecified | 16905 | 16893 | 99.93 |  |  |  |  |
| S11 | Open wound of neck | 4238 | 4233 | 99.88 |  |  |  |  |
| S111 | Open wound involving thyroid gland | 7 | 7 | 100.00 |  |  |  |  |
| S112 | Open wound involving pharynx and cervical esophagus | 75 | 75 | 100.00 |  |  |  |  |
| S117 | Multiple open wounds of neck | 226 | 226 | 100.00 |  |  |  |  |
| S118 | Open wound of other parts of neck | 1188 | 1187 | 99.92 |  |  |  |  |
| S119 | Open wound of neck, part unspecified | 2664 | 2660 | 99.85 |  |  |  |  |
| S12 | Fracture of neck | 12592 | 12585 | 99.94 |  |  |  |  |
| S120 | Fracture of first cervical vertebra | 627 | 626 | 99.84 |  |  |  |  |
| S121 | Fracture of second cervical vertebra | 1503 | 1501 | 99.87 |  |  |  |  |
| S122 | Fracture of other specified cervical vertebra | 2746 | 2745 | 99.96 |  |  |  |  |
| S127 | Multiple fractures of cervical spine | 1090 | 1090 | 100.00 |  |  |  |  |
| S128 | Fracture of other parts of neck | 261 | 261 | 100.00 |  |  |  |  |
| S129 | Fracture of neck, part unspecified | 6365 | 6362 | 99.95 |  |  |  |  |
| S13 | Dislocation, sprain and strain of joints and ligaments at neck level | 389619 | 389233 | 99.90 |  |  |  |  |
| S130 | Traumatic rupture of cervical intervertebral disc | 381 | 381 | 100.00 |  |  |  |  |
| S131 | Dislocation of cervical vertebra | 956 | 955 | 99.90 |  |  |  |  |
| S132 | Dislocation of other and unspecified parts of neck | 11 | 11 | 100.00 |  |  |  |  |
| S133 | Multiple dislocations of neck | 6 | 6 | 100.00 |  |  |  |  |
| S134 | Sprain and strain of cervical spine | 361359 | 361001 | 99.90 |  |  |  |  |
| S135 | Sprain and strain of thyroid region | 28 | 28 | 100.00 |  |  |  |  |
| S136 | Sprain and strain of joints and ligaments of other and unspecified parts of neck | 26878 | 26851 | 99.90 |  |  |  |  |
| S14 | Injury of nerves and spinal cord at neck level | 5170 | 5169 | 99.98 |  |  |  |  |
| S140 | Concussion and oedema of cervical spinal cord | 564 | 564 | 100.00 |  |  |  |  |
| S141 | Other and unspecified injuries of cervical spinal cord | 4103 | 4103 | 100.00 |  |  |  |  |
| S142 | Injury of nerve root of cervical spine | 215 | 215 | 100.00 |  |  |  |  |
| S143 | Injury of brachial plexus | 202 | 201 | 99.50 |  |  |  |  |
| S144 | Injury of peripheral nerves of neck | 5 | 5 | 100.00 |  |  |  |  |
| S145 | Injury of cervical sympathetic nerves | 0 | 0 | 0.00 |  |  |  |  |
| S146 | Injury of other and unspecified nerves of neck | 81 | 81 | 100.00 |  |  |  |  |
| S15 | Injury of blood vessels at neck level | 352 | 352 | 100.00 |  |  |  |  |
| S150 | Injury of carotid artery | 33 | 33 | 100.00 |  |  |  |  |
| S151 | Injury of vertebral artery | 27 | 27 | 100.00 |  |  |  |  |
| S152 | Injury of external jugular vein | 16 | 16 | 100.00 |  |  |  |  |
| S153 | Injury of internal jugular vein | 17 | 17 | 100.00 |  |  |  |  |
| S157 | Injury of multiple blood vessels at neck level | 11 | 11 | 100.00 |  |  |  |  |
| S158 | Injury of other blood vessels at neck level | 117 | 117 | 100.00 |  |  |  |  |
| S159 | Injury of unspecified blood vessels at neck level | 131 | 131 | 100.00 |  |  |  |  |
| S16 | Injury of muscle and tendon at neck level | 114 | 114 | 100.00 |  |  |  |  |
| S17 | Crushing injury of neck | 40 | 40 | 100.00 |  |  |  |  |
| S170 | Crushing injury of larynx and trachea | 20 | 20 | 100.00 |  |  |  |  |
| S178 | Crushing injury of other parts of neck | 3 | 3 | 100.00 |  |  |  |  |
| S179 | Crushing injury of neck, part unspecified | 17 | 17 | 100.00 |  |  |  |  |
| S18 | Traumatic amputation at neck level | 3 | 3 | 100.00 |  |  |  |  |
| S19 | Other and unspecified injuries of neck | 1303 | 1303 | 100.00 |  |  |  |  |
| S197 | Multiple injuries of neck | 15 | 15 | 100.00 |  |  |  |  |
| S198 | Other specified injuries of neck | 281 | 281 | 100.00 |  |  |  |  |
| S199 | Unspecified injury of neck | 1003 | 1003 | 100.00 |  |  |  |  |
| S20 | Superficial injury of thorax | 196927 | 196732 | 99.90 |  |  |  |  |
| S200 | Contusion of breast | 171 | 171 | 100.00 |  |  |  |  |
| S201 | Other and unspecified superficial injuries of breast | 84 | 84 | 100.00 |  |  |  |  |
| S202 | Contusion of thorax | 188218 | 188034 | 99.90 |  |  |  |  |
| S203 | Other superficial injuries of front wall of thorax | 1309 | 1308 | 99.92 |  |  |  |  |
| S204 | Other superficial injuries of back wall of thorax | 1043 | 1042 | 99.90 |  |  |  |  |
| S207 | Multiple superficial injuries of thorax | 108 | 108 | 100.00 |  |  |  |  |
| S208 | Superficial injury of other and unspecified parts of thorax | 5994 | 5985 | 99.85 |  |  |  |  |
| S21 | Open wound of thorax | 4059 | 4058 | 99.98 |  |  |  |  |
| S210 | Open wound of breast | 108 | 108 | 100.00 |  |  |  |  |
| S211 | Open wound of front wall of thorax | 1246 | 1246 | 100.00 |  |  |  |  |
| S212 | Open wound of back wall of thorax | 1316 | 1316 | 100.00 |  |  |  |  |
| S217 | Multiple open wounds of thoracic wall | 48 | 48 | 100.00 |  |  |  |  |
| S218 | Open wound of other parts of thorax | 255 | 255 | 100.00 |  |  |  |  |
| S219 | Open wound of thorax, part unspecified | 1086 | 1085 | 99.91 |  |  |  |  |
| S22 | Fracture of rib(s), sternum and thoracic spine | 164202 | 164046 | 99.90 |  |  |  |  |
| S220 | Fracture of thoracic vertebra | 29415 | 29375 | 99.86 |  |  |  |  |
| S221 | Multiple fractures of thoracic spine | 1644 | 1643 | 99.94 |  |  |  |  |
| S222 | Fracture of sternum | 10420 | 10412 | 99.92 |  |  |  |  |
| S223 | Fracture of rib | 49076 | 49033 | 99.91 |  |  |  |  |
| S224 | Multiple fracture of ribs | 72497 | 72434 | 99.91 |  |  |  |  |
| S225 | Flail chest | 481 | 480 | 99.79 |  |  |  |  |
| S228 | Fracture of other parts of bony thorax | 396 | 396 | 100.00 |  |  |  |  |
| S229 | Fracture of bony thorax, part unspecified | 273 | 273 | 100.00 |  |  |  |  |
| S23 | Dislocation, sprain and strain of joints and ligaments of thorax | 29206 | 29182 | 99.92 |  |  |  |  |
| S230 | Traumatic rupture of thoracic intervertebral disc | 22 | 22 | 100.00 |  |  |  |  |
| S231 | Dislocation of thoracic vertebra | 87 | 87 | 100.00 |  |  |  |  |
| S232 | Dislocation of other and unspecified parts of thorax | 21 | 21 | 100.00 |  |  |  |  |
| S233 | Sprain and strain of thoracic spine | 15952 | 15941 | 99.93 |  |  |  |  |
| S234 | Sprain and strain of ribs and sternum | 9395 | 9387 | 99.91 |  |  |  |  |
| S235 | Sprain and strain of other and unspecified parts of thorax | 3729 | 3724 | 99.87 |  |  |  |  |
| S24 | Injury of nerves and spinal cord at thorax level | 224 | 224 | 100.00 |  |  |  |  |
| S240 | Concussion and oedema of thoracic spinal cord | 20 | 20 | 100.00 |  |  |  |  |
| S241 | Other and unspecified injuries of thoracic spinal cord | 156 | 156 | 100.00 |  |  |  |  |
| S242 | Injury of nerve root of thoracic spine | 33 | 33 | 100.00 |  |  |  |  |
| S243 | Injury of peripheral nerves of thorax | 0 | 0 | 0.00 |  |  |  |  |
| S244 | Injury of thoracic sympathetic nerves | 7 | 7 | 100.00 |  |  |  |  |
| S245 | Injury of other nerves of thorax | 1 | 1 | 100.00 |  |  |  |  |
| S246 | Injury of unspecified nerve of thorax | 7 | 7 | 100.00 |  |  |  |  |
| S25 | Injury of blood vessels of thorax | 568 | 568 | 100.00 |  |  |  |  |
| S250 | Injury of thoracic aorta | 349 | 349 | 100.00 |  |  |  |  |
| S251 | Injury of innominate or subclavian artery | 21 | 21 | 100.00 |  |  |  |  |
| S252 | Injury of superior vena cava | 6 | 6 | 100.00 |  |  |  |  |
| S253 | Injury of innominate or subclavian vein | 10 | 10 | 100.00 |  |  |  |  |
| S254 | Injury of pulmonary blood vessels | 32 | 32 | 100.00 |  |  |  |  |
| S255 | Injury of intercostal blood vessels | 34 | 34 | 100.00 |  |  |  |  |
| S257 | Injury of multiple blood vessels of thorax | 2 | 2 | 100.00 |  |  |  |  |
| S258 | Injury of other blood vessels of thorax | 69 | 69 | 100.00 |  |  |  |  |
| S259 | Injury of unspecified blood vessel of thorax | 45 | 45 | 100.00 |  |  |  |  |
| S26 | Injury of heart | 965 | 965 | 100.00 |  |  |  |  |
| S260 | Injury of heart with hemopericardium | 216 | 216 | 100.00 |  |  |  |  |
| S268 | Other injuries of heart | 392 | 392 | 100.00 |  |  |  |  |
| S269 | Injury of heart, unspecified | 357 | 357 | 100.00 |  |  |  |  |
| S27 | injury of other and unspecified intrathoracic organs | 28938 | 28914 | 99.92 |  |  |  |  |
| S270 | Traumatic pneumothorax | 7764 | 7761 | 99.96 |  |  |  |  |
| S271 | Traumatic hemothorax | 7434 | 7428 | 99.92 |  |  |  |  |
| S272 | Traumatic hemopneumothorax | 6532 | 6525 | 99.89 |  |  |  |  |
| S273 | Other injuries of lung | 6271 | 6263 | 99.87 |  |  |  |  |
| S274 | Injury of bronchus | 27 | 27 | 100.00 |  |  |  |  |
| S275 | Injury of thoracic trachea | 183 | 183 | 100.00 |  |  |  |  |
| S276 | Injury of pleura | 48 | 48 | 100.00 |  |  |  |  |
| S277 | Multiple injuries of intrathoracic organs | 40 | 40 | 100.00 |  |  |  |  |
| S278 | Injury of other specified intrathoracic organs | 558 | 558 | 100.00 |  |  |  |  |
| S279 | Injury of unspecified intrathoracic organ | 81 | 81 | 100.00 |  |  |  |  |
| S28 | Crushing injury of thorax and traumatic amputation of part of thorax | 50 | 50 | 100.00 |  |  |  |  |
| S280 | Crushed chest | 32 | 32 | 100.00 |  |  |  |  |
| S281 | Traumatic amputation of part of thorax | 18 | 18 | 100.00 |  |  |  |  |
| S29 | Other and unspecified injuries of thorax | 1165 | 1165 | 100.00 |  |  |  |  |
| S290 | Injury of muscle and tendon at thorax level | 54 | 54 | 100.00 |  |  |  |  |
| S297 | Multiple injuries of thorax | 24 | 24 | 100.00 |  |  |  |  |
| S298 | Other specified injuries of thorax | 201 | 201 | 100.00 |  |  |  |  |
| S299 | Unspecified injury of thorax | 886 | 886 | 100.00 |  |  |  |  |
| S30 | Superficial injury of abdomen, lower back and pelvis | 163455 | 163303 | 99.91 |  |  |  |  |
| S300 | Contusion of lower back and pelvis | 94397 | 94295 | 99.89 |  |  |  |  |
| S301 | Contusion of abdominal wall | 50856 | 50816 | 99.92 |  |  |  |  |
| S302 | Contusion of external genital organs | 6114 | 6111 | 99.95 |  |  |  |  |
| S307 | Multiple superficial injuries of abdomen, lower back and pelvis | 247 | 247 | 100.00 |  |  |  |  |
| S308 | Other superficial injuries of abdomen, lower back and pelvis | 10095 | 10090 | 99.95 |  |  |  |  |
| S309 | Superficial injury of abdomen, lower back and pelvis, part unspecified | 1746 | 1744 | 99.89 |  |  |  |  |
| S31 | Open wound of abdomen, lower back and pelvis | 14299 | 14290 | 99.94 |  |  |  |  |
| S32 | Fracture of lumbar spine and pelvis | 107492 | 107384 | 99.90 |  |  |  |  |
| S320 | Fracture of lumbar vertebra | 60837 | 60770 | 99.89 |  |  |  |  |
| S321 | Fracture of sacrum | 5208 | 5204 | 99.92 |  |  |  |  |
| S322 | Fracture of coccyx | 6349 | 6345 | 99.94 |  |  |  |  |
| S323 | Fracture of ilium | 2287 | 2283 | 99.83 |  |  |  |  |
| S324 | Fracture of acetabulum | 4856 | 4850 | 99.88 |  |  |  |  |
| S325 | Fracture of pubis | 7092 | 7083 | 99.87 |  |  |  |  |
| S327 | Multiple fractures of lumbar spine and pelvis | 2418 | 2416 | 99.92 |  |  |  |  |
| S328 | Fracture of other and unspecified parts of lumbar spine and pelvis | 18445 | 18433 | 99.93 |  |  |  |  |
| S33 | Dislocation, sprain and strain of joints and ligaments of lumbar spine and pelvis | 288733 | 288421 | 99.89 |  |  |  |  |
| S330 | Traumatic rupture of lumbar intervertebral disc | 266 | 266 | 100.00 |  |  |  |  |
| S331 | Dislocation of lumbar vertebra | 209 | 209 | 100.00 |  |  |  |  |
| S332 | Dislocation of sacroiliac and sacrococcygeal joint | 103 | 103 | 100.00 |  |  |  |  |
| S333 | Dislocation of other and unspecified parts of lumbar spine and pelvis | 142 | 142 | 100.00 |  |  |  |  |
| S334 | Traumatic rupture of symphysis pubis | 97 | 97 | 100.00 |  |  |  |  |
| S335 | Sprain and strain of lumbar spine | 271108 | 270809 | 99.89 |  |  |  |  |
| S336 | Sprain and strain of sacroiliac joint | 424 | 423 | 99.76 |  |  |  |  |
| S337 | Sprain and strain of other and unspecified parts of lumbar spine and pelvis | 16384 | 16372 | 99.93 |  |  |  |  |
| S34 | Injury of nerves and lumbar spinal cord at abdomen, lower back and pelvis level | 626 | 625 | 99.84 |  |  |  |  |
| S340 | Concussion and oedema of lumbar spinal cord | 55 | 55 | 100.00 |  |  |  |  |
| S341 | Other injury of lumbar spinal cord | 444 | 444 | 100.00 |  |  |  |  |
| S342 | Injury of nerve root of lumbar and sacral spine | 58 | 58 | 100.00 |  |  |  |  |
| S343 | Injury of cauda equina | 16 | 16 | 100.00 |  |  |  |  |
| S344 | Injury of lumbosacral plexus | 10 | 10 | 100.00 |  |  |  |  |
| S345 | Injury of lumbar, sacral and pelvic sympathetic nerves | 23 | 23 | 100.00 |  |  |  |  |
| S346 | Injury of peripheral nerve(s) of abdomen, lower back and pelvis | 3 | 3 | 100.00 |  |  |  |  |
| S348 | Injury of other and unspecified nerves at abdomen, lower back and pelvis level | 17 | 16 | 94.12 |  |  |  |  |
| S35 | Injury of blood vessels at abdomen, lower back and pelvis level | 1625 | 1624 | 99.94 |  |  |  |  |
| S350 | Injury of abdominal aorta | 71 | 71 | 100.00 |  |  |  |  |
| S351 | Injury of inferior vena cava | 104 | 104 | 100.00 |  |  |  |  |
| S352 | Injury of coeliac or mesenteric artery | 430 | 430 | 100.00 |  |  |  |  |
| S353 | Injury of portal or splenic vein | 140 | 140 | 100.00 |  |  |  |  |
| S354 | Injury of renal blood vessels | 188 | 188 | 100.00 |  |  |  |  |
| S355 | Injury of iliac blood vessels | 139 | 139 | 100.00 |  |  |  |  |
| S357 | Injury of multiple blood vessels at abdomen, lower back and pelvis level | 30 | 30 | 100.00 |  |  |  |  |
| S358 | Injury of other blood vessels at abdomen, lower back and pelvis level | 245 | 245 | 100.00 |  |  |  |  |
| S359 | Injury of unspecified blood vessel at abdomen, lower back and pelvis level | 278 | 277 | 99.64 |  |  |  |  |
| S36 | Injury of intra-abdominal organs | 16949 | 16934 | 99.91 |  |  |  |  |
| S360 | Injury of spleen | 3748 | 3741 | 99.81 |  |  |  |  |
| S361 | Injury of liver or gallbladder | 7181 | 7177 | 99.94 |  |  |  |  |
| S362 | Injury of pancreas | 599 | 599 | 100.00 |  |  |  |  |
| S363 | Injury of stomach | 80 | 80 | 100.00 |  |  |  |  |
| S364 | Injury of small intestine | 635 | 635 | 100.00 |  |  |  |  |
| S365 | Injury of colon | 259 | 259 | 100.00 |  |  |  |  |
| S366 | Injury of rectum | 160 | 160 | 100.00 |  |  |  |  |
| S367 | Injury of multiple intra-abdominal organs | 129 | 129 | 100.00 |  |  |  |  |
| S368 | Injury of other intra-abdominal organs | 3407 | 3404 | 99.91 |  |  |  |  |
| S369 | Injury of unspecified intra-abdominal organ | 751 | 750 | 99.87 |  |  |  |  |
| S37 | Injury of urinary and pelvic organs | 6296 | 6289 | 99.89 |  |  |  |  |
| S370 | Injury of kidney | 3102 | 3096 | 99.81 |  |  |  |  |
| S371 | Injury of ureter | 90 | 90 | 100.00 |  |  |  |  |
| S372 | Injury of bladder | 571 | 571 | 100.00 |  |  |  |  |
| S373 | Injury of urethra | 1859 | 1858 | 99.95 |  |  |  |  |
| S374 | Injury of ovary | 11 | 11 | 100.00 |  |  |  |  |
| S375 | Injury of fallopian tube | 2 | 2 | 100.00 |  |  |  |  |
| S376 | Injury of uterus | 22 | 22 | 100.00 |  |  |  |  |
| S377 | Injury of multiple pelvic organs | 37 | 37 | 100.00 |  |  |  |  |
| S378 | Injury of other pelvic organs | 481 | 481 | 100.00 |  |  |  |  |
| S379 | Injury of unspecified pelvic organ | 121 | 121 | 100.00 |  |  |  |  |
| S38 | Crushing injury and traumatic amputation of part of abdomen, lower back and pelvis | 117 | 117 | 100.00 |  |  |  |  |
| S380 | Crushing injury of external genital organs | 59 | 59 | 100.00 |  |  |  |  |
| S381 | Crushing injury of other and unspecified parts of abdomen, lower back and pelvis | 25 | 25 | 100.00 |  |  |  |  |
| S382 | Traumatic amputation of external genital organs | 32 | 32 | 100.00 |  |  |  |  |
| S383 | Traumatic amputation of other and unspecified parts of abdomen, lower back and pelvis | 1 | 1 | 100.00 |  |  |  |  |
| S39 | Other and unspecified injuries of abdomen, lower back and pelvis | 4028 | 4025 | 99.93 |  |  |  |  |
| S390 | Injury of muscle and tendon of abdomen, lower back and pelvis | 196 | 196 | 100.00 |  |  |  |  |
| S396 | Injury of intra-abdominal organs(s) with pelvic organ(s) | 48 | 48 | 100.00 |  |  |  |  |
| S397 | Other multiple injuries of abdomen, lower back and pelvis | 35 | 35 | 100.00 |  |  |  |  |
| S398 | Other specified injuries of abdomen, lower back and pelvis | 255 | 255 | 100.00 |  |  |  |  |
| S399 | Unspecified injury of abdomen, lower back and pelvis | 3494 | 3491 | 99.91 |  |  |  |  |
| S40 | Superficial injury of shoulder and upper arm | 145371 | 145230 | 99.90 |  |  |  |  |
| S400 | Contusion of shoulder and upper arm | 138861 | 138726 | 99.90 |  |  |  |  |
| S407 | Multiple superficial injuries of shoulder and upper arm | 308 | 308 | 100.00 |  |  |  |  |
| S408 | Other superficial injuries of shoulder and upper arm | 4910 | 4905 | 99.90 |  |  |  |  |
| S409 | Superficial injury of shoulder and upper arm, unspecified | 1292 | 1291 | 99.92 |  |  |  |  |
| S41 | Open wound of shoulder and upper arm | 8436 | 8423 | 99.85 |  |  |  |  |
| S410 | Open wound of shoulder | 1880 | 1878 | 99.89 |  |  |  |  |
| S411 | Open wound of upper arm | 5816 | 5807 | 99.85 |  |  |  |  |
| S417 | Multiple open wounds of shoulder and upper arm | 547 | 546 | 99.82 |  |  |  |  |
| S418 | Open wound of other and unspecified parts of shoulder girdle | 193 | 192 | 99.48 |  |  |  |  |
| S42 | Fracture of shoulder and upper arm | 116508 | 116399 | 99.91 |  |  |  |  |
| S420 | Fracture of clavicle | 46697 | 46650 | 99.90 |  |  |  |  |
| S421 | Fracture of scapula | 9560 | 9549 | 99.88 |  |  |  |  |
| S422 | Fracture of upper end of humerus | 20125 | 20104 | 99.90 |  |  |  |  |
| S423 | Fracture of shaft of humerus | 17626 | 17612 | 99.92 |  |  |  |  |
| S424 | Fracture of lower end of humerus | 21061 | 21046 | 99.93 |  |  |  |  |
| S427 | Multiple fractures of clavicle, scapula and humerus | 77 | 76 | 98.70 |  |  |  |  |
| S428 | Fracture of other parts of shoulder and upper arm | 480 | 480 | 100.00 |  |  |  |  |
| S429 | Fracture of shoulder girdle, part unspecified | 882 | 882 | 100.00 |  |  |  |  |
| S43 | Dislocation, sprain and strain of joints and ligaments of shoulder girdle | 73760 | 73699 | 99.92 |  |  |  |  |
| S430 | Dislocation of shoulder joint | 25244 | 25218 | 99.90 |  |  |  |  |
| S431 | Anterior dislocation of humerus | 1730 | 1727 | 99.83 |  |  |  |  |
| S432 | Dislocation of sternoclavicular joint | 92 | 92 | 100.00 |  |  |  |  |
| S433 | Dislocation of other and unspecified parts of shoulder girdle | 1946 | 1944 | 99.90 |  |  |  |  |
| S434 | Sprain and strain of shoulder joint | 33417 | 33394 | 99.93 |  |  |  |  |
| S435 | Sprain and strain of acromioclavicular joint | 2385 | 2384 | 99.96 |  |  |  |  |
| S436 | Sprain and strain of sternoclavicular joint | 79 | 79 | 100.00 |  |  |  |  |
| S437 | Sprain and strain of other and unspecified | 8867 | 8861 | 99.93 |  |  |  |  |
| S439 | parts of shoulder girdle | 0 | 0 | 0.00 |  |  |  |  |
| S44 | Injury of nerves at shoulder and upper arm level | 312 | 311 | 99.68 |  |  |  |  |
| S440 | Injury of ulnar nerve at upper arm level | 18 | 18 | 100.00 |  |  |  |  |
| S441 | Injury of median nerve at upper arm level | 12 | 12 | 100.00 |  |  |  |  |
| S442 | Injury of radial nerve at upper arm level | 62 | 62 | 100.00 |  |  |  |  |
| S443 | Injury of axillary nerve | 154 | 154 | 100.00 |  |  |  |  |
| S444 | Injury of musculocutaneous nerve | 14 | 14 | 100.00 |  |  |  |  |
| S445 | Injury of cutaneous sensory nerve at shoulder and upper arm level | 4 | 4 | 100.00 |  |  |  |  |
| S447 | Injury of multiple nerves at shoulder and upper arm level | 4 | 4 | 100.00 |  |  |  |  |
| S448 | Injury of other nerves at shoulder and upper arm level | 15 | 14 | 93.33 |  |  |  |  |
| S449 | Injury of unspecified nerve at shoulder and upper arm level | 29 | 29 | 100.00 |  |  |  |  |
| S45 | Injury of blood vessels at shoulder and upper arm level | 367 | 365 | 99.46 |  |  |  |  |
| S450 | Injury of axillary artery | 24 | 24 | 100.00 |  |  |  |  |
| S451 | Injury of brachial artery | 77 | 76 | 98.70 |  |  |  |  |
| S452 | Injury of axillary or brachial vein | 24 | 23 | 95.83 |  |  |  |  |
| S453 | Injury of superficial vein at shoulder and upper arm level | 67 | 67 | 100.00 |  |  |  |  |
| S457 | Injury of multiple blood vessels at shoulder and upper arm level | 4 | 4 | 100.00 |  |  |  |  |
| S458 | Injury of other blood vessels at shoulder and upper arm level | 97 | 97 | 100.00 |  |  |  |  |
| S459 | Injury of unspecified blood vessel at shoulder and upper arm level | 74 | 74 | 100.00 |  |  |  |  |
| S46 | Injury of muscle and tendon at shoulder and upper arm level | 2619 | 2617 | 99.92 |  |  |  |  |
| S460 | Injury of muscle(s) and tendon(s) of the rotator cuff of shoulder | 1145 | 1145 | 100.00 |  |  |  |  |
| S461 | Injury of muscle and tendon of long head of biceps | 129 | 129 | 100.00 |  |  |  |  |
| S462 | Injury of muscle and tendon of other parts of biceps | 173 | 173 | 100.00 |  |  |  |  |
| S463 | Injury of muscle and tendon of triceps | 110 | 110 | 100.00 |  |  |  |  |
| S467 | Injury of multiple muscles and tendons at shoulder and upper arm level | 190 | 190 | 100.00 |  |  |  |  |
| S468 | Injury of other muscles and tendon at shoulder and upper arm level | 439 | 439 | 100.00 |  |  |  |  |
| S469 | Injury of unspecified muscle and tendon at shoulder and upper arm level | 433 | 431 | 99.54 |  |  |  |  |
| S47 | Crushing injury of shoulder and upper arm | 87 | 87 | 100.00 |  |  |  |  |
| S48 | Traumatic amputation of shoulder and upper arm | 196 | 196 | 100.00 |  |  |  |  |
| S480 | Traumatic amputation at shoulder joint | 117 | 117 | 100.00 |  |  |  |  |
| S481 | Traumatic amputation at level between shoulder and elbow | 23 | 23 | 100.00 |  |  |  |  |
| S489 | Traumatic amputation of shoulder and upper arm, level unspecified | 56 | 56 | 100.00 |  |  |  |  |
| S49 | Other and unspecified injuries of shoulder and upper arm | 2646 | 2644 | 99.92 |  |  |  |  |
| S497 | Multiple injuries of shoulder and upper arm | 28 | 28 | 100.00 |  |  |  |  |
| S498 | Other specified injuries of shoulder and upper arm | 278 | 278 | 100.00 |  |  |  |  |
| S499 | Unspecified injury of shoulder and upper arm | 2337 | 2335 | 99.91 |  |  |  |  |
| S50 | Superficial injury of forearm | 164322 | 164136 | 99.89 |  |  |  |  |
| S500 | Contusion of elbow | 104716 | 104605 | 99.89 |  |  |  |  |
| S501 | Contusion of other and unspecified parts of forearm | 37675 | 37626 | 99.87 |  |  |  |  |
| S507 | Multiple superficial injuries of forearm | 1381 | 1381 | 100.00 |  |  |  |  |
| S508 | Other superficial injuries of forearm | 14564 | 14552 | 99.92 |  |  |  |  |
| S509 | Superficial injury of forearm, unspecified | 5986 | 5972 | 99.77 |  |  |  |  |
| S51 | Open wound of forearm | 55970 | 55914 | 99.90 |  |  |  |  |
| S510 | Open wound of elbow | 15444 | 15426 | 99.88 |  |  |  |  |
| S517 | Multiple open wounds of forearm | 3108 | 3103 | 99.84 |  |  |  |  |
| S518 | Open wound of other parts of forearm | 12848 | 12838 | 99.92 |  |  |  |  |
| S519 | Open wound of forearm, part unspecified | 24570 | 24547 | 99.91 |  |  |  |  |
| S52 | Fracture of forearm | 149125 | 148980 | 99.90 |  |  |  |  |
| S520 | Fracture of upper end of ulna | 16784 | 16769 | 99.91 |  |  |  |  |
| S521 | Fracture of upper end of radius | 13007 | 12994 | 99.90 |  |  |  |  |
| S522 | Fracture of shaft of ulna | 4150 | 4147 | 99.93 |  |  |  |  |
| S523 | Fracture of shaft of radius | 6062 | 6057 | 99.92 |  |  |  |  |
| S524 | Fracture of shafts of both ulna and radius | 4318 | 4314 | 99.91 |  |  |  |  |
| S525 | Fracture of lower end of radius | 76836 | 76764 | 99.91 |  |  |  |  |
| S526 | Fracture of lower end of both ulna and radius | 15339 | 15317 | 99.86 |  |  |  |  |
| S527 | Multiple fracture of forearm | 671 | 670 | 99.85 |  |  |  |  |
| S528 | Fracture of other parts of forearm | 8375 | 8367 | 99.90 |  |  |  |  |
| S529 | Fracture of forearm, part unspecified | 3583 | 3581 | 99.94 |  |  |  |  |
| S53 | Dislocation, sprain and strain of joints and ligaments of elbow | 106469 | 106357 | 99.89 |  |  |  |  |
| S530 | Dislocation of radial head | 44606 | 44560 | 99.90 |  |  |  |  |
| S531 | Dislocation of elbow, unspecified | 43436 | 43390 | 99.89 |  |  |  |  |
| S532 | Traumatic rupture of radial collateral ligament | 89 | 89 | 100.00 |  |  |  |  |
| S533 | Traumatic rupture of ulnar collateral ligament | 135 | 135 | 100.00 |  |  |  |  |
| S534 | Sprain and strain of elbow | 18203 | 18183 | 99.89 |  |  |  |  |
| S54 | Injury of nerves at forearm level | 837 | 836 | 99.88 |  |  |  |  |
| S540 | Injury of ulnar nerve at forearm level | 192 | 192 | 100.00 |  |  |  |  |
| S541 | Injury of median nerve at forearm level | 115 | 115 | 100.00 |  |  |  |  |
| S542 | Injury of radial nerve at forearm level | 331 | 330 | 99.70 |  |  |  |  |
| S543 | Injury of cutaneous sensory nerve at forearm level | 26 | 26 | 100.00 |  |  |  |  |
| S547 | Injury of multiple nerves at forearm level | 6 | 6 | 100.00 |  |  |  |  |
| S548 | Injury of other nerves at forearm level | 28 | 28 | 100.00 |  |  |  |  |
| S549 | Injury of unspecified nerve at forearm level | 139 | 139 | 100.00 |  |  |  |  |
| S55 | Injury of blood vessels at forearm level | 1081 | 1081 | 100.00 |  |  |  |  |
| S550 | Injury of ulnar artery at forearm level | 67 | 67 | 100.00 |  |  |  |  |
| S551 | Injury of radial artery at forearm level | 162 | 162 | 100.00 |  |  |  |  |
| S552 | Injury of vein at forearm level | 111 | 111 | 100.00 |  |  |  |  |
| S557 | Injury of multiple blood vessels at forearm level | 10 | 10 | 100.00 |  |  |  |  |
| S558 | Injury of other blood vessels at forearm level | 473 | 473 | 100.00 |  |  |  |  |
| S559 | Injury of unspecified blood vessels at forearm level | 258 | 258 | 100.00 |  |  |  |  |
| S56 | Injury of muscle and tendon at forearm level | 4568 | 4564 | 99.91 |  |  |  |  |
| S560 | Injury of flexor muscle and tendon of thumb at forearm level | 175 | 175 | 100.00 |  |  |  |  |
| S561 | Injury of flexor muscle and tendon of other finger(s) at forearm level | 712 | 712 | 100.00 |  |  |  |  |
| S562 | Injury of other flexor muscle and tendon at forearm level | 626 | 625 | 99.84 |  |  |  |  |
| S563 | Injury of extensor or abductor muscles and tendons of thumb at forearm level | 249 | 249 | 100.00 |  |  |  |  |
| S564 | Injury of extensor muscle and tendon of other finger(s) at forearm level | 1197 | 1196 | 99.92 |  |  |  |  |
| S565 | Injury of other extensor muscle and tendon at forearm level | 361 | 361 | 100.00 |  |  |  |  |
| S567 | Injury of multiple muscles and tendons at forearm level | 308 | 308 | 100.00 |  |  |  |  |
| S568 | Injury of other and unspecified muscles and tendon at forearm level | 940 | 938 | 99.79 |  |  |  |  |
| S57 | Crushing injury of forearm | 414 | 414 | 100.00 |  |  |  |  |
| S570 | Crushing injury of elbow | 127 | 127 | 100.00 |  |  |  |  |
| S578 | Crushing injury of other parts of forearm | 116 | 116 | 100.00 |  |  |  |  |
| S579 | Crushing injury of forearm, part unspecified | 171 | 171 | 100.00 |  |  |  |  |
| S58 | Traumatic amputation of forearm | 147 | 147 | 100.00 |  |  |  |  |
| S580 | Traumatic amputation at elbow level | 59 | 59 | 100.00 |  |  |  |  |
| S581 | Traumatic amputation at level between elbow and wrist | 19 | 19 | 100.00 |  |  |  |  |
| S589 | Traumatic amputation of forearm, level unspecified | 69 | 69 | 100.00 |  |  |  |  |
| S59 | Other and unspecified injuries of forearm | 3603 | 3600 | 99.92 |  |  |  |  |
| S597 | Multiple injuries of forearm | 49 | 49 | 100.00 |  |  |  |  |
| S598 | Other specified injuries of forearm | 250 | 250 | 100.00 |  |  |  |  |
| S599 | Unspecified injury of forearm | 3297 | 3294 | 99.91 |  |  |  |  |
| S60 | Superficial injury of wrist and hand | 315810 | 315485 | 99.90 |  |  |  |  |
| S600 | Contusion of finger(s) without damage to nail | 101075 | 100974 | 99.90 |  |  |  |  |
| S601 | Contusion of finger(s) with damage to nail | 15030 | 15022 | 99.95 |  |  |  |  |
| S602 | Contusion of other parts of wrist and hand | 137379 | 137244 | 99.90 |  |  |  |  |
| S607 | Multiple superficial injuries of wrist and hand | 1822 | 1819 | 99.84 |  |  |  |  |
| S608 | Other superficial injuries of wrist and hand | 42717 | 42670 | 99.89 |  |  |  |  |
| S609 | Superficial injury of wrist and hand, unspecified | 17787 | 17756 | 99.83 |  |  |  |  |
| S61 | Open wound of wrist and hand | 602914 | 602349 | 99.91 |  |  |  |  |
| S610 | Open wound of finger(s) without damage to nail | 389722 | 389339 | 99.90 |  |  |  |  |
| S611 | Open wound of finger(s) with damage to nail | 41041 | 41016 | 99.94 |  |  |  |  |
| S617 | Multiple open wounds of wrist and hand | 10264 | 10254 | 99.90 |  |  |  |  |
| S618 | Open wound of other parts of wrist and hand | 73385 | 73325 | 99.92 |  |  |  |  |
| S619 | Open wound of wrist and hand, part unspecified | 88502 | 88415 | 99.90 |  |  |  |  |
| S62 | Fracture at wrist and hand level | 124571 | 124450 | 99.90 |  |  |  |  |
| S620 | Fracture of navicular [scaphoid] bone of hand | 2682 | 2677 | 99.81 |  |  |  |  |
| S621 | Fracture of other carpal bone(s) | 5000 | 4996 | 99.92 |  |  |  |  |
| S622 | Fracture of first metacarpal bone | 2284 | 2282 | 99.91 |  |  |  |  |
| S623 | Fracture of other metacarpal bone | 22033 | 22009 | 99.89 |  |  |  |  |
| S624 | Multiple fracture of metacarpal bones | 1070 | 1070 | 100.00 |  |  |  |  |
| S625 | Fracture of thumb | 8256 | 8245 | 99.87 |  |  |  |  |
| S626 | Fracture of other finger | 70528 | 70465 | 99.91 |  |  |  |  |
| S627 | Multiple fracture of fingers | 2194 | 2190 | 99.82 |  |  |  |  |
| S628 | Fracture of other and unspecified parts of wrist and hand | 10524 | 10516 | 99.92 |  |  |  |  |
| S63 | Dislocation, sprain and strain of joints and ligaments at wrist and hand level | 84912 | 84836 | 99.91 |  |  |  |  |
| S630 | Dislocation of wrist | 1457 | 1453 | 99.73 |  |  |  |  |
| S631 | Dislocation of finger | 8372 | 8364 | 99.90 |  |  |  |  |
| S632 | Multiple dislocations of fingers | 72 | 72 | 100.00 |  |  |  |  |
| S633 | Traumatic rupture of ligament of wrist and carpus | 235 | 235 | 100.00 |  |  |  |  |
| S634 | Traumatic rupture of ligament of finger at metacarpophalangeal and interphalangeal joint(s) | 853 | 852 | 99.88 |  |  |  |  |
| S635 | Sprain and strain of wrist | 42742 | 42702 | 99.91 |  |  |  |  |
| S636 | Sprain and strain of finger(s) | 25480 | 25464 | 99.94 |  |  |  |  |
| S637 | Sprain and strain of other and unspecified parts of hand | 5701 | 5694 | 99.88 |  |  |  |  |
| S64 | Injury of nerves at wrist and hand level | 2039 | 2036 | 99.85 |  |  |  |  |
| S640 | Injury of ulnar nerve at wrist and hand level | 116 | 116 | 100.00 |  |  |  |  |
| S641 | Injury of median nerve at wrist and hand level | 135 | 135 | 100.00 |  |  |  |  |
| S642 | Injury of radial nerve at wrist and hand level | 122 | 122 | 100.00 |  |  |  |  |
| S643 | Injury of digital nerve of thumb | 244 | 244 | 100.00 |  |  |  |  |
| S644 | Injury of digital nerve of other finger | 1157 | 1154 | 99.74 |  |  |  |  |
| S647 | Injury of multiple nerves at wrist and hand level | 13 | 13 | 100.00 |  |  |  |  |
| S648 | Injury of other nerves at wrist and hand level | 110 | 110 | 100.00 |  |  |  |  |
| S649 | Injury of unspecified nerve at wrist and hand level | 142 | 142 | 100.00 |  |  |  |  |
| S65 | Injury of blood vessels at wrist and hand level | 13878 | 13868 | 99.93 |  |  |  |  |
| S650 | Injury of ulnar artery at wrist and hand level | 155 | 155 | 100.00 |  |  |  |  |
| S651 | Injury of radial artery at wrist and hand level | 210 | 210 | 100.00 |  |  |  |  |
| S652 | Injury of superficial palmar arch | 589 | 589 | 100.00 |  |  |  |  |
| S653 | Injury of deep palmar arch | 176 | 175 | 99.43 |  |  |  |  |
| S654 | Injury of blood vessel(s) of thumb | 760 | 760 | 100.00 |  |  |  |  |
| S655 | Injury of blood vessel(s) of other finger | 9268 | 9260 | 99.91 |  |  |  |  |
| S657 | Injury of multiple blood vessels at wrist and hand level | 15 | 15 | 100.00 |  |  |  |  |
| S658 | Injury of other blood vessels at wrist and hand level | 1717 | 1717 | 100.00 |  |  |  |  |
| S659 | Injury of unspecified blood vessel at wrist and hand level | 988 | 987 | 99.90 |  |  |  |  |
| S66 | Injury of muscle and tendon at wrist and hand level | 23506 | 23485 | 99.91 |  |  |  |  |
| S660 | Injury of long flexor muscle and tendon of thumb at wrist and hand level | 460 | 460 | 100.00 |  |  |  |  |
| S661 | Injury of flexor muscle and tendon of other finger at wrist and hand level | 3585 | 3583 | 99.94 |  |  |  |  |
| S662 | Injury of extensor muscle and tendon of thumb at wrist and hand level | 2147 | 2143 | 99.81 |  |  |  |  |
| S663 | Injury of extensor muscle and tendon of other finger at wrist and hand level | 10365 | 10356 | 99.91 |  |  |  |  |
| S664 | Injury of intrinsic muscle and tendon of thumb at wrist and hand level | 271 | 270 | 99.63 |  |  |  |  |
| S665 | Injury of intrinsic muscle and tendon of other finger at wrist and hand level | 1176 | 1175 | 99.91 |  |  |  |  |
| S666 | Injury of multiple flexor muscles and tendons at wrist and hand level | 489 | 489 | 100.00 |  |  |  |  |
| S667 | Injury of multiple extensor muscles and tendons at wrist and hand level | 508 | 508 | 100.00 |  |  |  |  |
| S668 | Injury of other muscles and tendons at wrist and hand level | 1803 | 1800 | 99.83 |  |  |  |  |
| S669 | Injury of unspecified muscle and tendon at wrist and hand level | 2702 | 2701 | 99.96 |  |  |  |  |
| S67 | Crushing injury of wrist and hand | 8909 | 8901 | 99.91 |  |  |  |  |
| S670 | Crushing injury of thumb and other finger(s) | 7635 | 7628 | 99.91 |  |  |  |  |
| S678 | Crushing injury of other and unspecified parts of wrist and hand | 1274 | 1273 | 99.92 |  |  |  |  |
| S68 | Traumatic amputation of wrist and hand | 8876 | 8864 | 99.86 |  |  |  |  |
| S680 | Traumatic amputation of thumb (complete) (partial) | 937 | 936 | 99.89 |  |  |  |  |
| S681 | Traumatic amputation of other single finger (complete)(partial) | 6308 | 6299 | 99.86 |  |  |  |  |
| S682 | Traumatic amputation of two or more fingers alone (complete)(partial) | 663 | 662 | 99.85 |  |  |  |  |
| S683 | Combined traumatic amputation of (part of) finger(s) with other parts of wrist and hand | 503 | 503 | 100.00 |  |  |  |  |
| S684 | Traumatic amputation of hand at wrist level | 63 | 63 | 100.00 |  |  |  |  |
| S688 | Traumatic amputation of other parts of wrist and hand | 188 | 188 | 100.00 |  |  |  |  |
| S689 | Traumatic amputation of wrist and hand, level unspecified | 214 | 213 | 99.53 |  |  |  |  |
| S69 | Other and unspecified injuries of wrist and hand | 20202 | 20184 | 99.91 |  |  |  |  |
| S697 | Multiple injuries of wrist and hand | 179 | 179 | 100.00 |  |  |  |  |
| S698 | Other specified injuries of wrist and hand | 913 | 912 | 99.89 |  |  |  |  |
| S699 | Unspecified injury of wrist and hand | 19105 | 19088 | 99.91 |  |  |  |  |
| S70 | Superficial injury of hip and thigh | 112479 | 112387 | 99.92 |  |  |  |  |
| S700 | Contusion of hip | 77187 | 77121 | 99.91 |  |  |  |  |
| S701 | Contusion of thigh | 28275 | 28254 | 99.93 |  |  |  |  |
| S707 | Multiple superficial injuries of hip and thigh | 570 | 570 | 100.00 |  |  |  |  |
| S708 | Other superficial injuries of hip and thigh | 4725 | 4721 | 99.92 |  |  |  |  |
| S709 | Superficial injury of hip and thigh, unspecified | 1722 | 1721 | 99.94 |  |  |  |  |
| S71 | Open wound of hip and thigh | 14745 | 14731 | 99.91 |  |  |  |  |
| S710 | Open wound of hip | 1713 | 1711 | 99.88 |  |  |  |  |
| S711 | Open wound of thigh | 12534 | 12522 | 99.90 |  |  |  |  |
| S717 | Multiple open wounds of hip and thigh | 341 | 341 | 100.00 |  |  |  |  |
| S718 | Open wound of other and unspecified parts of pelvic girdle | 157 | 157 | 100.00 |  |  |  |  |
| S72 | Fracture of femur | 122557 | 122447 | 99.91 |  |  |  |  |
| S720 | Fracture of neck of femur | 41075 | 41047 | 99.93 |  |  |  |  |
| S721 | Pertrochanteric fracture | 42513 | 42462 | 99.88 |  |  |  |  |
| S722 | Subtrochanteric fracture | 2525 | 2523 | 99.92 |  |  |  |  |
| S723 | Fracture of shaft of femur | 10880 | 10873 | 99.94 |  |  |  |  |
| S724 | Fracture of lower end of femur | 5965 | 5959 | 99.90 |  |  |  |  |
| S727 | Multiple fractures of femur | 213 | 213 | 100.00 |  |  |  |  |
| S728 | Fractures of other parts of femur | 2980 | 2977 | 99.90 |  |  |  |  |
| S729 | Fracture of femur, part unspecified | 16406 | 16393 | 99.92 |  |  |  |  |
| S73 | Dislocation, sprain and strain of joint and ligaments of hip | 15139 | 15120 | 99.87 |  |  |  |  |
| S730 | Dislocation of hip | 5562 | 5557 | 99.91 |  |  |  |  |
| S731 | Sprain and strain of hip | 9577 | 9563 | 99.85 |  |  |  |  |
| S74 | Injury of nerves at hip and thigh level | 85 | 85 | 100.00 |  |  |  |  |
| S740 | Injury of sciatic nerve at hip and thigh level | 52 | 52 | 100.00 |  |  |  |  |
| S741 | Injury of femoral nerve at hip and thigh level | 13 | 13 | 100.00 |  |  |  |  |
| S742 | Injury of cutaneous sensory nerve at hip and thigh level | 0 | 0 | 0.00 |  |  |  |  |
| S747 | Injury of multiple nerves at hip and thigh level | 2 | 2 | 100.00 |  |  |  |  |
| S748 | Injury of other nerves at hip and thigh level | 11 | 11 | 100.00 |  |  |  |  |
| S749 | Injury of unspecified nerve at hip and thigh level | 7 | 7 | 100.00 |  |  |  |  |
| S75 | Injury of blood vessels at hip and thigh level | 602 | 601 | 99.83 |  |  |  |  |
| S750 | Injury of femoral artery | 124 | 124 | 100.00 |  |  |  |  |
| S751 | Injury of femoral vein at hip and thigh level | 58 | 58 | 100.00 |  |  |  |  |
| S752 | Injury of greater saphenous vein at hip and thigh level | 5 | 5 | 100.00 |  |  |  |  |
| S757 | Injury of multiple blood vessels at hip and thigh level | 7 | 7 | 100.00 |  |  |  |  |
| S758 | Injury of other blood vessels at hip and thigh level | 192 | 192 | 100.00 |  |  |  |  |
| S759 | Injury of unspecified blood vessel at hip and thigh level | 216 | 215 | 99.54 |  |  |  |  |
| S76 | Injury of muscle and tendon at hip and thigh level | 3081 | 3079 | 99.94 |  |  |  |  |
| S760 | Injury of muscle and tendon of hip | 196 | 196 | 100.00 |  |  |  |  |
| S761 | Injury of quadriceps muscle and tendon | 1322 | 1322 | 100.00 |  |  |  |  |
| S762 | Injury of adductor muscle and tendon of thigh | 149 | 149 | 100.00 |  |  |  |  |
| S763 | Injury of muscle and tendon of the posterior muscle group at thigh level | 329 | 327 | 99.39 |  |  |  |  |
| S764 | Injury of other and unspecified muscles and tendons at thigh level | 883 | 883 | 100.00 |  |  |  |  |
| S767 | Injury of multiple muscles and tendons at hip and thigh level | 202 | 202 | 100.00 |  |  |  |  |
| S77 | Crushing injury of hip and thigh | 112 | 112 | 100.00 |  |  |  |  |
| S770 | Crushing injury of hip | 13 | 13 | 100.00 |  |  |  |  |
| S771 | Crushing injury of thigh | 83 | 83 | 100.00 |  |  |  |  |
| S772 | Crushing injury of hip with thigh | 16 | 16 | 100.00 |  |  |  |  |
| S78 | Traumatic amputation of hip and thigh | 24 | 24 | 100.00 |  |  |  |  |
| S780 | Traumatic amputation at hip joint | 2 | 2 | 100.00 |  |  |  |  |
| S781 | Traumatic amputation at level between hip and knee | 13 | 13 | 100.00 |  |  |  |  |
| S789 | Traumatic amputation of hip and thigh, level unspecified | 9 | 9 | 100.00 |  |  |  |  |
| S79 | Other and unspecified injuries of hip and thigh | 1603 | 1600 | 99.81 |  |  |  |  |
| S797 | Multiple injuries of hip and thigh | 16 | 16 | 100.00 |  |  |  |  |
| S798 | Other specified injuries of hip and thigh | 147 | 146 | 99.32 |  |  |  |  |
| S799 | Unspecified injury of hip and thigh | 1439 | 1437 | 99.86 |  |  |  |  |
| S80 | Superficial injury of lower leg | 330946 | 330644 | 99.91 |  |  |  |  |
| S800 | Contusion of knee | 213764 | 213579 | 99.91 |  |  |  |  |
| S801 | Contusion of other and unspecified parts of lower leg | 79351 | 79276 | 99.91 |  |  |  |  |
| S807 | Multiple superficial injuries of lower leg | 2560 | 2555 | 99.80 |  |  |  |  |
| S808 | Other superficial injuries of lower leg | 28588 | 28558 | 99.90 |  |  |  |  |
| S809 | Superficial injury of lower leg, unspecified | 6683 | 6676 | 99.90 |  |  |  |  |
| S81 | Open wound of lower leg | 104289 | 104175 | 99.89 |  |  |  |  |
| S810 | Open wound of knee | 45542 | 45486 | 99.88 |  |  |  |  |
| S817 | Multiple open wounds of lower leg | 2623 | 2620 | 99.89 |  |  |  |  |
| S818 | Open wound of other parts of lower leg | 21550 | 21530 | 99.91 |  |  |  |  |
| S819 | Open wound of lower leg, part unspecified | 34574 | 34539 | 99.90 |  |  |  |  |
| S82 | Fracture of lower leg, including ankle | 135972 | 135850 | 99.91 |  |  |  |  |
| S820 | Fracture of patella | 18260 | 18238 | 99.88 |  |  |  |  |
| S821 | Fracture of upper end of tibia | 11982 | 11970 | 99.90 |  |  |  |  |
| S822 | Fracture of shaft of tibia | 18883 | 18867 | 99.92 |  |  |  |  |
| S823 | Fracture of lower end of tibia | 15601 | 15586 | 99.90 |  |  |  |  |
| S824 | Fracture of fibula alone | 15188 | 15175 | 99.91 |  |  |  |  |
| S825 | Fracture of medial malleolus | 9242 | 9234 | 99.91 |  |  |  |  |
| S826 | Fracture of lateral malleolus | 24764 | 24745 | 99.92 |  |  |  |  |
| S827 | Multiple fractures of lower leg | 321 | 321 | 100.00 |  |  |  |  |
| S828 | Fractures of other parts of lower leg | 19567 | 19554 | 99.93 |  |  |  |  |
| S829 | Fracture of lower leg, part unspecified | 2164 | 2160 | 99.82 |  |  |  |  |
| S83 | Dislocation, sprain and strain of joints and ligaments of knee | 48297 | 48243 | 99.89 |  |  |  |  |
| S830 | Dislocation of patella | 1401 | 1399 | 99.86 |  |  |  |  |
| S831 | Dislocation of knee | 1020 | 1018 | 99.80 |  |  |  |  |
| S832 | Tear of meniscus, current | 1471 | 1471 | 100.00 |  |  |  |  |
| S833 | Tear of articular cartilage of knee, current | 57 | 57 | 100.00 |  |  |  |  |
| S834 | Sprain and strain involving (fibular)(tibial) collateral ligament of knee | 2816 | 2814 | 99.93 |  |  |  |  |
| S835 | Sprain and strain involving (anterior) (posterior) cruciate ligament of knee | 2520 | 2519 | 99.96 |  |  |  |  |
| S836 | Sprain and strain of other and unspecified parts of knee | 37967 | 37921 | 99.88 |  |  |  |  |
| S837 | Injury to multiple structures of knee | 1045 | 1044 | 99.90 |  |  |  |  |
| S84 | Injury of nerves at lower leg level | 192 | 192 | 100.00 |  |  |  |  |
| S840 | Injury of tibial nerve at lower leg level | 32 | 32 | 100.00 |  |  |  |  |
| S841 | Injury of peroneal nerve at lower leg level | 117 | 117 | 100.00 |  |  |  |  |
| S842 | Injury of cutaneous sensory nerve at lower leg level | 11 | 11 | 100.00 |  |  |  |  |
| S847 | Injury of multiple nerves at lower leg level | 0 | 0 | 0.00 |  |  |  |  |
| S848 | Injury of other nerves at lower leg level | 17 | 17 | 100.00 |  |  |  |  |
| S849 | Injury of unspecified nerve at lower leg level | 15 | 15 | 100.00 |  |  |  |  |
| S85 | Injury of blood vessels at lower leg level | 1857 | 1857 | 100.00 |  |  |  |  |
| S850 | Injury of popliteal artery | 378 | 378 | 100.00 |  |  |  |  |
| S851 | Injury of (anterior)(posterior) tibial artery | 107 | 107 | 100.00 |  |  |  |  |
| S852 | Injury of peroneal artery | 8 | 8 | 100.00 |  |  |  |  |
| S853 | Injury of greater saphenous vein at lower leg level | 11 | 11 | 100.00 |  |  |  |  |
| S854 | Injury of lesser saphenous vein at lower leg level | 5 | 5 | 100.00 |  |  |  |  |
| S855 | Injury of popliteal vein | 14 | 14 | 100.00 |  |  |  |  |
| S857 | Injury of multiple blood vessels at lower leg level | 16 | 16 | 100.00 |  |  |  |  |
| S858 | Injury of other blood vessels at lower leg level | 742 | 742 | 100.00 |  |  |  |  |
| S859 | Injury of unspecified blood vessel at lower leg level | 576 | 576 | 100.00 |  |  |  |  |
| S86 | Injury of muscle and tendon at lower leg level | 14408 | 14397 | 99.92 |  |  |  |  |
| S860 | Injury of Achilles tendon | 7735 | 7730 | 99.94 |  |  |  |  |
| S861 | Injury of other muscle(s) and tendon(s) of posterior muscle group at lower leg level | 1150 | 1149 | 99.91 |  |  |  |  |
| S862 | Injury of muscle(s) and tendon(s) of anterior muscle group at lower leg level | 467 | 466 | 99.79 |  |  |  |  |
| S863 | Injury of muscle(s) and tendon(s) of peroneal muscle group at lower leg level | 279 | 279 | 100.00 |  |  |  |  |
| S867 | Injury of multiple muscles and tendons at lower leg level | 585 | 584 | 99.83 |  |  |  |  |
| S868 | Injury of other muscles and tendons at lower leg level | 1768 | 1767 | 99.94 |  |  |  |  |
| S869 | Injury of unspecified muscles and tendons at lower leg level | 2424 | 2422 | 99.92 |  |  |  |  |
| S87 | Crushing injury of lower leg | 723 | 722 | 99.86 |  |  |  |  |
| S870 | Crushing injury of knee | 339 | 339 | 100.00 |  |  |  |  |
| S878 | Crushing injury of other and unspecified parts of lower leg | 384 | 383 | 99.74 |  |  |  |  |
| S88 | Traumatic amputation of lower leg | 301 | 300 | 99.67 |  |  |  |  |
| S880 | Traumatic amputation at knee level | 28 | 28 | 100.00 |  |  |  |  |
| S881 | Traumatic amputation at level between knee and ankle | 51 | 50 | 98.04 |  |  |  |  |
| S889 | Traumatic amputation of lower leg, level unspecified | 222 | 222 | 100.00 |  |  |  |  |
| S89 | Other and unspecified injuries of lower leg | 5812 | 5806 | 99.90 |  |  |  |  |
| S897 | Multiple injuries of lower leg | 55 | 55 | 100.00 |  |  |  |  |
| S898 | Other specified injuries of lower leg | 453 | 453 | 100.00 |  |  |  |  |
| S899 | Unspecified injury of lower leg | 5303 | 5297 | 99.89 |  |  |  |  |
| S90 | Superficial injury of ankle and foot | 214107 | 213894 | 99.90 |  |  |  |  |
| S900 | Contusion of ankle | 53667 | 53610 | 99.89 |  |  |  |  |
| S901 | Contusion of toe(s) without damage to nail | 36846 | 36812 | 99.91 |  |  |  |  |
| S902 | Contusion of toe(s) with damage to nail | 14015 | 13998 | 99.88 |  |  |  |  |
| S903 | Contusion of other and unspecified parts of foot | 82234 | 82150 | 99.90 |  |  |  |  |
| S907 | Multiple superficial injuries of ankle and foot | 905 | 904 | 99.89 |  |  |  |  |
| S908 | Other superficial injuries of ankle and foot | 19594 | 19583 | 99.94 |  |  |  |  |
| S909 | Superficial injury of ankle and foot, unspecified | 6846 | 6837 | 99.87 |  |  |  |  |
| S91 | Open wound of ankle and foot | 105726 | 105624 | 99.90 |  |  |  |  |
| S910 | Open wound of ankle | 12518 | 12505 | 99.90 |  |  |  |  |
| S911 | Open wound of toe(s) without damage to nail | 24966 | 24939 | 99.89 |  |  |  |  |
| S912 | Open wound of toe(s) with damage to nail | 7902 | 7888 | 99.82 |  |  |  |  |
| S913 | Open wound of other parts of foot | 58804 | 58758 | 99.92 |  |  |  |  |
| S917 | Multiple open wounds of ankle and foot | 1536 | 1534 | 99.87 |  |  |  |  |
| S92 | Fracture of foot, except ankle | 98135 | 98021 | 99.88 |  |  |  |  |
| S920 | Fracture of calcaneus | 15744 | 15730 | 99.91 |  |  |  |  |
| S921 | Fracture of talus | 2969 | 2967 | 99.93 |  |  |  |  |
| S922 | Fracture of other tarsal bone(s) | 7390 | 7381 | 99.88 |  |  |  |  |
| S923 | Fracture of metatarsal bone | 32455 | 32423 | 99.90 |  |  |  |  |
| S924 | Fracture of great toe | 11499 | 11480 | 99.83 |  |  |  |  |
| S925 | Fracture of other toe | 22386 | 22354 | 99.86 |  |  |  |  |
| S927 | Multiple fractures of foot | 822 | 822 | 100.00 |  |  |  |  |
| S929 | Fracture of foot, unspecified | 4870 | 4864 | 99.88 |  |  |  |  |
| S93 | Dislocation, sprain and strain of joints and ligaments at ankle and foot level | 225995 | 225773 | 99.90 |  |  |  |  |
| S930 | Dislocation of ankle joint | 1615 | 1611 | 99.75 |  |  |  |  |
| S931 | Dislocation of toe(s) | 2034 | 2034 | 100.00 |  |  |  |  |
| S932 | Rupture of ligaments at ankle and foot level | 1036 | 1034 | 99.81 |  |  |  |  |
| S933 | Dislocation of other and unspecified parts of foot | 664 | 663 | 99.85 |  |  |  |  |
| S934 | Sprain and strain of ankle | 189778 | 189596 | 99.90 |  |  |  |  |
| S935 | Sprain and strain of toe(s) | 6373 | 6368 | 99.92 |  |  |  |  |
| S936 | Sprain and strain of other and unspecified parts of foot | 24495 | 24467 | 99.89 |  |  |  |  |
| S94 | Injury of nerves at ankle and foot level | 121 | 121 | 100.00 |  |  |  |  |
| S940 | Injury of lateral plantar nerve | 2 | 2 | 100.00 |  |  |  |  |
| S941 | Injury of medial plantar nerve | 6 | 6 | 100.00 |  |  |  |  |
| S942 | Injury of deep peroneal nerve at ankle and foot level | 23 | 23 | 100.00 |  |  |  |  |
| S943 | Injury of cutaneous sensory nerve at ankle and foot level | 22 | 22 | 100.00 |  |  |  |  |
| S947 | Injury of multiple nerves at ankle and foot level | 7 | 7 | 100.00 |  |  |  |  |
| S948 | Injury of other nerves at ankle and foot level | 26 | 26 | 100.00 |  |  |  |  |
| S949 | Injury of unspecified nerve at ankle and foot level | 35 | 35 | 100.00 |  |  |  |  |
| S95 | Injury of blood vessels at ankle and foot level | 1420 | 1419 | 99.93 |  |  |  |  |
| S950 | Injury of dorsal artery of foot | 81 | 81 | 100.00 |  |  |  |  |
| S951 | Injury of plantar artery of foot | 86 | 86 | 100.00 |  |  |  |  |
| S952 | Injury of dorsal vein of foot | 97 | 97 | 100.00 |  |  |  |  |
| S957 | Injury of multiple blood vessels at ankle and foot level | 8 | 8 | 100.00 |  |  |  |  |
| S958 | Injury of other blood vessels at ankle and foot level | 595 | 594 | 99.83 |  |  |  |  |
| S959 | Injury of unspecified blood vessel at ankle and foot level | 553 | 553 | 100.00 |  |  |  |  |
| S96 | Injury of muscle and tendon at ankle and foot level | 3892 | 3889 | 99.92 |  |  |  |  |
| S960 | Injury of muscle and tendon of long flexor muscle of toe at ankle and foot level | 428 | 427 | 99.77 |  |  |  |  |
| S961 | Injury of muscle and tendon of long extensor muscle of toe at ankle and foot level | 1187 | 1186 | 99.92 |  |  |  |  |
| S962 | Injury of intrinsic muscle and tendon at ankle and foot level | 263 | 262 | 99.62 |  |  |  |  |
| S967 | Injury of multiple muscles and tendons at ankle and foot level | 163 | 163 | 100.00 |  |  |  |  |
| S968 | Injury of other muscles and tendons at ankle and foot level | 808 | 808 | 100.00 |  |  |  |  |
| S969 | Injury of unspecified muscle tendon at ankle and foot level | 1043 | 1043 | 100.00 |  |  |  |  |
| S97 | Crushing injury of ankle and foot | 1587 | 1584 | 99.81 |  |  |  |  |
| S970 | Crushing injury of ankle | 140 | 140 | 100.00 |  |  |  |  |
| S971 | Crushing injury of toe(s) | 663 | 662 | 99.85 |  |  |  |  |
| S978 | Crushing injury of other parts of ankle and foot | 784 | 782 | 99.74 |  |  |  |  |
| S98 | Traumatic amputation of ankle and foot | 348 | 348 | 100.00 |  |  |  |  |
| S980 | Traumatic amputation of foot at ankle level | 47 | 47 | 100.00 |  |  |  |  |
| S981 | Traumatic amputation of one toe | 179 | 179 | 100.00 |  |  |  |  |
| S982 | Traumatic amputation of two or more toes | 44 | 44 | 100.00 |  |  |  |  |
| S983 | Traumatic amputation of other parts of foot | 44 | 44 | 100.00 |  |  |  |  |
| S984 | Traumatic amputation of foot, level unspecified | 34 | 34 | 100.00 |  |  |  |  |
| S99 | Other and unspecified injuries of ankle and foot | 9536 | 9528 | 99.92 |  |  |  |  |
| S997 | Multiple injuries of ankle and foot | 61 | 61 | 100.00 |  |  |  |  |
| S998 | Other specified injuries of ankle and foot | 657 | 657 | 100.00 |  |  |  |  |
| S999 | Unspecified injury of ankle and foot | 8812 | 8804 | 99.91 |  |  |  |  |
| T00 | Superficial injuries involving multiple body regions | 193832 | 193637 | 99.90 |  |  |  |  |
| T000 | Superficial injuries involving head with neck | 4212 | 4207 | 99.88 |  |  |  |  |
| T001 | Superficial injuries involving thorax with abdomen, lower back and pelvis | 65 | 65 | 100.00 |  |  |  |  |
| T002 | Superficial injuries involving multiple regions of upper limb(s) | 58 | 58 | 100.00 |  |  |  |  |
| T003 | Superficial injuries involving multiple regions of lower limb(s) | 103 | 103 | 100.00 |  |  |  |  |
| T006 | Superficial injuries involving multiple regions of upper limb(s) with lower limb(s) | 67 | 67 | 100.00 |  |  |  |  |
| T008 | Superficial injuries involving other combinations of body regions | 1854 | 1850 | 99.78 |  |  |  |  |
| T009 | Multiple superficial injuries, unspecified | 187473 | 187287 | 99.90 |  |  |  |  |
| T01 | Open wounds involving multiple body regions | 7059 | 7050 | 99.87 |  |  |  |  |
| T010 | Open wounds involving head with neck | 176 | 176 | 100.00 |  |  |  |  |
| T011 | Open wounds involving thorax with abdomen, lower back and pelvis | 31 | 31 | 100.00 |  |  |  |  |
| T012 | Open wounds involving multiple regions of upper limb(s) | 772 | 772 | 100.00 |  |  |  |  |
| T013 | Open wounds involving multiple regions of lower limb(s) | 244 | 244 | 100.00 |  |  |  |  |
| T016 | Open wounds involving multiple regions of upper limb(s) with lower limb(s) | 35 | 35 | 100.00 |  |  |  |  |
| T018 | Open wounds involving other combinations of body regions | 30 | 30 | 100.00 |  |  |  |  |
| T019 | Multiple open wounds, unspecified | 5771 | 5762 | 99.84 |  |  |  |  |
| T02 | Fractures involving multiple body regions | 620 | 619 | 99.84 |  |  |  |  |
| T020 | Fractures involving head with neck | 5 | 5 | 100.00 |  |  |  |  |
| T021 | Fractures involving thorax with lower back and pelvis | 80 | 80 | 100.00 |  |  |  |  |
| T022 | Fractures involving multiple regions of one upper limb | 6 | 6 | 100.00 |  |  |  |  |
| T023 | Fractures involving multiple regions of one lower limb | 16 | 16 | 100.00 |  |  |  |  |
| T024 | Fractures involving multiple regions of both upper limbs | 6 | 6 | 100.00 |  |  |  |  |
| T025 | Fractures involving multiple regions of both lower limbs | 24 | 24 | 100.00 |  |  |  |  |
| T026 | Fractures involving multiple regions of upper limb(s) with lower limb(s) | 7 | 7 | 100.00 |  |  |  |  |
| T027 | Fractures involving thorax with lower back and pelvis with limb(s) | 18 | 18 | 100.00 |  |  |  |  |
| T028 | Fractures involving other combinations of body regions | 159 | 158 | 99.37 |  |  |  |  |
| T029 | Multiple fractures, unspecified | 299 | 299 | 100.00 |  |  |  |  |
| T03 | Dislocations, sprains and strains involving multiple body regions | 3750 | 3745 | 99.87 |  |  |  |  |
| T030 | Dislocations, sprains and strains involving head with neck | 605 | 605 | 100.00 |  |  |  |  |
| T031 | Dislocations, sprains and strains involving thorax with lower back and pelvis | 2076 | 2073 | 99.86 |  |  |  |  |
| T032 | Dislocations, sprains and strains involving multiple regions of upper limb(s) | 112 | 112 | 100.00 |  |  |  |  |
| T033 | Dislocations, sprains and strains involving multiple regions of lower limb(s) | 150 | 149 | 99.33 |  |  |  |  |
| T034 | Dislocations, sprains and strains involving multiple regions of upper limb(s) with lower limb(s) | 15 | 15 | 100.00 |  |  |  |  |
| T038 | Dislocations, sprains and strains involving other combinations of body regions | 307 | 307 | 100.00 |  |  |  |  |
| T039 | Multiple dislocations, sprains and strains, unspecified | 485 | 484 | 99.79 |  |  |  |  |
| T04 | Crushing injuries involving multiple body regions | 200 | 200 | 100.00 |  |  |  |  |
| T040 | Crushing injuries involving head with neck | 3 | 3 | 100.00 |  |  |  |  |
| T041 | Crushing injuries involving thorax with abdomen, lower back and pelvis | 14 | 14 | 100.00 |  |  |  |  |
| T042 | Crushing injuries involving multiple regions of upper limb(s) | 47 | 47 | 100.00 |  |  |  |  |
| T043 | Crushing injuries involving multiple regions of lower limb(s) | 84 | 84 | 100.00 |  |  |  |  |
| T044 | Crushing injuries involving multiple regions of upper limb(s) with lower limb(s) | 6 | 6 | 100.00 |  |  |  |  |
| T047 | Crushing injuries of thorax with abdomen, lower back and pelvis with limb(s) | 3 | 3 | 100.00 |  |  |  |  |
| T048 | Crushing injuries involving other combinations of body regions | 7 | 7 | 100.00 |  |  |  |  |
| T049 | Multiple crushing injuries, unspecified | 36 | 36 | 100.00 |  |  |  |  |
| T05 | Traumatic amputations involving multiple body regions | 62 | 62 | 100.00 |  |  |  |  |
| T050 | Traumatic amputation of both hands | 7 | 7 | 100.00 |  |  |  |  |
| T051 | Traumatic amputation of one hand and other arm [any level, except hand] | 20 | 20 | 100.00 |  |  |  |  |
| T052 | Traumatic amputation of both arm [any level] | 1 | 1 | 100.00 |  |  |  |  |
| T053 | Traumatic amputation of both feet | 1 | 1 | 100.00 |  |  |  |  |
| T054 | Traumatic amputation of one foot and other leg [any level, except foot] | 3 | 3 | 100.00 |  |  |  |  |
| T055 | Traumatic amputation of both legs [any level] | 6 | 6 | 100.00 |  |  |  |  |
| T056 | Traumatic amputation of upper and lower limbs, any combination [any level] | 0 | 0 | 0.00 |  |  |  |  |
| T058 | Traumatic amputation involving other combinations of body regions | 1 | 1 | 100.00 |  |  |  |  |
| T059 | Multiple traumatic amputations, unspecified | 23 | 23 | 100.00 |  |  |  |  |
| T06 | Other injuries involving multiple body regions, NEC | 2857 | 2854 | 99.89 |  |  |  |  |
| T060 | Injuries of brain and cranial nerves with injuries of nerves and spinal cord at neck level | 37 | 37 | 100.00 |  |  |  |  |
| T061 | Injuries of nerves and spinal cord involving other multiple body regions | 86 | 86 | 100.00 |  |  |  |  |
| T062 | Injuries of nerves involving multiple body regions | 7 | 7 | 100.00 |  |  |  |  |
| T063 | Injuries of blood vessels involving multiple body regions | 6 | 6 | 100.00 |  |  |  |  |
| T064 | Injuries of muscles and tendons involving multiple body regions | 25 | 25 | 100.00 |  |  |  |  |
| T065 | Injuries of intrathoracic organs with intra-abdominal and pelvic organs | 18 | 18 | 100.00 |  |  |  |  |
| T068 | Other specified injuries involving multiple body regions | 2678 | 2675 | 99.89 |  |  |  |  |
| T07 | Unspecified multiple injuries | 2803 | 2803 | 100.00 |  |  |  |  |
| T08 | Fracture of spine, level unspecified | 4447 | 4440 | 99.84 |  |  |  |  |
| T080 | Closed | 4432 | 4425 | 99.84 |  |  |  |  |
| T081 | Open | 15 | 15 | 100.00 |  |  |  |  |
| T09 | Other injuries of spine and trunk, level unspecified | 14952 | 14933 | 99.87 |  |  |  |  |
| T090 | Superficial injury of trunk, level unspecified | 1960 | 1959 | 99.95 |  |  |  |  |
| T091 | Open wound of trunk, level unspecified | 583 | 582 | 99.83 |  |  |  |  |
| T092 | Dislocation, sprain and strain of unspecified joint and ligament of trunk | 9823 | 9809 | 99.86 |  |  |  |  |
| T093 | Injury of spinal cord, level unspecified | 2090 | 2088 | 99.90 |  |  |  |  |
| T094 | Injury of unspecified nerve, spinal nerve root and plexus of trunk | 44 | 44 | 100.00 |  |  |  |  |
| T095 | Injury of unspecified muscle and tendon of trunk | 21 | 21 | 100.00 |  |  |  |  |
| T096 | Traumatic amputation of trunk, level unspecified | 0 | 0 | 0.00 |  |  |  |  |
| T098 | Other specified injuries of trunk, level unspecified | 57 | 57 | 100.00 |  |  |  |  |
| T099 | Unspecified injury of trunk, level unspecified | 374 | 373 | 99.73 |  |  |  |  |
| T10 | Fracture of upper limb, level unspecified | 476 | 476 | 100.00 |  |  |  |  |
| T100 | Closed | 446 | 446 | 100.00 |  |  |  |  |
| T101 | Open | 30 | 30 | 100.00 |  |  |  |  |
| T11 | Other injuries of upper limb, level unspecified | 4529 | 4523 | 99.87 |  |  |  |  |
| T110 | Superficial injury of upper limb, level unspecified | 1977 | 1974 | 99.85 |  |  |  |  |
| T111 | Open wound of upper limb, level unspecified | 1139 | 1139 | 100.00 |  |  |  |  |
| T112 | Dislocation, sprain and strain of unspecified joint and ligament of trunk joint and ligament of upper limb, level unspecified | 418 | 418 | 100.00 |  |  |  |  |
| T113 | Injury of unspecified nerve of upper limb, level unspecified | 11 | 11 | 100.00 |  |  |  |  |
| T114 | Injury of unspecified blood vessel of upper limb, level unspecified | 4 | 4 | 100.00 |  |  |  |  |
| T115 | Injury of unspecified muscle and tendon of upper limb, level unspecified | 62 | 62 | 100.00 |  |  |  |  |
| T116 | Traumatic amputation of upper limb, level unspecified | 43 | 43 | 100.00 |  |  |  |  |
| T118 | Other specified injuries of upper limb, level unspecified | 27 | 27 | 100.00 |  |  |  |  |
| T119 | Unspecified injury of upper limb, level unspecified | 848 | 845 | 99.65 |  |  |  |  |
| T12 | Fracture of lower limb, level unspecified | 481 | 481 | 100.00 |  |  |  |  |
| T120 | Closed | 390 | 390 | 100.00 |  |  |  |  |
| T121 | Open | 91 | 91 | 100.00 |  |  |  |  |
| T13 | Other injuries of lower limb, level unspecified | 9592 | 9582 | 99.90 |  |  |  |  |
| T130 | Superficial injury of lower limb, level unspecified | 3450 | 3446 | 99.88 |  |  |  |  |
| T131 | Open wound of lower limb, level unspecified | 3086 | 3083 | 99.90 |  |  |  |  |
| T132 | Dislocation, sprain and strain of unspecified joint and ligament of lower limb, level unspecified | 827 | 826 | 99.88 |  |  |  |  |
| T133 | Injury of unspecified nerve of lower limb, level unspecified | 5 | 5 | 100.00 |  |  |  |  |
| T134 | Injury of unspecified blood vessel of lower limb, level unspecified | 19 | 19 | 100.00 |  |  |  |  |
| T135 | Injury of unspecified muscle and tendon of lower limb, level unspecified | 730 | 729 | 99.86 |  |  |  |  |
| T136 | Traumatic amputation of lower limb, level unspecified | 57 | 57 | 100.00 |  |  |  |  |
| T138 | Other specified injuries of lower limb, level unspecified | 29 | 29 | 100.00 |  |  |  |  |
| T139 | Unspecified injury of lower limb, level unspecified | 1389 | 1388 | 99.93 |  |  |  |  |
| T14 | Injury of unspecified body region | 327971 | 327684 | 99.91 |  |  |  |  |
| T140 | Superficial injury of unspecified body region | 170472 | 170328 | 99.92 |  |  |  |  |
| T141 | Open wound of unspecified body region | 139295 | 139173 | 99.91 |  |  |  |  |
| T142 | Fracture of unspecified body region | 1722 | 1722 | 100.00 |  |  |  |  |
| T143 | Dislocation, sprain and strain of unspecified body region | 3334 | 3329 | 99.85 |  |  |  |  |
| T144 | Injury of nerve(s) of unspecified body region | 83 | 83 | 100.00 |  |  |  |  |
| T145 | Injury of blood vessel(s) of unspecified body region | 283 | 283 | 100.00 |  |  |  |  |
| T146 | Injury of muscles and tendons of unspecified body region | 7409 | 7394 | 99.80 |  |  |  |  |
| T147 | Crushing injury and traumatic amputation of unspecified body region | 712 | 712 | 100.00 |  |  |  |  |
| T148 | Other injuries of unspecified body region | 3677 | 3676 | 99.97 |  |  |  |  |
| T149 | Injury, unspecified | 984 | 984 | 100.00 |  |  |  |  |
| T15 | Foreign body on external eye | 44866 | 44813 | 99.88 |  |  |  |  |
| T150 | Foreign body in cornea | 15329 | 15313 | 99.90 |  |  |  |  |
| T151 | Foreign body in conjunctival sac | 6010 | 6005 | 99.92 |  |  |  |  |
| T158 | Foreign body in other and multiple parts of external eye | 1070 | 1070 | 100.00 |  |  |  |  |
| T159 | Foreign body on external eye, part unspecified | 22457 | 22425 | 99.86 |  |  |  |  |
| T16 | Foreign body in ear | 19886 | 19864 | 99.89 |  |  |  |  |
| T17 | Foreign body in respiratory tract | 106001 | 105891 | 99.90 |  |  |  |  |
| T170 | Foreign body in nasal sinus | 2480 | 2477 | 99.88 |  |  |  |  |
| T171 | Foreign body in nostril | 23964 | 23940 | 99.90 |  |  |  |  |
| T172 | Foreign body in pharynx | 71786 | 71713 | 99.90 |  |  |  |  |
| T173 | Foreign body in larynx | 5774 | 5765 | 99.84 |  |  |  |  |
| T174 | Foreign body in trachea | 327 | 327 | 100.00 |  |  |  |  |
| T175 | Foreign body in bronchus | 413 | 413 | 100.00 |  |  |  |  |
| T178 | Foreign body in other and multiple parts of respiratory tract | 317 | 317 | 100.00 |  |  |  |  |
| T179 | Foreign body in respiratory tract, part unspecified | 940 | 939 | 99.89 |  |  |  |  |
| T18 | Foreign body in alimentary tract | 53223 | 53158 | 99.88 |  |  |  |  |
| T180 | Foreign body in mouth | 4454 | 4448 | 99.87 |  |  |  |  |
| T181 | Foreign body in esophagus | 15997 | 15978 | 99.88 |  |  |  |  |
| T182 | Foreign body in stomach | 6289 | 6281 | 99.87 |  |  |  |  |
| T183 | Foreign body in small intestine | 646 | 646 | 100.00 |  |  |  |  |
| T184 | Foreign body in colon | 352 | 352 | 100.00 |  |  |  |  |
| T185 | Foreign body in anus and rectum | 519 | 518 | 99.81 |  |  |  |  |
| T188 | Foreign body in other and multiple parts of alimentary tract | 537 | 537 | 100.00 |  |  |  |  |
| T189 | Foreign body in alimentary tract, part unspecified | 24429 | 24398 | 99.87 |  |  |  |  |
| T19 | Foreign body in genitourinary tract | 2644 | 2640 | 99.85 |  |  |  |  |
| T190 | Foreign body in urethra | 60 | 58 | 96.67 |  |  |  |  |
| T191 | Foreign body in bladder | 46 | 46 | 100.00 |  |  |  |  |
| T192 | Foreign body in vulva and vagina | 2302 | 2300 | 99.91 |  |  |  |  |
| T193 | Foreign body in uterus [any part] | 35 | 35 | 100.00 |  |  |  |  |
| T198 | Foreign body in other and multiple parts of genitourinary tract | 77 | 77 | 100.00 |  |  |  |  |
| T199 | Foreign body in genitourinary tract, part unspecified | 124 | 124 | 100.00 |  |  |  |  |
| T20 | Burn and corrosion of head and neck | 22123 | 22104 | 99.91 |  |  |  |  |
| T200 | Burn of unspecified degree of head and neck | 3303 | 3298 | 99.85 |  |  |  |  |
| T201 | Burn of first degree of head and neck | 5046 | 5039 | 99.86 |  |  |  |  |
| T202 | Burn of second degree of head and neck | 13376 | 13369 | 99.95 |  |  |  |  |
| T203 | Burn of third degree of head and neck | 171 | 171 | 100.00 |  |  |  |  |
| T204 | Corrosion of unspecified degree of head and neck | 54 | 54 | 100.00 |  |  |  |  |
| T205 | Corrosion of first degree of head and neck | 94 | 94 | 100.00 |  |  |  |  |
| T206 | Corrosion of second degree of head and neck | 73 | 73 | 100.00 |  |  |  |  |
| T207 | Corrosion of third degree of head and neck | 6 | 6 | 100.00 |  |  |  |  |
| T21 | Burn and corrosion of trunk | 15489 | 15473 | 99.90 |  |  |  |  |
| T210 | Burn of unspecified degree of trunk | 1694 | 1691 | 99.82 |  |  |  |  |
| T211 | Burn of first degree of trunk | 2402 | 2399 | 99.88 |  |  |  |  |
| T212 | Burn of second degree of trunk | 11101 | 11091 | 99.91 |  |  |  |  |
| T213 | Burn of third degree of trunk | 234 | 234 | 100.00 |  |  |  |  |
| T214 | Corrosion of unspecified degree of trunk | 20 | 20 | 100.00 |  |  |  |  |
| T215 | Corrosion of first degree of trunk | 19 | 19 | 100.00 |  |  |  |  |
| T216 | Corrosion of second degree of trunk | 19 | 19 | 100.00 |  |  |  |  |
| T217 | Corrosion of third degree of trunk | 0 | 0 | 0.00 |  |  |  |  |
| T22 | Burn and corrosion of shoulder and upper limb, except wrist and hand | 19257 | 19242 | 99.92 |  |  |  |  |
| T220 | Burn of unspecified degree of shoulder and upper limb, except wrist and hand | 2502 | 2499 | 99.88 |  |  |  |  |
| T221 | Burn of first degree of shoulder and upper limb, except wrist and hand | 2298 | 2296 | 99.91 |  |  |  |  |
| T222 | Burn of second degree of shoulder and upper limb, except wrist and hand | 14073 | 14063 | 99.93 |  |  |  |  |
| T223 | Burn of third degree of shoulder and upper limb, except wrist and hand | 238 | 238 | 100.00 |  |  |  |  |
| T224 | Corrosion of unspecified degree of shoulder and upper limb, except wrist and hand | 44 | 44 | 100.00 |  |  |  |  |
| T225 | Corrosion of first degree of shoulder and upper limb, except wrist and hand | 27 | 27 | 100.00 |  |  |  |  |
| T226 | Corrosion of second degree of shoulder and upper limb, except wrist and hand | 65 | 65 | 100.00 |  |  |  |  |
| T227 | Corrosion of third degree of shoulder and upper limb, except wrist and hand | 10 | 10 | 100.00 |  |  |  |  |
| T23 | Burn and corrosion of wrist and hand | 70842 | 70773 | 99.90 |  |  |  |  |
| T230 | Burn of unspecified degree of wrist and hand | 10877 | 10863 | 99.87 |  |  |  |  |
| T231 | Burn of first degree of wrist and hand | 10580 | 10569 | 99.90 |  |  |  |  |
| T232 | Burn of second degree of wrist and hand | 48320 | 48276 | 99.91 |  |  |  |  |
| T233 | Burn of third degree of wrist and hand | 690 | 690 | 100.00 |  |  |  |  |
| T234 | Corrosion of unspecified degree of wrist and hand | 87 | 87 | 100.00 |  |  |  |  |
| T235 | Corrosion of first degree of wrist and hand | 83 | 83 | 100.00 |  |  |  |  |
| T236 | Corrosion of second degree of wrist and hand | 189 | 189 | 100.00 |  |  |  |  |
| T237 | Corrosion of third degree of wrist and hand | 16 | 16 | 100.00 |  |  |  |  |
| T24 | Burn and corrosion of hip and lower limb, except ankle and foot | 34149 | 34123 | 99.92 |  |  |  |  |
| T240 | Burn of unspecified degree of hip and lower limb, except ankle and foot | 3545 | 3543 | 99.94 |  |  |  |  |
| T241 | Burn of first degree of hip and lower limb, except ankle and foot | 4031 | 4029 | 99.95 |  |  |  |  |
| T242 | Burn of second degree of hip and lower limb, except ankle and foot | 25939 | 25917 | 99.92 |  |  |  |  |
| T243 | Burn of third degree of hip and lower limb, except ankle and foot | 433 | 433 | 100.00 |  |  |  |  |
| T244 | Corrosion of unspecified degree of hip and lower limb, except ankle and foot | 30 | 30 | 100.00 |  |  |  |  |
| T245 | Corrosion of first degree of hip and lower imb, except ankle and foot | 31 | 31 | 100.00 |  |  |  |  |
| T246 | Corrosion of second degree of hip and lower limb, except ankle and foot | 122 | 122 | 100.00 |  |  |  |  |
| T247 | Corrosion of third degree of hip and lower limb, except ankle and foot | 18 | 18 | 100.00 |  |  |  |  |
| T25 | Burn and corrosion of ankle and foot | 21404 | 21381 | 99.89 |  |  |  |  |
| T250 | Burn of unspecified degree of ankle and foot | 2669 | 2666 | 99.89 |  |  |  |  |
| T251 | Burn of first degree of ankle and foot | 2021 | 2017 | 99.80 |  |  |  |  |
| T252 | Burn of second degree of ankle and foot | 16335 | 16320 | 99.91 |  |  |  |  |
| T253 | Burn of third degree of ankle and foot | 279 | 278 | 99.64 |  |  |  |  |
| T254 | Corrosion unspecified degree of ankle and foot | 39 | 39 | 100.00 |  |  |  |  |
| T255 | Corrosion of first degree of ankle and foot | 14 | 14 | 100.00 |  |  |  |  |
| T256 | Corrosion of second degree of ankle and foot | 30 | 30 | 100.00 |  |  |  |  |
| T257 | Corrosion of third degree of ankle and foot | 17 | 17 | 100.00 |  |  |  |  |
| T26 | Burn and corrosion confined to eye and adnexa | 7354 | 7345 | 99.88 |  |  |  |  |
| T260 | Burn of eyelid and periocular area | 665 | 664 | 99.85 |  |  |  |  |
| T261 | Burn of cornea and conjunctival sac | 1909 | 1904 | 99.74 |  |  |  |  |
| T262 | Burn with resulting rupture and destruction of eyeball | 9 | 9 | 100.00 |  |  |  |  |
| T263 | Burn of other parts of eye and adnexa | 262 | 262 | 100.00 |  |  |  |  |
| T264 | Burn of eye and adnexa, part unspecified | 604 | 603 | 99.83 |  |  |  |  |
| T265 | Corrosion of eyelid and periocular area | 266 | 266 | 100.00 |  |  |  |  |
| T266 | Corrosion of cornea and conjunctival sac | 3059 | 3058 | 99.97 |  |  |  |  |
| T267 | Corrosion with resulting rupture and destruction of eyeball | 33 | 33 | 100.00 |  |  |  |  |
| T268 | Corrosion of other parts of eye and adnexa | 138 | 138 | 100.00 |  |  |  |  |
| T269 | Corrosion of eye and adnexa, part unspecified | 409 | 408 | 99.76 |  |  |  |  |
| T27 | Burn and corrosion of respiratory tract | 1024 | 1022 | 99.80 |  |  |  |  |
| T270 | Burn of larynx and trachea | 95 | 95 | 100.00 |  |  |  |  |
| T271 | Burn involving larynx and trachea with lung | 55 | 55 | 100.00 |  |  |  |  |
| T272 | Burn of other parts of respiratory tract | 178 | 177 | 99.44 |  |  |  |  |
| T273 | Burn of respiratory tract, part unspecified | 683 | 682 | 99.85 |  |  |  |  |
| T274 | Corrosion of larynx and trachea | 10 | 10 | 100.00 |  |  |  |  |
| T275 | Corrosion involving larynx and trachea with lung | 0 | 0 | 0.00 |  |  |  |  |
| T276 | Corrosion of other parts of respiratory tract | 0 | 0 | 0.00 |  |  |  |  |
| T277 | Corrosion of respiratory tract, part unspecified | 3 | 3 | 100.00 |  |  |  |  |
| T28 | Burn and corrosion of other internal organs | 607 | 607 | 100.00 |  |  |  |  |
| T280 | Burn of mouth and pharynx | 219 | 219 | 100.00 |  |  |  |  |
| T281 | Burn of esophagus | 31 | 31 | 100.00 |  |  |  |  |
| T282 | Burn of other parts of alimentary tract | 8 | 8 | 100.00 |  |  |  |  |
| T283 | Burn of internal genitourinary organs | 46 | 46 | 100.00 |  |  |  |  |
| T284 | Burn of other and unspecified internal organs | 16 | 16 | 100.00 |  |  |  |  |
| T285 | Corrosion of mouth and pharynx | 69 | 69 | 100.00 |  |  |  |  |
| T286 | Corrosion of esophagus | 103 | 103 | 100.00 |  |  |  |  |
| T287 | Corrosion of other parts of alimentary tract | 110 | 110 | 100.00 |  |  |  |  |
| T288 | Corrosion of internal genitourinary organs | 0 | 0 | 0.00 |  |  |  |  |
| T289 | Corrosion of other and unspecified internal organs | 5 | 5 | 100.00 |  |  |  |  |
| T29 | Burns and corrosions of multiple body regions | 1745 | 1742 | 99.83 |  |  |  |  |
| T290 | Burns of multiple regions, unspecified degree | 806 | 803 | 99.63 |  |  |  |  |
| T291 | Burns of multiple regions, no more than first-degree burns mentioned | 164 | 164 | 100.00 |  |  |  |  |
| T292 | Burns of multiple regions, no more than second-degree burns mentioned | 657 | 657 | 100.00 |  |  |  |  |
| T293 | Burns of multiple regions, at least one burn of third degree mentioned | 107 | 107 | 100.00 |  |  |  |  |
| T294 | Corrosions of multiple regions, unspecified degree | 6 | 6 | 100.00 |  |  |  |  |
| T295 | Corrosions of multiple regions, no more than first-degree corrosions mentioned | 2 | 2 | 100.00 |  |  |  |  |
| T296 | Corrosions of multiple regions, no more than second-degree corrosions mentioned | 3 | 3 | 100.00 |  |  |  |  |
| T297 | Corrosions of multiple regions, at least one corrosion of third degree mentioned | 0 | 0 | 0.00 |  |  |  |  |
| T30 | Burn and corrosion, body region unspecified | 62587 | 62510 | 99.88 |  |  |  |  |
| T300 | Burn of unspecified body region, unspecified degree | 37976 | 37932 | 99.88 |  |  |  |  |
| T301 | Burn of first degree, body region unspecified | 3956 | 3950 | 99.85 |  |  |  |  |
| T302 | Burn of second degree, body region unspecified | 18997 | 18973 | 99.87 |  |  |  |  |
| T303 | Burn of third degree, body region unspecified | 186 | 185 | 99.46 |  |  |  |  |
| T304 | Corrosion of unspecified body region, unspecified degree | 1454 | 1452 | 99.86 |  |  |  |  |
| T305 | Corrosion of first degree, body region unspecified | 5 | 5 | 100.00 |  |  |  |  |
| T306 | Corrosion of second degree, body region unspecified | 10 | 10 | 100.00 |  |  |  |  |
| T307 | Corrosion of third degree, body region unspecified | 3 | 3 | 100.00 |  |  |  |  |
| T31 | Burns classified according to extent of body surface involved | 6182 | 6171 | 99.82 |  |  |  |  |
| T310 | Burns involving less than 10％ of body surface | 5281 | 5271 | 99.81 |  |  |  |  |
| T311 | Burns involving 10-19％ of body surface | 406 | 405 | 99.75 |  |  |  |  |
| T312 | Burns involving 20-29％ of body surface | 123 | 123 | 100.00 |  |  |  |  |
| T313 | Burns involving 30-39％ of body surface | 127 | 127 | 100.00 |  |  |  |  |
| T314 | Burns involving 40-49％ of body surface | 61 | 61 | 100.00 |  |  |  |  |
| T315 | Burns involving 50-59％ of body surface | 44 | 44 | 100.00 |  |  |  |  |
| T316 | Burns involving 60-69％ of body surface | 47 | 47 | 100.00 |  |  |  |  |
| T317 | Burns involving 70-79％ of body surface | 29 | 29 | 100.00 |  |  |  |  |
| T318 | Burns involving 80-89％ of body surface | 30 | 30 | 100.00 |  |  |  |  |
| T319 | Burns involving 90％ or more of body surface | 34 | 34 | 100.00 |  |  |  |  |
| T32 | Corrosions classified according to extent of body surface involved | 9 | 9 | 100.00 |  |  |  |  |
| T320 | Corrosions involving less than 10％ of body Corrosions involving 10-19％ of body surface | 6 | 6 | 100.00 |  |  |  |  |
| T321 | Corrosions involving 10-19％ of body surface | 0 | 0 | 0.00 |  |  |  |  |
| T322 | Corrosions involving 20-29％ of body surface | 2 | 2 | 100.00 |  |  |  |  |
| T323 | Corrosions involving 30-39％ of body surface | 0 | 0 | 0.00 |  |  |  |  |
| T324 | Corrosions involving 40-49％ of body surface | 0 | 0 | 0.00 |  |  |  |  |
| T325 | Corrosions involving 50-59％ of body surface | 0 | 0 | 0.00 |  |  |  |  |
| T326 | Corrosions involving 60-69％ of body surface | 1 | 1 | 100.00 |  |  |  |  |
| T327 | Corrosions involving 70-79％ of body surface | 0 | 0 | 0.00 |  |  |  |  |
| T328 | Corrosions involving 80-89％ of body surface | 0 | 0 | 0.00 |  |  |  |  |
| T329 | Corrosions involving 90％ or more of body surface | 0 | 0 | 0.00 |  |  |  |  |
| T33 | Superficial frostbite | 0 | 0 | 0.00 |  |  |  |  |
| T34 | Frostbite with tissue necrosis | 0 | 0 | 0.00 |  |  |  |  |
| T35 | Frostbite involving multiple body regions and unspecified frostbite | 0 | 0 | 0.00 |  |  |  |  |
| T36 | Poisoning by systemic antibiotics | 0 | 0 | 0.00 |  |  |  |  |
| T37 | Poisoning by other systemic anti-infectives and antiparasitic | 0 | 0 | 0.00 |  |  |  |  |
| T38 | Poisoning by hormones and their synthetic substitutes and antagonists, NEC | 0 | 0 | 0.00 |  |  |  |  |
| T39 | Poisoning by nonopioid analgesics, antipyretics and antirheumatics | 0 | 0 | 0.00 |  |  |  |  |
| T40 | Poisoning by narcotics and psychodysleptics [hallucinogens] | 0 | 0 | 0.00 |  |  |  |  |
| T41 | Poisoning by anesthetics and therapeutic gases | 0 | 0 | 0.00 |  |  |  |  |
| T42 | Poisoning by antiepileptic, sedative-hypnotic and antiparkinsonism drugs | 0 | 0 | 0.00 |  |  |  |  |
| T43 | Poisoning by psychotropic drugs, NEC | 0 | 0 | 0.00 |  |  |  |  |
| T44 | Poisoning by drugs primarily affecting the autonomic nervous system | 0 | 0 | 0.00 |  |  |  |  |
| T45 | Poisoning by primarily systemic and hematological agents, NEC | 0 | 0 | 0.00 |  |  |  |  |
| T46 | Poisoning by agents primarily affecting the cardiovascular system | 0 | 0 | 0.00 |  |  |  |  |
| T47 | Poisoning by agents primarily affecting the gastrointestinal system | 0 | 0 | 0.00 |  |  |  |  |
| T48 | Poisoning by agents primarily acting on smooth and skeletal muscles and the respiratory system | 0 | 0 | 0.00 |  |  |  |  |
| T49 | Poisoning by topical agents primarily affecting skin and mucous membrane and by ophthalmological, otorhinolaryngological and dental drugs | 0 | 0 | 0.00 |  |  |  |  |
| T50 | Poisoning by diuretics and other and unspecified drugs, medicaments and biological substances | 0 | 0 | 0.00 |  |  |  |  |
| T51 | Toxic effect of alcohol | 0 | 0 | 0.00 |  |  |  |  |
| T52 | Toxic effect of organic solvents | 0 | 0 | 0.00 |  |  |  |  |
| T53 | Toxic effect of halogen derivatives of aliphatic and aromatic hydrocarbons | 0 | 0 | 0.00 |  |  |  |  |
| T54 | Toxic effect of corrosive substances | 0 | 0 | 0.00 |  |  |  |  |
| T55 | Toxic effect of soaps and detergents | 0 | 0 | 0.00 |  |  |  |  |
| T56 | Toxic effect of metals | 0 | 0 | 0.00 |  |  |  |  |
| T57 | Toxic effect of other inorganic substances | 0 | 0 | 0.00 |  |  |  |  |
| T58 | Toxic effect of carbon monoxide | 0 | 0 | 0.00 |  |  |  |  |
| T59 | Toxic effect of other gases, fumes and vapours | 0 | 0 | 0.00 |  |  |  |  |
| T60 | Toxic effect of pesticides | 0 | 0 | 0.00 |  |  |  |  |
| T61 | Toxic effect of noxious substances eaten as seafood | 0 | 0 | 0.00 |  |  |  |  |
| T62 | Toxic effect of other noxious substances eaten as food | 0 | 0 | 0.00 |  |  |  |  |
| T63 | Toxic effect of contact with venomous animals | 0 | 0 | 0.00 |  |  |  |  |
| T64 | Toxic effect of aflatoxin and other mycotoxin food contaminants | 0 | 0 | 0.00 |  |  |  |  |
| T65 | Toxic effect of other and unspecified substances | 0 | 0 | 0.00 |  |  |  |  |
| T66 | Unspecified effects of radiation | 0 | 0 | 0.00 |  |  |  |  |
| T67 | Effects of heat and light | 0 | 0 | 0.00 |  |  |  |  |
| T68 | Hypothermia | 0 | 0 | 0.00 |  |  |  |  |
| T69 | Other effects of reduced temperature | 0 | 0 | 0.00 |  |  |  |  |
| T70 | Effects of air pressure and water pressure | 0 | 0 | 0.00 |  |  |  |  |
| T71 | Asphyxiation | 0 | 0 | 0.00 |  |  |  |  |
| T73 | Effects of other deprivation | 0 | 0 | 0.00 |  |  |  |  |
| T74 | Maltreatment syndromes | 0 | 0 | 0.00 |  |  |  |  |
| T75 | Effects of other external causes | 0 | 0 | 0.00 |  |  |  |  |
| T76 | Unspecified effects of external causes | 0 | 0 | 0.00 |  |  |  |  |
| T77 |  | 0 | 0 | 0.00 |  |  |  |  |
| T78 | Adverse effects, NEC | 0 | 0 | 0.00 |  |  |  |  |
| T79 | Certain early complications of trauma, NEC | 4099 | 4092 | 99.83 |  |  |  |  |
| T790 | Air embolism (traumatic) | 7 | 7 | 100.00 |  |  |  |  |
| T791 | Fat embolism (traumatic) | 23 | 23 | 100.00 |  |  |  |  |
| T792 | Traumatic secondary and recurrent hemorrhage | 45 | 45 | 100.00 |  |  |  |  |
| T793 | Post-traumatic wound infection, NEC | 906 | 905 | 99.89 |  |  |  |  |
| T794 | Traumatic shock | 1085 | 1082 | 99.72 |  |  |  |  |
| T795 | Traumatic anuria | 4 | 4 | 100.00 |  |  |  |  |
| T796 | Traumatic ischemia of muscle | 1049 | 1047 | 99.81 |  |  |  |  |
| T797 | Traumatic subcutaneous emphysema | 935 | 934 | 99.89 |  |  |  |  |
| T798 | Other early complications of trauma | 29 | 29 | 100.00 |  |  |  |  |
| T799 | Unspecified early complication of trauma | 16 | 16 | 100.00 |  |  |  |  |
| T80 | Complications following infusion, transfusion and therapeutic injection | 0 | 0 | 0.00 |  |  |  |  |
| T81 | Complications of procedures, NEC | 0 | 0 | 0.00 |  |  |  |  |
| T82 | Complications of cardiac and vascular prosthetic devices, implants and grafts | 0 | 0 | 0.00 |  |  |  |  |
| T83 | Complications of genitourinary prosthetic devices, implants and grafts | 0 | 0 | 0.00 |  |  |  |  |
| T84 | Complications of internal orthopedic prosthetic devices, implants and grafts | 0 | 0 | 0.00 |  |  |  |  |
| T85 | Complications of other internal prosthetic devices, implants and grafts | 0 | 0 | 0.00 |  |  |  |  |
| T86 | Failure and rejection of transplanted organs and tissues | 0 | 0 | 0.00 |  |  |  |  |
| T87 | Complications peculiar to reattachment and amputation | 0 | 0 | 0.00 |  |  |  |  |
| T88 | Other complications of surgical and medical care, NEC | 0 | 0 | 0.00 |  |  |  |  |
| T90 | Sequelae of injuries of head | 390 | 390 | 100.00 |  |  |  |  |
| T900 | Sequelae of superficial injury of head | 69 | 69 | 100.00 |  |  |  |  |
| T901 | Sequelae of open wound of head | 50 | 50 | 100.00 |  |  |  |  |
| T902 | Sequelae of fracture of skull and facial bones | 130 | 130 | 100.00 |  |  |  |  |
| T903 | Sequelae of injury of cranial nerves | 1 | 1 | 100.00 |  |  |  |  |
| T904 | Sequelae of injury of eye and orbit | 32 | 32 | 100.00 |  |  |  |  |
| T905 | Sequelae of intracranial injury | 69 | 69 | 100.00 |  |  |  |  |
| T908 | Sequelae of other specified injuries of head | 5 | 5 | 100.00 |  |  |  |  |
| T909 | Sequelae of unspecified injury of head | 34 | 34 | 100.00 |  |  |  |  |
| T91 | Sequelae of injuries of neck and trunk | 411 | 409 | 99.51 |  |  |  |  |
| T910 | Sequelae of superficial injury and open wound of neck and trunk | 41 | 40 | 97.56 |  |  |  |  |
| T911 | Sequelae of fracture of spine | 222 | 221 | 99.55 |  |  |  |  |
| T912 | Sequelae of other fracture of thorax and pelvis | 78 | 78 | 100.00 |  |  |  |  |
| T913 | Sequelae of injury of spinal cord | 66 | 66 | 100.00 |  |  |  |  |
| T914 | Sequelae of injury of intrathoracic organs | 2 | 2 | 100.00 |  |  |  |  |
| T915 | Sequelae of injury of intra-abdominal and pelvic organs | 2 | 2 | 100.00 |  |  |  |  |
| T918 | Sequelae of other specified injuries of neck and trunk | 0 | 0 | 0.00 |  |  |  |  |
| T919 | Sequelae of unspecified injury of neck and trunk | 0 | 0 | 0.00 |  |  |  |  |
| T92 | Sequelae of injuries of upper limb | 114 | 113 | 99.12 |  |  |  |  |
| T920 | Sequelae of open wound of upper limb | 8 | 8 | 100.00 |  |  |  |  |
| T921 | Sequelae of fracture of arm | 15 | 15 | 100.00 |  |  |  |  |
| T922 | Sequelae of fracture at wrist and hand level | 21 | 21 | 100.00 |  |  |  |  |
| T923 | Sequelae of dislocation, sprain and strain of upper limb | 50 | 50 | 100.00 |  |  |  |  |
| T924 | Sequelae of injury of nerve of upper limb | 0 | 0 | 0.00 |  |  |  |  |
| T925 | Sequelae of injury of muscle and tendon of upper limb | 13 | 13 | 100.00 |  |  |  |  |
| T926 | Sequelae of crushing injury and traumatic amputation of upper limb | 6 | 5 | 83.33 |  |  |  |  |
| T928 | Sequelae of other specified injuries of upper limb | 0 | 0 | 0.00 |  |  |  |  |
| T929 | Sequelae of unspecified injury of upper limb | 1 | 1 | 100.00 |  |  |  |  |
| T93 | Sequelae of injuries of lower limb | 179 | 179 | 100.00 |  |  |  |  |
| T930 | Sequelae of open wound of lower limb | 33 | 33 | 100.00 |  |  |  |  |
| T931 | Sequelae of fracture of femur | 23 | 23 | 100.00 |  |  |  |  |
| T932 | Sequelae of other fractures of lower limb | 17 | 17 | 100.00 |  |  |  |  |
| T933 | Sequelae of dislocation, sprain and strain of lower limb | 74 | 74 | 100.00 |  |  |  |  |
| T934 | Sequelae of injury of nerve of lower limb | 6 | 6 | 100.00 |  |  |  |  |
| T935 | Sequelae of injury of muscle and tendon of lower limb | 15 | 15 | 100.00 |  |  |  |  |
| T936 | Sequelae of crushing injury and traumatic amputation of lower limb | 7 | 7 | 100.00 |  |  |  |  |
| T938 | Sequelae of other specified injuries of lower limb | 1 | 1 | 100.00 |  |  |  |  |
| T939 | Sequelae of unspecified injury of lower limb | 3 | 3 | 100.00 |  |  |  |  |
| T94 | Sequelae of injuries involving multiple and unspecified body regions | 6 | 6 | 100.00 |  |  |  |  |
| T940 | Sequelae of injuries involving multiple body regions | 1 | 1 | 100.00 |  |  |  |  |
| T941 | Sequelae of injuries, not specified by body region | 5 | 5 | 100.00 |  |  |  |  |
| T95 | Sequelae of burns, corrosions and frostbite | 188 | 188 | 100.00 |  |  |  |  |
| T950 | Sequelae of burn, corrosion and frostbite of head and neck | 28 | 28 | 100.00 |  |  |  |  |
| T951 | Sequelae of burn, corrosion and frostbite of trunk | 7 | 7 | 100.00 |  |  |  |  |
| T952 | Sequelae of burn, corrosion and frostbite of upper limb | 80 | 80 | 100.00 |  |  |  |  |
| T953 | Sequelae of burn, corrosion and frostbite of lower limb | 29 | 29 | 100.00 |  |  |  |  |
| T954 | Sequelae of burn and corrosion classifiable only according to extent of body surface involved | 2 | 2 | 100.00 |  |  |  |  |
| T958 | Sequelae of other specified burn, corrosion and frostbite | 9 | 9 | 100.00 |  |  |  |  |
| T959 | Sequelae of unspecified burn, corrosion and frostbite | 33 | 33 | 100.00 |  |  |  |  |
| T96 | Sequelae of poisoning by drugs, medicaments and biological substances | 80 | 80 | 100.00 |  |  |  |  |
| T97 | Sequelae of toxic effects of substances chiefly nonmedicinal as to source | 87 | 86 | 98.85 |  |  |  |  |
| T98 | Sequelae of other and unspecified effects of external causes | 66 | 66 | 100.00 |  |  |  |  |
| T980 | Sequelae of effects of foreign body entering through natural orifice | 48 | 48 | 100.00 |  |  |  |  |
| T981 | Sequelae of other and unspecified effects of external causes | 14 | 14 | 100.00 |  |  |  |  |
| T982 | Sequelae of certain early complications of trauma | 1 | 1 | 100.00 |  |  |  |  |
| T983 | Sequelae of complications of surgical and medical care, NEC | 3 | 3 | 100.00 |  |  |  |  |
| **Exclusive SRR** | | | | |  | 3 | 3 | 100 |
| **Code** | **Name** | **Total (N)** | **Survivor**  **(N)** | **Exclusive**  **SRR (%)** |  |  |  |  |
| S00 | superficial injury of head | 229018 | 228953 | 99.97162 |  |  |  |  |
| S001 | Contusion of eyelid and periocular area | 13843 | 13843 | 100 |  |  |  |  |
| S002 | Other superficial injuries of eyelid and periocular area | 6701 | 6701 | 100 |  |  |  |  |
| S003 | Superficial injury of nose | 19949 | 19949 | 100 |  |  |  |  |
| S004 | Superficial injury of ear | 7012 | 7012 | 100 |  |  |  |  |
| S005 | Superficial injury of lip and oral cavity | 13994 | 13994 | 100 |  |  |  |  |
| S007 | Multiple superficial injuries of head | 882 | 882 | 100 |  |  |  |  |
| S008 | Superficial injury of other parts of head | 136237 | 136218 | 99.98605 |  |  |  |  |
| S009 | Superficial injury of head, part unspecified | 57407 | 57389 | 99.96864 |  |  |  |  |
| S01 | open wound of head | 36383 | 36040 | 99.05725 |  |  |  |  |
| S010 | Open wound of scalp | 179422 | 179395 | 99.98495 |  |  |  |  |
| S011 | Open wound of eyelid and periocular area | 96653 | 96652 | 99.99897 |  |  |  |  |
| S012 | Open wound of nose | 10590 | 10590 | 100 |  |  |  |  |
| S013 | Open wound of ear | 19233 | 19233 | 100 |  |  |  |  |
| S014 | Open wound of cheek and temporomandibular area | 30922 | 30922 | 100 |  |  |  |  |
| S015 | Open wound of lip | 84057 | 84056 | 99.99881 |  |  |  |  |
| S017 | Multiple open wounds of head | 1772 | 1768 | 99.77427 |  |  |  |  |
| S018 | Open wound of other parts of head | 267191 | 267166 | 99.99064 |  |  |  |  |
| S019 | Open wound of head, part unspecified | 27909 | 27903 | 99.9785 |  |  |  |  |
| S02 | fracture of skull and facial bones | 2791 | 2783 | 99.71336 |  |  |  |  |
| S020 | Fracture of vault of skull | 1472 | 1410 | 95.78804 |  |  |  |  |
| S021 | Fracture of base of skull | 1325 | 1282 | 96.75472 |  |  |  |  |
| S022 | Fracture of nasal bones | 24158 | 24157 | 99.99586 |  |  |  |  |
| S023 | Fracture of orbital floor | 4097 | 4097 | 100 |  |  |  |  |
| S024 | Fracture of malar and maxillary bones | 3629 | 3628 | 99.97244 |  |  |  |  |
| S025 | Fracture of tooth | 6857 | 6857 | 100 |  |  |  |  |
| S026 | Fracture of mandible | 4002 | 3999 | 99.92504 |  |  |  |  |
| S027 | Multiple fractures involving skull and facial bones | 134 | 124 | 92.53731 |  |  |  |  |
| S028 | Fractures of other skull and facial bones | 4017 | 4002 | 99.62659 |  |  |  |  |
| S029 | Fracture of skull and facial bones, part unspecified | 2701 | 2620 | 97.00111 |  |  |  |  |
| S03 | Dislocation, sprain and strain of joints and ligaments of head | 35783 | 35732 | 99.85747 |  |  |  |  |
| S030 | Dislocation of jaw | 7501 | 7501 | 100 |  |  |  |  |
| S031 | Dislocation of septal cartilage of nose | 1 | 1 | 100 |  |  |  |  |
| S032 | Dislocation of tooth | 7422 | 7422 | 100 |  |  |  |  |
| S033 | Dislocation of other and unspecified parts of head | 4 | 4 | 100 |  |  |  |  |
| S034 | Sprain and strain of jaw | 513 | 513 | 100 |  |  |  |  |
| S035 | Sprain and strain of joints and ligaments of other and unspecified parts of head | 143 | 143 | 100 |  |  |  |  |
| S04 | Injury of cranial nerve | 13562 | 13528 | 99.7493 |  |  |  |  |
| S040 | Injury of optic nerve and pathways | 66 | 66 | 100 |  |  |  |  |
| S041 | Injury of oculomotor nerve | 7 | 7 | 100 |  |  |  |  |
| S042 | Injury of trochlear nerve | 4 | 4 | 100 |  |  |  |  |
| S043 | Injury of trigeminal nerve | 8 | 8 | 100 |  |  |  |  |
| S044 | Injury of abducent nerve | 1 | 1 | 100 |  |  |  |  |
| S045 | Injury of facial nerve | 34 | 34 | 100 |  |  |  |  |
| S046 | Injury of acoustic nerve | 11 | 11 | 100 |  |  |  |  |
| S047 | Injury of accessory nerve | 0 | 0 | 0 |  |  |  |  |
| S048 | Injury of other cranial nerves | 1 | 1 | 100 |  |  |  |  |
| S049 | Injury of unspecified cranial nerve | 0 | 0 | 0 |  |  |  |  |
| S05 | Injury of eye and orbit | 3243 | 3212 | 99.04409 |  |  |  |  |
| S050 | Injury of conjunctiva and corneal abrasion without mention of foreign body | 40936 | 40936 | 100 |  |  |  |  |
| S051 | Contusion of eyeball and orbital tissues | 12941 | 12941 | 100 |  |  |  |  |
| S052 | Ocular laceration and rupture with prolapse or loss of intraocular tissue | 218 | 218 | 100 |  |  |  |  |
| S053 | Ocular laceration without prolapse or loss of intraocular tissue | 1376 | 1376 | 100 |  |  |  |  |
| S054 | Penetrating wound of orbit with or without foreign body | 106 | 106 | 100 |  |  |  |  |
| S055 | Penetrating wound of eyeball with foreign body | 427 | 427 | 100 |  |  |  |  |
| S056 | Penetrating wound of eyeball without foreign body | 311 | 311 | 100 |  |  |  |  |
| S057 | Avulsion of eye | 10 | 10 | 100 |  |  |  |  |
| S058 | Other injuries of eye and orbit | 3209 | 3209 | 100 |  |  |  |  |
| S059 | Injury of eye and orbit, unspecified | 6577 | 6577 | 100 |  |  |  |  |
| S06 | Intracranial injury | 111065 | 111040 | 99.97749 |  |  |  |  |
| S060 | Concussion | 216784 | 216719 | 99.97002 |  |  |  |  |
| S061 | Traumatic cerebral oedema | 30 | 28 | 93.33333 |  |  |  |  |
| S062 | Diffuse brain injury | 5435 | 5405 | 99.44802 |  |  |  |  |
| S063 | Focal brain injury | 1426 | 1423 | 99.78962 |  |  |  |  |
| S064 | Epidural haemorrhage | 2651 | 2644 | 99.73595 |  |  |  |  |
| S065 | Traumatic subdural haemorrhage | 20375 | 20286 | 99.56319 |  |  |  |  |
| S066 | Traumatic subarachnoid haemorrhage | 4263 | 4218 | 98.94441 |  |  |  |  |
| S067 | Intracranial injury with prolonged coma | 4 | 3 | 75 |  |  |  |  |
| S068 | Other intracranial injuries | 1798 | 1766 | 98.22024 |  |  |  |  |
| S069 | Intracranial injury, unspecified | 401 | 267 | 66.58354 |  |  |  |  |
| S07 | Crushing injury of head | 11 | 11 | 100 |  |  |  |  |
| S070 | Crushing injury of face | 181 | 179 | 98.89503 |  |  |  |  |
| S071 | Crushing injury of skull | 8 | 6 | 75 |  |  |  |  |
| S078 | Crushing injury of other parts of head | 25 | 23 | 92 |  |  |  |  |
| S079 | Crushing injury of head, part unspecified | 32 | 30 | 93.75 |  |  |  |  |
| S08 | Traumatic amputation of part of head | 172 | 168 | 97.67442 |  |  |  |  |
| S080 | Avulsion of scalp | 562 | 562 | 100 |  |  |  |  |
| S081 | Traumatic amputation of ear | 31 | 31 | 100 |  |  |  |  |
| S088 | Traumatic amputation of other parts of head | 4 | 4 | 100 |  |  |  |  |
| S089 | Traumatic amputation of unspecified part of head | 3 | 3 | 100 |  |  |  |  |
| S09 | Other and unspecified injuries of head | 236 | 233 | 98.72881 |  |  |  |  |
| S090 | Injury of blood vessels of head, NEC | 8 | 8 | 100 |  |  |  |  |
| S091 | Injury of muscle and tendon of head | 1937 | 1937 | 100 |  |  |  |  |
| S092 | Traumatic rupture of ear drum | 2146 | 2146 | 100 |  |  |  |  |
| S097 | Multiple injuries of head | 20 | 12 | 60 |  |  |  |  |
| S098 | Other specified injuries of head | 334 | 331 | 99.1018 |  |  |  |  |
| S099 | Unspecified injury of head | 30763 | 30725 | 99.87647 |  |  |  |  |
| S10 | Superficial injury of neck | 74713 | 74687 | 99.9652 |  |  |  |  |
| S100 | Contusion of throat | 439 | 438 | 99.77221 |  |  |  |  |
| S101 | Other and unspecified superficial injuries of throat | 2081 | 2080 | 99.95195 |  |  |  |  |
| S107 | Multiple superficial injuries of neck | 71 | 71 | 100 |  |  |  |  |
| S108 | Superficial injury of other parts of neck | 4174 | 4166 | 99.80834 |  |  |  |  |
| S109 | Superficial injury of neck, part unspecified | 6816 | 6804 | 99.82394 |  |  |  |  |
| S11 | Open wound of neck | 5250 | 5245 | 99.90476 |  |  |  |  |
| S111 | Open wound involving thyroid gland | 2 | 2 | 100 |  |  |  |  |
| S112 | Open wound involving pharynx and cervical esophagus | 58 | 58 | 100 |  |  |  |  |
| S117 | Multiple open wounds of neck | 105 | 104 | 99.04762 |  |  |  |  |
| S118 | Open wound of other parts of neck | 706 | 705 | 99.85836 |  |  |  |  |
| S119 | Open wound of neck, part unspecified | 1585 | 1576 | 99.43218 |  |  |  |  |
| S12 | Fracture of neck | 16681 | 16665 | 99.90408 |  |  |  |  |
| S120 | Fracture of first cervical vertebra | 121 | 120 | 99.17355 |  |  |  |  |
| S121 | Fracture of second cervical vertebra | 423 | 420 | 99.29078 |  |  |  |  |
| S122 | Fracture of other specified cervical vertebra | 488 | 487 | 99.79508 |  |  |  |  |
| S127 | Multiple fractures of cervical spine | 189 | 188 | 99.4709 |  |  |  |  |
| S128 | Fracture of other parts of neck | 93 | 91 | 97.84946 |  |  |  |  |
| S129 | Fracture of neck, part unspecified | 1598 | 1586 | 99.24906 |  |  |  |  |
| S13 | Dislocation, sprain and strain of joints and ligaments at neck level | 41806 | 41627 | 99.57183 |  |  |  |  |
| S130 | Traumatic rupture of cervical intervertebra disc | 78 | 77 | 98.71795 |  |  |  |  |
| S131 | Dislocation of cervical vertebra | 245 | 235 | 95.91837 |  |  |  |  |
| S132 | Dislocation of other and unspecified parts of neck | 5 | 5 | 100 |  |  |  |  |
| S133 | Multiple dislocations of neck | 3 | 3 | 100 |  |  |  |  |
| S134 | Sprain and strain of cervical spine | 98856 | 98839 | 99.9828 |  |  |  |  |
| S135 | Sprain and strain of thyroid region | 12 | 12 | 100 |  |  |  |  |
| S136 | Sprain and strain of joints and ligaments of other and unspecified parts of neck | 10362 | 10361 | 99.99035 |  |  |  |  |
| S14 | Injury of nerves and spinal cord at neck level | 9900 | 9900 | 100 |  |  |  |  |
| S140 | Concussion and oedema of cervical spinal cord | 147 | 147 | 100 |  |  |  |  |
| S141 | Other and unspecified injuries of cervical spinal cord | 1510 | 1503 | 99.53642 |  |  |  |  |
| S142 | Injury of nerve root of cervical spine | 73 | 73 | 100 |  |  |  |  |
| S143 | Injury of brachial plexus | 53 | 53 | 100 |  |  |  |  |
| S144 | Injury of peripheral nerves of neck | 1 | 1 | 100 |  |  |  |  |
| S145 | Injury of cervical sympathetic nerves | 0 | 0 | 0 |  |  |  |  |
| S146 | Injury of other and unspecified nerves of neck | 41 | 41 | 100 |  |  |  |  |
| S15 | Injury of blood vessels at neck level | 83 | 69 | 83.13253 |  |  |  |  |
| S150 | Injury of carotid artery | 10 | 10 | 100 |  |  |  |  |
| S151 | Injury of vertebral artery | 4 | 4 | 100 |  |  |  |  |
| S152 | Injury of external jugular vein | 1 | 1 | 100 |  |  |  |  |
| S153 | Injury of internal jugular vein | 2 | 2 | 100 |  |  |  |  |
| S157 | Injury of multiple blood vessels at neck level | 4 | 4 | 100 |  |  |  |  |
| S158 | Injury of other blood vessels at neck level | 50 | 50 | 100 |  |  |  |  |
| S159 | Injury of unspecified blood vessels at neck level | 72 | 69 | 95.83333 |  |  |  |  |
| S16 | Injury of muscle and tendon at neck level | 2 | 2 | 100 |  |  |  |  |
| S17 | Crushing injury of neck | 6 | 4 | 66.66667 |  |  |  |  |
| S170 | Crushing injury of larynx and trachea | 5 | 4 | 80 |  |  |  |  |
| S178 | Crushing injury of other parts of neck | 0 | 0 | 0 |  |  |  |  |
| S179 | Crushing injury of neck, part unspecified | 10 | 9 | 90 |  |  |  |  |
| S18 | Traumatic amputation at neck level | 19 | 18 | 94.73684 |  |  |  |  |
| S19 | Other and unspecified injuries of neck | 119 | 92 | 77.31092 |  |  |  |  |
| S197 | Multiple injuries of neck | 8 | 8 | 100 |  |  |  |  |
| S198 | Other specified injuries of neck | 214 | 212 | 99.06542 |  |  |  |  |
| S199 | Unspecified injury of neck | 649 | 639 | 98.45917 |  |  |  |  |
| S20 | Superficial injury of thorax | 2509 | 2216 | 88.32204 |  |  |  |  |
| S200 | Contusion of breast | 84 | 84 | 100 |  |  |  |  |
| S201 | Other and unspecified superficial injuries of breast | 61 | 61 | 100 |  |  |  |  |
| S202 | Contusion of thorax | 73320 | 73306 | 99.98091 |  |  |  |  |
| S203 | Other superficial injuries of front wall of thorax | 599 | 597 | 99.66611 |  |  |  |  |
| S204 | Other superficial injuries of back wall of thorax | 528 | 528 | 100 |  |  |  |  |
| S207 | Multiple superficial injuries of thorax | 30 | 30 | 100 |  |  |  |  |
| S208 | Superficial injury of other and unspecified parts of thorax | 3104 | 3104 | 100 |  |  |  |  |
| S21 | Open wound of thorax | 184 | 171 | 92.93478 |  |  |  |  |
| S210 | Open wound of breast | 77 | 77 | 100 |  |  |  |  |
| S211 | Open wound of front wall of thorax | 694 | 689 | 99.27954 |  |  |  |  |
| S212 | Open wound of back wall of thorax | 817 | 817 | 100 |  |  |  |  |
| S217 | Multiple open wounds of thoracic wall | 14 | 14 | 100 |  |  |  |  |
| S218 | Open wound of other parts of thorax | 148 | 147 | 99.32432 |  |  |  |  |
| S219 | Open wound of thorax, part unspecified | 652 | 642 | 98.46626 |  |  |  |  |
| S22 | Fracture of rib(s), sternum and thoracic spine | 59992 | 59951 | 99.93166 |  |  |  |  |
| S220 | Fracture of thoracic vertebra | 12354 | 12353 | 99.99191 |  |  |  |  |
| S221 | Multiple fractures of thoracic spine | 355 | 353 | 99.43662 |  |  |  |  |
| S222 | Fracture of sternum | 3158 | 3156 | 99.93667 |  |  |  |  |
| S223 | Fracture of rib | 19385 | 19370 | 99.92262 |  |  |  |  |
| S224 | Multiple fracture of ribs | 22137 | 21984 | 99.30885 |  |  |  |  |
| S225 | Flail chest | 39 | 29 | 74.35897 |  |  |  |  |
| S228 | Fracture of other parts of bony thorax | 125 | 125 | 100 |  |  |  |  |
| S229 | Fracture of bony thorax, part unspecified | 99 | 98 | 98.9899 |  |  |  |  |
| S23 | Dislocation, sprain and strain of joints and ligaments of thorax | 10521 | 10511 | 99.90495 |  |  |  |  |
| S230 | Traumatic rupture of thoracic intervertebral disc | 8 | 8 | 100 |  |  |  |  |
| S231 | Dislocation of thoracic vertebra | 13 | 13 | 100 |  |  |  |  |
| S232 | Dislocation of other and unspecified parts of thorax | 4 | 4 | 100 |  |  |  |  |
| S233 | Sprain and strain of thoracic spine | 2915 | 2915 | 100 |  |  |  |  |
| S234 | Sprain and strain of ribs and sternum | 4733 | 4733 | 100 |  |  |  |  |
| S235 | Sprain and strain of other and unspecified parts of thorax | 2196 | 2196 | 100 |  |  |  |  |
| S24 | Injury of nerves and spinal cord at thorax level | 34665 | 34653 | 99.96538 |  |  |  |  |
| S240 | Concussion and oedema of thoracic spinal cord | 7 | 7 | 100 |  |  |  |  |
| S241 | Other and unspecified injuries of thoracic spinal cord | 45 | 45 | 100 |  |  |  |  |
| S242 | Injury of nerve root of thoracic spine | 14 | 14 | 100 |  |  |  |  |
| S243 | Injury of peripheral nerves of thorax | 0 | 0 | 0 |  |  |  |  |
| S244 | Injury of thoracic sympathetic nerves | 3 | 3 | 100 |  |  |  |  |
| S245 | Injury of other nerves of thorax | 0 | 0 | 0 |  |  |  |  |
| S246 | Injury of unspecified nerve of thorax | 4 | 4 | 100 |  |  |  |  |
| S25 | Injury of blood vessels of thorax | 10541 | 10442 | 99.06081 |  |  |  |  |
| S250 | Injury of thoracic aorta | 60 | 46 | 76.66667 |  |  |  |  |
| S251 | Injury of innominate or subclavian artery | 2 | 2 | 100 |  |  |  |  |
| S252 | Injury of superior vena cava | 0 | 0 | 0 |  |  |  |  |
| S253 | Injury of innominate or subclavian vein | 2 | 2 | 100 |  |  |  |  |
| S254 | Injury of pulmonary blood vessels | 2 | 0 | 0 |  |  |  |  |
| S255 | Injury of intercostal blood vessels | 4 | 4 | 100 |  |  |  |  |
| S257 | Injury of multiple blood vessels of thorax | 0 | 0 | 0 |  |  |  |  |
| S258 | Injury of other blood vessels of thorax | 19 | 18 | 94.73684 |  |  |  |  |
| S259 | Injury of unspecified blood vessel of thorax | 19 | 19 | 100 |  |  |  |  |
| S26 | Injury of heart | 103583 | 103581 | 99.99807 |  |  |  |  |
| S260 | Injury of heart with hemopericardium | 46 | 31 | 67.3913 |  |  |  |  |
| S268 | Other injuries of heart | 25 | 20 | 80 |  |  |  |  |
| S269 | Injury of heart, unspecified | 29 | 22 | 75.86207 |  |  |  |  |
| S27 | Injury of other and unspecified intrathoracic organs | 6997 | 6996 | 99.98571 |  |  |  |  |
| S270 | Traumatic pneumothorax | 958 | 916 | 95.61587 |  |  |  |  |
| S271 | Traumatic hemothorax | 654 | 542 | 82.87462 |  |  |  |  |
| S272 | Traumatic hemopneumothorax | 593 | 473 | 79.76391 |  |  |  |  |
| S273 | Other injuries of lung | 228 | 217 | 95.17544 |  |  |  |  |
| S274 | Injury of bronchus | 5 | 4 | 80 |  |  |  |  |
| S275 | Injury of thoracic trachea | 65 | 58 | 89.23077 |  |  |  |  |
| S276 | Injury of pleura | 6 | 6 | 100 |  |  |  |  |
| S277 | Multiple injuries of intrathoracic organs | 4 | 1 | 25 |  |  |  |  |
| S278 | Injury of other specified intrathoracic organs | 103 | 101 | 98.05825 |  |  |  |  |
| S279 | Injury of unspecified intrathoracic organ | 18 | 14 | 77.77778 |  |  |  |  |
| S28 | Crushing injury of thorax and traumatic amputation of part of thorax | 125 | 120 | 96 |  |  |  |  |
| S280 | Crushed chest | 8 | 5 | 62.5 |  |  |  |  |
| S281 | Traumatic amputation of part of thorax | 17 | 16 | 94.11765 |  |  |  |  |
| S29 | Other and unspecified injuries of thorax | 87 | 82 | 94.25287 |  |  |  |  |
| S290 | Injury of muscle and tendon at thorax level | 34 | 34 | 100 |  |  |  |  |
| S297 | Multiple injuries of thorax | 4 | 3 | 75 |  |  |  |  |
| S298 | Other specified injuries of thorax | 135 | 132 | 97.77778 |  |  |  |  |
| S299 | Unspecified injury of thorax | 469 | 448 | 95.52239 |  |  |  |  |
| S30 | Superficial injury of abdomen, lower back and pelvis | 2246 | 2226 | 99.10953 |  |  |  |  |
| S300 | Contusion of lower back and pelvis | 33383 | 33382 | 99.997 |  |  |  |  |
| S301 | Contusion of abdominal wall | 16968 | 16954 | 99.91749 |  |  |  |  |
| S302 | Contusion of external genital organs | 4939 | 4939 | 100 |  |  |  |  |
| S307 | Multiple superficial injuries of abdomen, lower back and pelvis | 97 | 97 | 100 |  |  |  |  |
| S308 | Other superficial injuries of abdomen, lower back and pelvis | 3997 | 3996 | 99.97498 |  |  |  |  |
| S309 | Superficial injury of abdomen, lower back and pelvis, part unspecified | 939 | 938 | 99.8935 |  |  |  |  |
| S31 | Open wound of abdomen, lower back and pelvis | 2246 | 2196 | 97.77382 |  |  |  |  |
| S32 | Fracture of lumbar spine and pelvis | 1702 | 1700 | 99.88249 |  |  |  |  |
| S320 | Fracture of lumbar vertebra | 28966 | 28963 | 99.98964 |  |  |  |  |
| S321 | Fracture of sacrum | 1321 | 1320 | 99.9243 |  |  |  |  |
| S322 | Fracture of coccyx | 2984 | 2984 | 100 |  |  |  |  |
| S323 | Fracture of ilium | 511 | 511 | 100 |  |  |  |  |
| S324 | Fracture of acetabulum | 1081 | 1081 | 100 |  |  |  |  |
| S325 | Fracture of pubis | 2027 | 2024 | 99.852 |  |  |  |  |
| S327 | Multiple fractures of lumbar spine and pelvis | 699 | 696 | 99.57082 |  |  |  |  |
| S328 | Fracture of other and unspecified parts of lumbar spine and pelvis | 5940 | 5847 | 98.43434 |  |  |  |  |
| S33 | Dislocation, sprain and strain of joints and ligaments of lumbar spine and pelvis | 338 | 334 | 98.81657 |  |  |  |  |
| S330 | Traumatic rupture of lumbar intervertebral disc | 143 | 143 | 100 |  |  |  |  |
| S331 | Dislocation of lumbar vertebra | 64 | 64 | 100 |  |  |  |  |
| S332 | Dislocation of sacroiliac and sacrococcygeal joint | 11 | 11 | 100 |  |  |  |  |
| S333 | Dislocation of other and unspecified parts of lumbar spine and pelvis | 65 | 65 | 100 |  |  |  |  |
| S334 | Traumatic rupture of symphysis pubis | 12 | 11 | 91.66667 |  |  |  |  |
| S335 | Sprain and strain of lumbar spine | 103421 | 103420 | 99.99903 |  |  |  |  |
| S336 | Sprain and strain of sacroiliac joint | 150 | 150 | 100 |  |  |  |  |
| S337 | Sprain and strain of other and unspecified parts of lumbar spine and pelvis | 6815 | 6815 | 100 |  |  |  |  |
| S34 | Injury of nerves and lumbar spinal cord at abdomen, lower back and pelvis level | 46876 | 46871 | 99.98933 |  |  |  |  |
| S340 | Concussion and oedema of lumbar spinal cord | 10 | 10 | 100 |  |  |  |  |
| S341 | Other injury of lumbar spinal cord | 123 | 123 | 100 |  |  |  |  |
| S342 | Injury of nerve root of lumbar and sacral spine | 12 | 12 | 100 |  |  |  |  |
| S343 | Injury of cauda equina | 4 | 4 | 100 |  |  |  |  |
| S344 | Injury of lumbosacral plexus | 1 | 1 | 100 |  |  |  |  |
| S345 | Injury of lumbar, sacral and pelvic sympathetic nerves | 7 | 7 | 100 |  |  |  |  |
| S346 | Injury of peripheral nerve(s) of abdomen, lower back and pelvis | 2 | 2 | 100 |  |  |  |  |
| S348 | Injury of other and unspecified nerves at abdomen, lower back and pelvis level | 6 | 6 | 100 |  |  |  |  |
| S35 | Injury of blood vessels at abdomen, lower back and pelvis level | 64570 | 64567 | 99.99535 |  |  |  |  |
| S350 | Injury of abdominal aorta | 17 | 16 | 94.11765 |  |  |  |  |
| S351 | Injury of inferior vena cava | 15 | 14 | 93.33333 |  |  |  |  |
| S352 | Injury of coeliac or mesenteric artery | 110 | 106 | 96.36364 |  |  |  |  |
| S353 | Injury of portal or splenic vein | 26 | 25 | 96.15385 |  |  |  |  |
| S354 | Injury of renal blood vessels | 30 | 29 | 96.66667 |  |  |  |  |
| S355 | Injury of iliac blood vessels | 25 | 24 | 96 |  |  |  |  |
| S357 | Injury of multiple blood vessels at abdomen, lower back and pelvis level | 6 | 4 | 66.66667 |  |  |  |  |
| S358 | Injury of other blood vessels at abdomen, lower back and pelvis level | 76 | 74 | 97.36842 |  |  |  |  |
| S359 | Injury of unspecified blood vessel at abdomen, lower back and pelvis level | 107 | 106 | 99.06542 |  |  |  |  |
| S36 | Injury of intra-abdominal organs | 37232 | 37232 | 100 |  |  |  |  |
| S360 | Injury of spleen | 814 | 809 | 99.38575 |  |  |  |  |
| S361 | Injury of liver or gallbladder | 1095 | 1083 | 98.90411 |  |  |  |  |
| S362 | Injury of pancreas | 133 | 133 | 100 |  |  |  |  |
| S363 | Injury of stomach | 21 | 21 | 100 |  |  |  |  |
| S364 | Injury of small intestine | 212 | 211 | 99.5283 |  |  |  |  |
| S365 | Injury of colon | 79 | 79 | 100 |  |  |  |  |
| S366 | Injury of rectum | 100 | 100 | 100 |  |  |  |  |
| S367 | Injury of multiple intra-abdominal organs | 53 | 48 | 90.56604 |  |  |  |  |
| S368 | Injury of other intra-abdominal organs | 827 | 796 | 96.25151 |  |  |  |  |
| S369 | Injury of unspecified intra-abdominal organ | 261 | 250 | 95.78544 |  |  |  |  |
| S37 | Injury of urinary and pelvic organs | 92 | 92 | 100 |  |  |  |  |
| S370 | Injury of kidney | 662 | 660 | 99.69789 |  |  |  |  |
| S371 | Injury of ureter | 52 | 52 | 100 |  |  |  |  |
| S372 | Injury of bladder | 136 | 135 | 99.26471 |  |  |  |  |
| S373 | Injury of urethra | 1390 | 1390 | 100 |  |  |  |  |
| S374 | Injury of ovary | 4 | 4 | 100 |  |  |  |  |
| S375 | Injury of fallopian tube | 1 | 1 | 100 |  |  |  |  |
| S376 | Injury of uterus | 11 | 11 | 100 |  |  |  |  |
| S377 | Injury of multiple pelvic organs | 11 | 11 | 100 |  |  |  |  |
| S378 | Injury of other pelvic organs | 103 | 103 | 100 |  |  |  |  |
| S379 | Injury of unspecified pelvic organ | 46 | 45 | 97.82609 |  |  |  |  |
| S38 | Crushing injury and traumatic amputation of part of abdomen, lower back and pelvis | 145 | 144 | 99.31034 |  |  |  |  |
| S380 | Crushing injury of external genital organs | 52 | 52 | 100 |  |  |  |  |
| S381 | Crushing injury of other and unspecified parts of abdomen, lower back and pelvis | 8 | 8 | 100 |  |  |  |  |
| S382 | Traumatic amputation of external genital organs | 16 | 16 | 100 |  |  |  |  |
| S383 | Traumatic amputation of other and unspecified parts of abdomen, lower back and pelvis | 1 | 1 | 100 |  |  |  |  |
| S39 | Other and unspecified injuries of abdomen, lower back and pelvis | 939 | 939 | 100 |  |  |  |  |
| S390 | Injury of muscle and tendon of abdomen, lower back and pelvis | 75 | 75 | 100 |  |  |  |  |
| S396 | Injury of intra-abdominal organs(s) with pelvic organ(s) | 13 | 11 | 84.61538 |  |  |  |  |
| S397 | Other multiple injuries of abdomen, lower back and pelvis | 12 | 10 | 83.33333 |  |  |  |  |
| S398 | Other specified injuries of abdomen, lower back and pelvis | 161 | 161 | 100 |  |  |  |  |
| S399 | Unspecified injury of abdomen, lower back and pelvis | 2555 | 2552 | 99.88258 |  |  |  |  |
| S40 | Superficial injury of shoulder and upper arm | 570 | 570 | 100 |  |  |  |  |
| S400 | Contusion of shoulder and upper arm | 36272 | 36272 | 100 |  |  |  |  |
| S407 | Multiple superficial injuries of shoulder and upper arm | 61 | 60 | 98.36066 |  |  |  |  |
| S408 | Other superficial injuries of shoulder and upper arm | 2011 | 2011 | 100 |  |  |  |  |
| S409 | Superficial injury of shoulder and upper arm, unspecified | 596 | 596 | 100 |  |  |  |  |
| S41 | Open wound of shoulder and upper arm | 56 | 56 | 100 |  |  |  |  |
| S410 | Open wound of shoulder | 1027 | 1027 | 100 |  |  |  |  |
| S411 | Open wound of upper arm | 4087 | 4086 | 99.97553 |  |  |  |  |
| S417 | Multiple open wounds of shoulder and upper arm | 267 | 267 | 100 |  |  |  |  |
| S418 | Open wound of other and unspecified parts of shoulder girdle | 112 | 112 | 100 |  |  |  |  |
| S42 | Fracture of shoulder and upper arm | 67342 | 67342 | 100 |  |  |  |  |
| S420 | Fracture of clavicle | 25154 | 25154 | 100 |  |  |  |  |
| S421 | Fracture of scapula | 1756 | 1756 | 100 |  |  |  |  |
| S422 | Fracture of upper end of humerus | 11436 | 11436 | 100 |  |  |  |  |
| S423 | Fracture of shaft of humerus | 10729 | 10726 | 99.97204 |  |  |  |  |
| S424 | Fracture of lower end of humerus | 14877 | 14877 | 100 |  |  |  |  |
| S427 | Multiple fractures of clavicle, scapula and humerus | 18 | 18 | 100 |  |  |  |  |
| S428 | Fracture of other parts of shoulder and upper arm | 182 | 182 | 100 |  |  |  |  |
| S429 | Fracture of shoulder girdle, part unspecified | 306 | 306 | 100 |  |  |  |  |
| S43 | Dislocation, sprain and strain of joints and ligaments of shoulder girdle | 54493 | 54492 | 99.99816 |  |  |  |  |
| S430 | Dislocation of shoulder joint | 19680 | 19680 | 100 |  |  |  |  |
| S431 | Anterior dislocation of humerus | 956 | 956 | 100 |  |  |  |  |
| S432 | Dislocation of sternoclavicular joint | 35 | 35 | 100 |  |  |  |  |
| S433 | Dislocation of other and unspecified parts of shoulder girdle | 1521 | 1521 | 100 |  |  |  |  |
| S434 | Sprain and strain of shoulder joint | 10724 | 10724 | 100 |  |  |  |  |
| S435 | Sprain and strain of acromioclavicular joint | 904 | 904 | 100 |  |  |  |  |
| S436 | Sprain and strain of sternoclavicular joint | 42 | 42 | 100 |  |  |  |  |
| S437 | Sprain and strain of other and unspecified | 3365 | 3365 | 100 |  |  |  |  |
| S439 | parts of shoulder girdle | 0 | 0 | 0 |  |  |  |  |
| S44 | Injury of nerves at shoulder and upper arm level | 146054 | 146053 | 99.99932 |  |  |  |  |
| S440 | Injury of ulnar nerve at upper arm level | 3 | 3 | 100 |  |  |  |  |
| S441 | Injury of median nerve at upper arm level | 2 | 2 | 100 |  |  |  |  |
| S442 | Injury of radial nerve at upper arm level | 6 | 6 | 100 |  |  |  |  |
| S443 | Injury of axillary nerve | 13 | 13 | 100 |  |  |  |  |
| S444 | Injury of musculocutaneous nerve | 3 | 3 | 100 |  |  |  |  |
| S445 | Injury of cutaneous sensory nerve at shoulder and upper arm level | 2 | 2 | 100 |  |  |  |  |
| S447 | Injury of multiple nerves at shoulder and upper arm level | 2 | 2 | 100 |  |  |  |  |
| S448 | Injury of other nerves at shoulder and upper arm level | 6 | 6 | 100 |  |  |  |  |
| S449 | Injury of unspecified nerve at shoulder and upper arm level | 12 | 12 | 100 |  |  |  |  |
| S45 | Injury of blood vessels at shoulder and upper arm level | 10525 | 10525 | 100 |  |  |  |  |
| S450 | Injury of axillary artery | 8 | 8 | 100 |  |  |  |  |
| S451 | Injury of brachial artery | 31 | 31 | 100 |  |  |  |  |
| S452 | Injury of axillary or brachial vein | 9 | 9 | 100 |  |  |  |  |
| S453 | Injury of superficial vein at shoulder and upper arm level | 40 | 40 | 100 |  |  |  |  |
| S457 | Injury of multiple blood vessels at shoulder and upper arm level | 2 | 2 | 100 |  |  |  |  |
| S458 | Injury of other blood vessels at shoulder and upper arm level | 58 | 58 | 100 |  |  |  |  |
| S459 | Injury of unspecified blood vessel at shoulder and upper arm level | 45 | 44 | 97.77778 |  |  |  |  |
| S46 | Injury of muscle and tendon at shoulder and upper arm level | 689 | 689 | 100 |  |  |  |  |
| S460 | Injury of muscle(s) and tendon(s) of the rotator cuff of shoulder | 598 | 598 | 100 |  |  |  |  |
| S461 | Injury of muscle and tendon of long head of biceps | 92 | 92 | 100 |  |  |  |  |
| S462 | Injury of muscle and tendon of other parts of biceps | 108 | 108 | 100 |  |  |  |  |
| S463 | Injury of muscle and tendon of triceps | 49 | 49 | 100 |  |  |  |  |
| S467 | Injury of multiple muscles and tendons at shoulder and upper arm level | 92 | 92 | 100 |  |  |  |  |
| S468 | Injury of other muscles and tendon at shoulder and upper arm level | 227 | 227 | 100 |  |  |  |  |
| S469 | Injury of unspecified muscle and tendon at shoulder and upper arm level | 216 | 216 | 100 |  |  |  |  |
| S47 | Crushing injury of shoulder and upper arm | 101 | 101 | 100 |  |  |  |  |
| S48 | Traumatic amputation of shoulder and upper arm | 778 | 778 | 100 |  |  |  |  |
| S480 | Traumatic amputation at shoulder joint | 94 | 94 | 100 |  |  |  |  |
| S481 | Traumatic amputation at level between shoulder and elbow | 12 | 12 | 100 |  |  |  |  |
| S489 | Traumatic amputation of shoulder and upper arm, level unspecified | 25 | 25 | 100 |  |  |  |  |
| S49 | Other and unspecified injuries of shoulder and upper arm | 1467 | 1467 | 100 |  |  |  |  |
| S497 | Multiple injuries of shoulder and upper arm | 17 | 17 | 100 |  |  |  |  |
| S498 | Other specified injuries of shoulder and upper arm | 121 | 121 | 100 |  |  |  |  |
| S499 | Unspecified injury of shoulder and upper arm | 1393 | 1393 | 100 |  |  |  |  |
| S50 | Superficial injury of forearm | 221 | 221 | 100 |  |  |  |  |
| S500 | Contusion of elbow | 35034 | 35034 | 100 |  |  |  |  |
| S501 | Contusion of other and unspecified parts of forearm | 14179 | 14179 | 100 |  |  |  |  |
| S507 | Multiple superficial injuries of forearm | 383 | 383 | 100 |  |  |  |  |
| S508 | Other superficial injuries of forearm | 5320 | 5320 | 100 |  |  |  |  |
| S509 | Superficial injury of forearm, unspecified | 2235 | 2235 | 100 |  |  |  |  |
| S51 | Open wound of forearm | 222 | 221 | 99.54955 |  |  |  |  |
| S510 | Open wound of elbow | 8677 | 8677 | 100 |  |  |  |  |
| S517 | Multiple open wounds of forearm | 2084 | 2084 | 100 |  |  |  |  |
| S518 | Open wound of other parts of forearm | 9992 | 9992 | 100 |  |  |  |  |
| S519 | Open wound of forearm, part unspecified | 19034 | 19034 | 100 |  |  |  |  |
| S52 | Fracture of forearm | 581725 | 581724 | 99.99983 |  |  |  |  |
| S520 | Fracture of upper end of ulna | 8555 | 8554 | 99.98831 |  |  |  |  |
| S521 | Fracture of upper end of radius | 6479 | 6479 | 100 |  |  |  |  |
| S522 | Fracture of shaft of ulna | 1904 | 1904 | 100 |  |  |  |  |
| S523 | Fracture of shaft of radius | 3509 | 3509 | 100 |  |  |  |  |
| S524 | Fracture of shafts of both ulna and radius | 2936 | 2936 | 100 |  |  |  |  |
| S525 | Fracture of lower end of radius | 49221 | 49221 | 100 |  |  |  |  |
| S526 | Fracture of lower end of both ulna and radius | 9680 | 9680 | 100 |  |  |  |  |
| S527 | Multiple fracture of forearm | 470 | 469 | 99.78723 |  |  |  |  |
| S528 | Fracture of other parts of forearm | 3148 | 3148 | 100 |  |  |  |  |
| S529 | Fracture of forearm, part unspecified | 2224 | 2224 | 100 |  |  |  |  |
| S53 | Dislocation, sprain and strain of joints and ligaments of elbow | 140230 | 140225 | 99.99643 |  |  |  |  |
| S530 | Dislocation of radial head | 42458 | 42458 | 100 |  |  |  |  |
| S531 | Dislocation of elbow, unspecified | 38799 | 38799 | 100 |  |  |  |  |
| S532 | Traumatic rupture of radial collateral ligament | 20 | 20 | 100 |  |  |  |  |
| S533 | Traumatic rupture of ulnar collateral ligament | 34 | 34 | 100 |  |  |  |  |
| S534 | Sprain and strain of elbow | 10397 | 10397 | 100 |  |  |  |  |
| S54 | Injury of nerves at forearm level | 64582 | 64582 | 100 |  |  |  |  |
| S540 | Injury of ulnar nerve at forearm level | 31 | 31 | 100 |  |  |  |  |
| S541 | Injury of median nerve at forearm level | 19 | 19 | 100 |  |  |  |  |
| S542 | Injury of radial nerve at forearm level | 66 | 66 | 100 |  |  |  |  |
| S543 | Injury of cutaneous sensory nerve at forearm level | 5 | 5 | 100 |  |  |  |  |
| S547 | Injury of multiple nerves at forearm level | 0 | 0 | 0 |  |  |  |  |
| S548 | Injury of other nerves at forearm level | 7 | 7 | 100 |  |  |  |  |
| S549 | Injury of unspecified nerve at forearm level | 6 | 6 | 100 |  |  |  |  |
| S55 | Injury of blood vessels at forearm level | 44621 | 44621 | 100 |  |  |  |  |
| S550 | Injury of ulnar artery at forearm level | 14 | 14 | 100 |  |  |  |  |
| S551 | Injury of radial artery at forearm level | 49 | 49 | 100 |  |  |  |  |
| S552 | Injury of vein at forearm level | 68 | 68 | 100 |  |  |  |  |
| S557 | Injury of multiple blood vessels at forearm level | 5 | 5 | 100 |  |  |  |  |
| S558 | Injury of other blood vessels at forearm level | 348 | 348 | 100 |  |  |  |  |
| S559 | Injury of unspecified blood vessels at forearm level | 199 | 199 | 100 |  |  |  |  |
| S56 | Injury of muscle and tendon at forearm level | 3588 | 3588 | 100 |  |  |  |  |
| S560 | Injury of flexor muscle and tendon of thumb at forearm level | 101 | 101 | 100 |  |  |  |  |
| S561 | Injury of flexor muscle and tendon of other finger(s) at forearm level | 384 | 384 | 100 |  |  |  |  |
| S562 | Injury of other flexor muscle and tendon at forearm level | 275 | 275 | 100 |  |  |  |  |
| S563 | Injury of extensor or abductor muscles and tendons of thumb at forearm level | 119 | 119 | 100 |  |  |  |  |
| S564 | Injury of extensor muscle and tendon of other finger(s) at forearm level | 625 | 625 | 100 |  |  |  |  |
| S565 | Injury of other extensor muscle and tendon at forearm level | 159 | 159 | 100 |  |  |  |  |
| S567 | Injury of multiple muscles and tendons at forearm level | 145 | 145 | 100 |  |  |  |  |
| S568 | Injury of other and unspecified muscles and tendon at forearm level | 538 | 538 | 100 |  |  |  |  |
| S57 | Crushing injury of forearm | 182 | 181 | 99.45055 |  |  |  |  |
| S570 | Crushing injury of elbow | 44 | 44 | 100 |  |  |  |  |
| S578 | Crushing injury of other parts of forearm | 45 | 45 | 100 |  |  |  |  |
| S579 | Crushing injury of forearm, part unspecified | 93 | 93 | 100 |  |  |  |  |
| S58 | Traumatic amputation of forearm | 1257 | 1257 | 100 |  |  |  |  |
| S580 | Traumatic amputation at elbow level | 28 | 28 | 100 |  |  |  |  |
| S581 | Traumatic amputation at level between elbow and wrist | 11 | 11 | 100 |  |  |  |  |
| S589 | Traumatic amputation of forearm, level unspecified | 48 | 47 | 97.91667 |  |  |  |  |
| S59 | Other and unspecified injuries of forearm | 10314 | 10314 | 100 |  |  |  |  |
| S597 | Multiple injuries of forearm | 34 | 34 | 100 |  |  |  |  |
| S598 | Other specified injuries of forearm | 134 | 134 | 100 |  |  |  |  |
| S599 | Unspecified injury of forearm | 2261 | 2261 | 100 |  |  |  |  |
| S60 | Superficial injury of wrist and hand | 10021 | 10021 | 100 |  |  |  |  |
| S600 | Contusion of finger(s) without damage to nail | 75233 | 75233 | 100 |  |  |  |  |
| S601 | Contusion of finger(s) with damage to nail | 11588 | 11588 | 100 |  |  |  |  |
| S602 | Contusion of other parts of wrist and hand | 57338 | 57338 | 100 |  |  |  |  |
| S607 | Multiple superficial injuries of wrist and hand | 889 | 889 | 100 |  |  |  |  |
| S608 | Other superficial injuries of wrist and hand | 27252 | 27251 | 99.99633 |  |  |  |  |
| S609 | Superficial injury of wrist and hand, unspecified | 13114 | 13114 | 100 |  |  |  |  |
| S61 | Open wound of wrist and hand | 7913 | 7913 | 100 |  |  |  |  |
| S610 | Open wound of finger(s) without damage to nail | 352709 | 352709 | 100 |  |  |  |  |
| S611 | Open wound of finger(s) with damage to nail | 33731 | 33731 | 100 |  |  |  |  |
| S617 | Multiple open wounds of wrist and hand | 7610 | 7610 | 100 |  |  |  |  |
| S618 | Open wound of other parts of wrist and hand | 62388 | 62385 | 99.99519 |  |  |  |  |
| S619 | Open wound of wrist and hand, part unspecified | 74203 | 74201 | 99.9973 |  |  |  |  |
| S62 | Fracture at wrist and hand level | 7473 | 7473 | 100 |  |  |  |  |
| S620 | Fracture of navicular [scaphoid] bone of hand | 1011 | 1011 | 100 |  |  |  |  |
| S621 | Fracture of other carpal bone(s) | 1707 | 1707 | 100 |  |  |  |  |
| S622 | Fracture of first metacarpal bone | 921 | 921 | 100 |  |  |  |  |
| S623 | Fracture of other metacarpal bone | 12493 | 12493 | 100 |  |  |  |  |
| S624 | Multiple fracture of metacarpal bones | 557 | 557 | 100 |  |  |  |  |
| S625 | Fracture of thumb | 4238 | 4238 | 100 |  |  |  |  |
| S626 | Fracture of other finger | 39068 | 39068 | 100 |  |  |  |  |
| S627 | Multiple fracture of fingers | 1291 | 1291 | 100 |  |  |  |  |
| S628 | Fracture of other and unspecified parts of wrist and hand | 6256 | 6256 | 100 |  |  |  |  |
| S63 | Dislocation, sprain and strain of joints and ligaments at wrist and hand level | 56131 | 56126 | 99.99109 |  |  |  |  |
| S630 | Dislocation of wrist | 679 | 679 | 100 |  |  |  |  |
| S631 | Dislocation of finger | 4724 | 4724 | 100 |  |  |  |  |
| S632 | Multiple dislocations of fingers | 36 | 36 | 100 |  |  |  |  |
| S633 | Traumatic rupture of ligament of wrist and carpus | 78 | 78 | 100 |  |  |  |  |
| S634 | Traumatic rupture of ligament of finger at metacarpophalangeal and interphalangeal joint(s) | 326 | 326 | 100 |  |  |  |  |
| S635 | Sprain and strain of wrist | 20983 | 20983 | 100 |  |  |  |  |
| S636 | Sprain and strain of finger(s) | 18474 | 18474 | 100 |  |  |  |  |
| S637 | Sprain and strain of other and unspecified parts of hand | 3226 | 3226 | 100 |  |  |  |  |
| S64 | Injury of nerves at wrist and hand level | 81534 | 81516 | 99.97792 |  |  |  |  |
| S640 | Injury of ulnar nerve at wrist and hand level | 33 | 33 | 100 |  |  |  |  |
| S641 | Injury of median nerve at wrist and hand level | 17 | 17 | 100 |  |  |  |  |
| S642 | Injury of radial nerve at wrist and hand level | 24 | 24 | 100 |  |  |  |  |
| S643 | Injury of digital nerve of thumb | 49 | 49 | 100 |  |  |  |  |
| S644 | Injury of digital nerve of other finger | 212 | 212 | 100 |  |  |  |  |
| S647 | Injury of multiple nerves at wrist and hand level | 2 | 2 | 100 |  |  |  |  |
| S648 | Injury of other nerves at wrist and hand level | 25 | 25 | 100 |  |  |  |  |
| S649 | Injury of unspecified nerve at wrist and hand level | 50 | 50 | 100 |  |  |  |  |
| S65 | Injury of blood vessels at wrist and hand level | 25407 | 25387 | 99.92128 |  |  |  |  |
| S650 | Injury of ulnar artery at wrist and hand level | 69 | 69 | 100 |  |  |  |  |
| S651 | Injury of radial artery at wrist and hand level | 63 | 62 | 98.4127 |  |  |  |  |
| S652 | Injury of superficial palmar arch | 487 | 487 | 100 |  |  |  |  |
| S653 | Injury of deep palmar arch | 145 | 145 | 100 |  |  |  |  |
| S654 | Injury of blood vessel(s) of thumb | 625 | 625 | 100 |  |  |  |  |
| S655 | Injury of blood vessel(s) of other finger | 8094 | 8094 | 100 |  |  |  |  |
| S657 | Injury of multiple blood vessels at wrist and hand level | 6 | 6 | 100 |  |  |  |  |
| S658 | Injury of other blood vessels at wrist and hand level | 1414 | 1414 | 100 |  |  |  |  |
| S659 | Injury of unspecified blood vessel at wrist and hand level | 800 | 800 | 100 |  |  |  |  |
| S66 | Injury of muscle and tendon at wrist and hand level | 1007 | 1004 | 99.70209 |  |  |  |  |
| S660 | Injury of long flexor muscle and tendon of thumb at wrist and hand level | 219 | 219 | 100 |  |  |  |  |
| S661 | Injury of flexor muscle and tendon of other finger at wrist and hand level | 1921 | 1921 | 100 |  |  |  |  |
| S662 | Injury of extensor muscle and tendon of thumb at wrist and hand level | 1188 | 1188 | 100 |  |  |  |  |
| S663 | Injury of extensor muscle and tendon of other finger at wrist and hand level | 5687 | 5687 | 100 |  |  |  |  |
| S664 | Injury of intrinsic muscle and tendon of thumb at wrist and hand level | 140 | 140 | 100 |  |  |  |  |
| S665 | Injury of intrinsic muscle and tendon of other finger at wrist and hand level | 866 | 866 | 100 |  |  |  |  |
| S666 | Injury of multiple flexor muscles and tendons at wrist and hand level | 244 | 244 | 100 |  |  |  |  |
| S667 | Injury of multiple extensor muscles and tendons at wrist and hand level | 250 | 250 | 100 |  |  |  |  |
| S668 | Injury of other muscles and tendons at wrist and hand level | 1038 | 1038 | 100 |  |  |  |  |
| S669 | Injury of unspecified muscle and tendon at wrist and hand level | 1743 | 1743 | 100 |  |  |  |  |
| S67 | Crushing injury of wrist and hand | 336 | 336 | 100 |  |  |  |  |
| S670 | Crushing injury of thumb and other finger(s) | 4638 | 4638 | 100 |  |  |  |  |
| S678 | Crushing injury of other and unspecified parts of wrist and hand | 754 | 754 | 100 |  |  |  |  |
| S68 | Traumatic amputation of wrist and hand | 757 | 757 | 100 |  |  |  |  |
| S680 | Traumatic amputation of thumb (complete) (partial) | 714 | 714 | 100 |  |  |  |  |
| S681 | Traumatic amputation of other single finger (complete)(partial) | 4769 | 4769 | 100 |  |  |  |  |
| S682 | Traumatic amputation of two or more fingers alone (complete)(partial) | 519 | 519 | 100 |  |  |  |  |
| S683 | Combined traumatic amputation of (part of) finger(s) with other parts of wrist and hand | 365 | 365 | 100 |  |  |  |  |
| S684 | Traumatic amputation of hand at wrist level | 48 | 48 | 100 |  |  |  |  |
| S688 | Traumatic amputation of other parts of wrist and hand | 139 | 139 | 100 |  |  |  |  |
| S689 | Traumatic amputation of wrist and hand, level unspecified | 161 | 161 | 100 |  |  |  |  |
| S69 | Other and unspecified injuries of wrist and hand | 88 | 86 | 97.72727 |  |  |  |  |
| S697 | Multiple injuries of wrist and hand | 102 | 102 | 100 |  |  |  |  |
| S698 | Other specified injuries of wrist and hand | 596 | 596 | 100 |  |  |  |  |
| S699 | Unspecified injury of wrist and hand | 15794 | 15794 | 100 |  |  |  |  |
| S70 | Superficial injury of hip and thigh | 116724 | 116717 | 99.994 |  |  |  |  |
| S700 | Contusion of hip | 26294 | 26290 | 99.98479 |  |  |  |  |
| S701 | Contusion of thigh | 9200 | 9200 | 100 |  |  |  |  |
| S707 | Multiple superficial injuries of hip and thigh | 257 | 257 | 100 |  |  |  |  |
| S708 | Other superficial injuries of hip and thigh | 2743 | 2742 | 99.96354 |  |  |  |  |
| S709 | Superficial injury of hip and thigh, unspecified | 1145 | 1145 | 100 |  |  |  |  |
| S71 | Open wound of hip and thigh | 53819 | 53814 | 99.99071 |  |  |  |  |
| S710 | Open wound of hip | 1270 | 1269 | 99.92126 |  |  |  |  |
| S711 | Open wound of thigh | 9834 | 9834 | 100 |  |  |  |  |
| S717 | Multiple open wounds of hip and thigh | 203 | 203 | 100 |  |  |  |  |
| S718 | Open wound of other and unspecified parts of pelvic girdle | 96 | 96 | 100 |  |  |  |  |
| S72 | Fracture of femur | 87372 | 87351 | 99.97596 |  |  |  |  |
| S720 | Fracture of neck of femur | 30373 | 30371 | 99.99342 |  |  |  |  |
| S721 | Pertrochanteric fracture | 31513 | 31506 | 99.97779 |  |  |  |  |
| S722 | Subtrochanteric fracture | 1599 | 1598 | 99.93746 |  |  |  |  |
| S723 | Fracture of shaft of femur | 6646 | 6639 | 99.89467 |  |  |  |  |
| S724 | Fracture of lower end of femur | 3436 | 3436 | 100 |  |  |  |  |
| S727 | Multiple fractures of femur | 62 | 61 | 98.3871 |  |  |  |  |
| S728 | Fractures of other parts of femur | 1926 | 1923 | 99.84424 |  |  |  |  |
| S729 | Fracture of femur, part unspecified | 10870 | 10856 | 99.87121 |  |  |  |  |
| S73 | Dislocation, sprain and strain of joint and ligaments of hip | 12627 | 12622 | 99.9604 |  |  |  |  |
| S730 | Dislocation of hip | 4058 | 4056 | 99.95071 |  |  |  |  |
| S731 | Sprain and strain of hip | 5030 | 5030 | 100 |  |  |  |  |
| S74 | Injury of nerves at hip and thigh level | 3427 | 3425 | 99.94164 |  |  |  |  |
| S740 | Injury of sciatic nerve at hip and thigh level | 21 | 21 | 100 |  |  |  |  |
| S741 | Injury of femoral nerve at hip and thigh level | 4 | 4 | 100 |  |  |  |  |
| S742 | Injury of cutaneous sensory nerve at hip and thigh level | 0 | 0 | 0 |  |  |  |  |
| S747 | Injury of multiple nerves at hip and thigh level | 0 | 0 | 0 |  |  |  |  |
| S748 | Injury of other nerves at hip and thigh level | 5 | 5 | 100 |  |  |  |  |
| S749 | Injury of unspecified nerve at hip and thigh level | 2 | 2 | 100 |  |  |  |  |
| S75 | Injury of blood vessels at hip and thigh level | 27822 | 27822 | 100 |  |  |  |  |
| S750 | Injury of femoral artery | 46 | 43 | 93.47826 |  |  |  |  |
| S751 | Injury of femoral vein at hip and thigh level | 21 | 21 | 100 |  |  |  |  |
| S752 | Injury of greater saphenous vein at hip and thigh level | 2 | 2 | 100 |  |  |  |  |
| S757 | Injury of multiple blood vessels at hip and thigh level | 2 | 2 | 100 |  |  |  |  |
| S758 | Injury of other blood vessels at hip and thigh level | 117 | 117 | 100 |  |  |  |  |
| S759 | Injury of unspecified blood vessel at hip and thigh level | 125 | 125 | 100 |  |  |  |  |
| S76 | Injury of muscle and tendon at hip and thigh level | 307 | 307 | 100 |  |  |  |  |
| S760 | Injury of muscle and tendon of hip | 101 | 101 | 100 |  |  |  |  |
| S761 | Injury of quadriceps muscle and tendon | 586 | 586 | 100 |  |  |  |  |
| S762 | Injury of adductor muscle and tendon of thigh | 93 | 93 | 100 |  |  |  |  |
| S763 | Injury of muscle and tendon of the posterior muscle group at thigh level | 243 | 243 | 100 |  |  |  |  |
| S764 | Injury of other and unspecified muscles and tendons at thigh level | 571 | 571 | 100 |  |  |  |  |
| S767 | Injury of multiple muscles and tendons at hip and thigh level | 121 | 121 | 100 |  |  |  |  |
| S77 | Crushing injury of hip and thigh | 15 | 15 | 100 |  |  |  |  |
| S770 | Crushing injury of hip | 7 | 7 | 100 |  |  |  |  |
| S771 | Crushing injury of thigh | 41 | 41 | 100 |  |  |  |  |
| S772 | Crushing injury of hip with thigh | 7 | 7 | 100 |  |  |  |  |
| S78 | Traumatic amputation of hip and thigh | 986 | 986 | 100 |  |  |  |  |
| S780 | Traumatic amputation at hip joint | 1 | 1 | 100 |  |  |  |  |
| S781 | Traumatic amputation at level between hip and knee | 9 | 9 | 100 |  |  |  |  |
| S789 | Traumatic amputation of hip and thigh, level unspecified | 5 | 3 | 60 |  |  |  |  |
| S79 | Other and unspecified injuries of hip and thigh | 7904 | 7904 | 100 |  |  |  |  |
| S797 | Multiple injuries of hip and thigh | 7 | 7 | 100 |  |  |  |  |
| S798 | Other specified injuries of hip and thigh | 76 | 76 | 100 |  |  |  |  |
| S799 | Unspecified injury of hip and thigh | 849 | 848 | 99.88221 |  |  |  |  |
| S80 | Superficial injury of lower leg | 3728 | 3726 | 99.94635 |  |  |  |  |
| S800 | Contusion of knee | 69607 | 69606 | 99.99856 |  |  |  |  |
| S801 | Contusion of other and unspecified parts of lower leg | 33327 | 33325 | 99.994 |  |  |  |  |
| S807 | Multiple superficial injuries of lower leg | 739 | 737 | 99.72936 |  |  |  |  |
| S808 | Other superficial injuries of lower leg | 12202 | 12201 | 99.9918 |  |  |  |  |
| S809 | Superficial injury of lower leg, unspecified | 3577 | 3577 | 100 |  |  |  |  |
| S81 | Open wound of lower leg | 229 | 228 | 99.56332 |  |  |  |  |
| S810 | Open wound of knee | 31715 | 31713 | 99.99369 |  |  |  |  |
| S817 | Multiple open wounds of lower leg | 1664 | 1664 | 100 |  |  |  |  |
| S818 | Open wound of other parts of lower leg | 16863 | 16860 | 99.98221 |  |  |  |  |
| S819 | Open wound of lower leg, part unspecified | 27074 | 27073 | 99.99631 |  |  |  |  |
| S82 | Fracture of lower leg, including ankle | 142329 | 142329 | 100 |  |  |  |  |
| S820 | Fracture of patella | 10075 | 10075 | 100 |  |  |  |  |
| S821 | Fracture of upper end of tibia | 5805 | 5804 | 99.98277 |  |  |  |  |
| S822 | Fracture of shaft of tibia | 10950 | 10941 | 99.91781 |  |  |  |  |
| S823 | Fracture of lower end of tibia | 9194 | 9189 | 99.94562 |  |  |  |  |
| S824 | Fracture of fibula alone | 5955 | 5950 | 99.91604 |  |  |  |  |
| S825 | Fracture of medial malleolus | 3739 | 3739 | 100 |  |  |  |  |
| S826 | Fracture of lateral malleolus | 14580 | 14580 | 100 |  |  |  |  |
| S827 | Multiple fractures of lower leg | 162 | 160 | 98.76543 |  |  |  |  |
| S828 | Fractures of other parts of lower leg | 12465 | 12462 | 99.97593 |  |  |  |  |
| S829 | Fracture of lower leg, part unspecified | 1142 | 1140 | 99.82487 |  |  |  |  |
| S83 | Dislocation, sprain and strain of joints and ligaments of knee | 100512 | 100510 | 99.99801 |  |  |  |  |
| S830 | Dislocation of patella | 910 | 910 | 100 |  |  |  |  |
| S831 | Dislocation of knee | 673 | 673 | 100 |  |  |  |  |
| S832 | Tear of meniscus, current | 671 | 671 | 100 |  |  |  |  |
| S833 | Tear of articular cartilage of knee, current | 31 | 31 | 100 |  |  |  |  |
| S834 | Sprain and strain involving (fibular)(tibial) collateral ligament of knee | 1386 | 1386 | 100 |  |  |  |  |
| S835 | Sprain and strain involving (anterior) (posterior) cruciate ligament of knee | 1307 | 1307 | 100 |  |  |  |  |
| S836 | Sprain and strain of other and unspecified parts of knee | 24537 | 24537 | 100 |  |  |  |  |
| S837 | Injury to multiple structures of knee | 553 | 553 | 100 |  |  |  |  |
| S84 | Injury of nerves at lower leg level | 41166 | 41165 | 99.99757 |  |  |  |  |
| S840 | Injury of tibial nerve at lower leg level | 9 | 9 | 100 |  |  |  |  |
| S841 | Injury of peroneal nerve at lower leg level | 30 | 30 | 100 |  |  |  |  |
| S842 | Injury of cutaneous sensory nerve at lower leg level | 3 | 3 | 100 |  |  |  |  |
| S847 | Injury of multiple nerves at lower leg level | 0 | 0 | 0 |  |  |  |  |
| S848 | Injury of other nerves at lower leg level | 3 | 3 | 100 |  |  |  |  |
| S849 | Injury of unspecified nerve at lower leg level | 2 | 2 | 100 |  |  |  |  |
| S85 | Injury of blood vessels at lower leg level | 169913 | 169913 | 100 |  |  |  |  |
| S850 | Injury of popliteal artery | 261 | 261 | 100 |  |  |  |  |
| S851 | Injury of (anterior)(posterior) tibial artery | 36 | 36 | 100 |  |  |  |  |
| S852 | Injury of peroneal artery | 2 | 2 | 100 |  |  |  |  |
| S853 | Injury of greater saphenous vein at lower leg level | 7 | 7 | 100 |  |  |  |  |
| S854 | Injury of lesser saphenous vein at lower leg level | 2 | 2 | 100 |  |  |  |  |
| S855 | Injury of popliteal vein | 6 | 6 | 100 |  |  |  |  |
| S857 | Injury of multiple blood vessels at lower leg level | 5 | 5 | 100 |  |  |  |  |
| S858 | Injury of other blood vessels at lower leg level | 562 | 562 | 100 |  |  |  |  |
| S859 | Injury of unspecified blood vessel at lower leg level | 419 | 419 | 100 |  |  |  |  |
| S86 | Injury of muscle and tendon at lower leg level | 138 | 138 | 100 |  |  |  |  |
| S860 | Injury of Achilles tendon | 6562 | 6562 | 100 |  |  |  |  |
| S861 | Injury of other muscle(s) and tendon(s) of posterior muscle group at lower leg level | 921 | 921 | 100 |  |  |  |  |
| S862 | Injury of muscle(s) and tendon(s) of anterior muscle group at lower leg level | 228 | 228 | 100 |  |  |  |  |
| S863 | Injury of muscle(s) and tendon(s) of peroneal muscle group at lower leg level | 193 | 193 | 100 |  |  |  |  |
| S867 | Injury of multiple muscles and tendons at lower leg level | 401 | 400 | 99.75062 |  |  |  |  |
| S868 | Injury of other muscles and tendons at lower leg level | 1254 | 1254 | 100 |  |  |  |  |
| S869 | Injury of unspecified muscles and tendons at lower leg level | 1776 | 1776 | 100 |  |  |  |  |
| S87 | Crushing injury of lower leg | 77 | 77 | 100 |  |  |  |  |
| S870 | Crushing injury of knee | 137 | 137 | 100 |  |  |  |  |
| S878 | Crushing injury of other and unspecified parts of lower leg | 160 | 159 | 99.375 |  |  |  |  |
| S88 | Traumatic amputation of lower leg | 1229 | 1229 | 100 |  |  |  |  |
| S880 | Traumatic amputation at knee level | 14 | 14 | 100 |  |  |  |  |
| S881 | Traumatic amputation at level between knee and ankle | 32 | 32 | 100 |  |  |  |  |
| S889 | Traumatic amputation of lower leg, level unspecified | 182 | 181 | 99.45055 |  |  |  |  |
| S89 | Other and unspecified injuries of lower leg | 1305 | 1305 | 100 |  |  |  |  |
| S897 | Multiple injuries of lower leg | 31 | 31 | 100 |  |  |  |  |
| S898 | Other specified injuries of lower leg | 278 | 278 | 100 |  |  |  |  |
| S899 | Unspecified injury of lower leg | 3701 | 3701 | 100 |  |  |  |  |
| S90 | Superficial injury of ankle and foot | 1545 | 1545 | 100 |  |  |  |  |
| S900 | Contusion of ankle | 25221 | 25221 | 100 |  |  |  |  |
| S901 | Contusion of toe(s) without damage to nail | 28083 | 28083 | 100 |  |  |  |  |
| S902 | Contusion of toe(s) with damage to nail | 12113 | 12113 | 100 |  |  |  |  |
| S903 | Contusion of other and unspecified parts of foot | 53788 | 53788 | 100 |  |  |  |  |
| S907 | Multiple superficial injuries of ankle and foot | 418 | 418 | 100 |  |  |  |  |
| S908 | Other superficial injuries of ankle and foot | 13323 | 13323 | 100 |  |  |  |  |
| S909 | Superficial injury of ankle and foot, unspecified | 5373 | 5373 | 100 |  |  |  |  |
| S91 | Open wound of ankle and foot | 437 | 437 | 100 |  |  |  |  |
| S910 | Open wound of ankle | 9342 | 9341 | 99.9893 |  |  |  |  |
| S911 | Open wound of toe(s) without damage to nail | 21209 | 21209 | 100 |  |  |  |  |
| S912 | Open wound of toe(s) with damage to nail | 6461 | 6461 | 100 |  |  |  |  |
| S913 | Open wound of other parts of foot | 50342 | 50341 | 99.99801 |  |  |  |  |
| S917 | Multiple open wounds of ankle and foot | 1060 | 1060 | 100 |  |  |  |  |
| S92 | Fracture of foot, except ankle | 114124 | 113874 | 99.78094 |  |  |  |  |
| S920 | Fracture of calcaneus | 8756 | 8756 | 100 |  |  |  |  |
| S921 | Fracture of talus | 902 | 902 | 100 |  |  |  |  |
| S922 | Fracture of other tarsal bone(s) | 2440 | 2440 | 100 |  |  |  |  |
| S923 | Fracture of metatarsal bone | 17925 | 17925 | 100 |  |  |  |  |
| S924 | Fracture of great toe | 6427 | 6427 | 100 |  |  |  |  |
| S925 | Fracture of other toe | 13711 | 13711 | 100 |  |  |  |  |
| S927 | Multiple fractures of foot | 357 | 357 | 100 |  |  |  |  |
| S929 | Fracture of foot, unspecified | 2746 | 2745 | 99.96358 |  |  |  |  |
| S93 | Dislocation, sprain and strain of joints and ligaments at ankle and foot level | 4761 | 4754 | 99.85297 |  |  |  |  |
| S930 | Dislocation of ankle joint | 310 | 310 | 100 |  |  |  |  |
| S931 | Dislocation of toe(s) | 1070 | 1070 | 100 |  |  |  |  |
| S932 | Rupture of ligaments at ankle and foot level | 369 | 369 | 100 |  |  |  |  |
| S933 | Dislocation of other and unspecified parts of foot | 171 | 171 | 100 |  |  |  |  |
| S934 | Sprain and strain of ankle | 145789 | 145789 | 100 |  |  |  |  |
| S935 | Sprain and strain of toe(s) | 4737 | 4737 | 100 |  |  |  |  |
| S936 | Sprain and strain of other and unspecified parts of foot | 17466 | 17466 | 100 |  |  |  |  |
| S94 | Injury of nerves at ankle and foot level | 68 | 67 | 98.52941 |  |  |  |  |
| S940 | Injury of lateral plantar nerve | 0 | 0 | 0 |  |  |  |  |
| S941 | Injury of medial plantar nerve | 1 | 1 | 100 |  |  |  |  |
| S942 | Injury of deep peroneal nerve at ankle and foot level | 3 | 3 | 100 |  |  |  |  |
| S943 | Injury of cutaneous sensory nerve at ankle and foot level | 6 | 6 | 100 |  |  |  |  |
| S947 | Injury of multiple nerves at ankle and foot level | 2 | 2 | 100 |  |  |  |  |
| S948 | Injury of other nerves at ankle and foot level | 8 | 8 | 100 |  |  |  |  |
| S949 | Injury of unspecified nerve at ankle and foot level | 6 | 6 | 100 |  |  |  |  |
| S95 | Injury of blood vessels at ankle and foot level | 1612 | 1584 | 98.26303 |  |  |  |  |
| S950 | Injury of dorsal artery of foot | 43 | 43 | 100 |  |  |  |  |
| S951 | Injury of plantar artery of foot | 70 | 70 | 100 |  |  |  |  |
| S952 | Injury of dorsal vein of foot | 74 | 74 | 100 |  |  |  |  |
| S957 | Injury of multiple blood vessels at ankle and foot level | 3 | 3 | 100 |  |  |  |  |
| S958 | Injury of other blood vessels at ankle and foot level | 490 | 490 | 100 |  |  |  |  |
| S959 | Injury of unspecified blood vessel at ankle and foot level | 459 | 459 | 100 |  |  |  |  |
| S96 | Injury of muscle and tendon at ankle and foot level | 408 | 407 | 99.7549 |  |  |  |  |
| S960 | Injury of muscle and tendon of long flexor muscle of toe at ankle and foot level | 280 | 280 | 100 |  |  |  |  |
| S961 | Injury of muscle and tendon of long extensor muscle of toe at ankle and foot level | 600 | 600 | 100 |  |  |  |  |
| S962 | Injury of intrinsic muscle and tendon at ankle and foot level | 162 | 162 | 100 |  |  |  |  |
| S967 | Injury of multiple muscles and tendons at ankle and foot level | 84 | 84 | 100 |  |  |  |  |
| S968 | Injury of other muscles and tendons at ankle and foot level | 459 | 459 | 100 |  |  |  |  |
| S969 | Injury of unspecified muscle tendon at ankle and foot level | 627 | 627 | 100 |  |  |  |  |
| S97 | Crushing injury of ankle and foot | 60 | 59 | 98.33333 |  |  |  |  |
| S970 | Crushing injury of ankle | 63 | 63 | 100 |  |  |  |  |
| S971 | Crushing injury of toe(s) | 401 | 401 | 100 |  |  |  |  |
| S978 | Crushing injury of other parts of ankle and foot | 305 | 305 | 100 |  |  |  |  |
| S98 | Traumatic amputation of ankle and foot | 29 | 28 | 96.55172 |  |  |  |  |
| S980 | Traumatic amputation of foot at ankle level | 19 | 19 | 100 |  |  |  |  |
| S981 | Traumatic amputation of one toe | 108 | 108 | 100 |  |  |  |  |
| S982 | Traumatic amputation of two or more toes | 22 | 22 | 100 |  |  |  |  |
| S983 | Traumatic amputation of other parts of foot | 22 | 22 | 100 |  |  |  |  |
| S984 | Traumatic amputation of foot, level unspecified | 23 | 23 | 100 |  |  |  |  |
| S99 | Other and unspecified injuries of ankle and foot | 2 | 2 | 100 |  |  |  |  |
| S997 | Multiple injuries of ankle and foot | 37 | 37 | 100 |  |  |  |  |
| S998 | Other specified injuries of ankle and foot | 350 | 350 | 100 |  |  |  |  |
| S999 | Unspecified injury of ankle and foot | 7035 | 7035 | 100 |  |  |  |  |
| T00 | Superficial injuries involving multiple body regions | 63 | 62 | 98.4127 |  |  |  |  |
| T000 | Superficial injuries involving head with neck | 3912 | 3912 | 100 |  |  |  |  |
| T001 | Superficial injuries involving thorax with abdomen, lower back and pelvis | 37 | 37 | 100 |  |  |  |  |
| T002 | Superficial injuries involving multiple regions of upper limb(s) | 24 | 24 | 100 |  |  |  |  |
| T003 | Superficial injuries involving multiple regions of lower limb(s) | 48 | 48 | 100 |  |  |  |  |
| T006 | Superficial injuries involving multiple regions of upper limb(s) with lower limb(s) | 22 | 22 | 100 |  |  |  |  |
| T008 | Superficial injuries involving other combinations of body regions | 1108 | 1107 | 99.90975 |  |  |  |  |
| T009 | Multiple superficial injuries, unspecified | 101938 | 101689 | 99.75573 |  |  |  |  |
| T01 | Open wounds involving multiple body regions | 6961 | 6700 | 96.25054 |  |  |  |  |
| T010 | Open wounds involving head with neck | 122 | 122 | 100 |  |  |  |  |
| T011 | Open wounds involving thorax with abdomen, lower back and pelvis | 20 | 20 | 100 |  |  |  |  |
| T012 | Open wounds involving multiple regions of upper limb(s) | 549 | 549 | 100 |  |  |  |  |
| T013 | Open wounds involving multiple regions of lower limb(s) | 156 | 156 | 100 |  |  |  |  |
| T016 | Open wounds involving multiple regions of upper limb(s) with lower limb(s) | 19 | 19 | 100 |  |  |  |  |
| T018 | Open wounds involving other combinations of body regions | 14 | 14 | 100 |  |  |  |  |
| T019 | Multiple open wounds, unspecified | 3881 | 3874 | 99.81963 |  |  |  |  |
| T02 | Fractures involving multiple body regions | 8665 | 8660 | 99.9423 |  |  |  |  |
| T020 | Fractures involving head with neck | 2 | 2 | 100 |  |  |  |  |
| T021 | Fractures involving thorax with lower back and pelvis | 42 | 42 | 100 |  |  |  |  |
| T022 | Fractures involving multiple regions of one upper limb | 4 | 4 | 100 |  |  |  |  |
| T023 | Fractures involving multiple regions of one lower limb | 10 | 9 | 90 |  |  |  |  |
| T024 | Fractures involving multiple regions of both upper limbs | 3 | 3 | 100 |  |  |  |  |
| T025 | Fractures involving multiple regions of both lower limbs | 6 | 6 | 100 |  |  |  |  |
| T026 | Fractures involving multiple regions of upper limb(s) with lower limb(s) | 1 | 1 | 100 |  |  |  |  |
| T027 | Fractures involving thorax with lower back and pelvis with limb(s) | 5 | 5 | 100 |  |  |  |  |
| T028 | Fractures involving other combinations of body regions | 150 | 149 | 99.33333 |  |  |  |  |
| T029 | Multiple fractures, unspecified | 95 | 68 | 71.57895 |  |  |  |  |
| T03 | Dislocations, sprains and strains involving multiple body regions | 787 | 786 | 99.87294 |  |  |  |  |
| T030 | Dislocations, sprains and strains involving head with neck | 272 | 272 | 100 |  |  |  |  |
| T031 | Dislocations, sprains and strains involving thorax with lower back and pelvis | 1036 | 1036 | 100 |  |  |  |  |
| T032 | Dislocations, sprains and strains involving multiple regions of upper limb(s) | 54 | 54 | 100 |  |  |  |  |
| T033 | Dislocations, sprains and strains involving multiple regions of lower limb(s) | 88 | 88 | 100 |  |  |  |  |
| T034 | Dislocations, sprains and strains involving multiple regions of upper limb(s) with lower limb(s) | 7 | 7 | 100 |  |  |  |  |
| T038 | Dislocations, sprains and strains involving other combinations of body regions | 36 | 36 | 100 |  |  |  |  |
| T039 | Multiple dislocations, sprains and strains, unspecified | 272 | 272 | 100 |  |  |  |  |
| T04 | Crushing injuries involving multiple body regions | 5215 | 5214 | 99.98082 |  |  |  |  |
| T040 | Crushing injuries involving head with neck | 0 | 0 | 0 |  |  |  |  |
| T041 | Crushing injuries involving thorax with abdomen, lower back and pelvis | 5 | 4 | 80 |  |  |  |  |
| T042 | Crushing injuries involving multiple regions of upper limb(s) | 25 | 24 | 96 |  |  |  |  |
| T043 | Crushing injuries involving multiple regions of lower limb(s) | 32 | 32 | 100 |  |  |  |  |
| T044 | Crushing injuries involving multiple regions of upper limb(s) with lower limb(s) | 3 | 3 | 100 |  |  |  |  |
| T047 | Crushing injuries of thorax with abdomen, lower back and pelvis with limb(s) | 0 | 0 | 0 |  |  |  |  |
| T048 | Crushing injuries involving other combinations of body regions | 4 | 4 | 100 |  |  |  |  |
| T049 | Multiple crushing injuries, unspecified | 8 | 7 | 87.5 |  |  |  |  |
| T05 | Traumatic amputations involving multiple body regions | 248537 | 248491 | 99.98149 |  |  |  |  |
| T050 | Traumatic amputation of both hands | 5 | 5 | 100 |  |  |  |  |
| T051 | Traumatic amputation of one hand and other arm [any level, except hand] | 12 | 12 | 100 |  |  |  |  |
| T052 | Traumatic amputation of both arm [any level] | 0 | 0 | 0 |  |  |  |  |
| T053 | Traumatic amputation of both feet | 0 | 0 | 0 |  |  |  |  |
| T054 | Traumatic amputation of one foot and other leg [any level, except foot] | 1 | 1 | 100 |  |  |  |  |
| T055 | Traumatic amputation of both legs [any level] | 1 | 1 | 100 |  |  |  |  |
| T056 | Traumatic amputation of upper and lower limbs, any combination [any level] | 0 | 0 | 0 |  |  |  |  |
| T058 | Traumatic amputation involving other combinations of body regions | 1 | 1 | 100 |  |  |  |  |
| T059 | Multiple traumatic amputations, unspecified | 13 | 13 | 100 |  |  |  |  |
| T06 | Other injuries involving multiple body regions, NEC | 27259 | 27240 | 99.9303 |  |  |  |  |
| T060 | Injuries of brain and cranial nerves with injuries of nerves and spinal cord at neck level | 7 | 6 | 85.71429 |  |  |  |  |
| T061 | Injuries of nerves and spinal cord involving other multiple body regions | 29 | 29 | 100 |  |  |  |  |
| T062 | Injuries of nerves involving multiple body regions | 0 | 0 | 0 |  |  |  |  |
| T063 | Injuries of blood vessels involving multiple body regions | 1 | 1 | 100 |  |  |  |  |
| T064 | Injuries of muscles and tendons involving multiple body regions | 12 | 12 | 100 |  |  |  |  |
| T065 | Injuries of intrathoracic organs with intra-abdominal and pelvic organs | 3 | 1 | 33.33333 |  |  |  |  |
| T068 | Other specified injuries involving multiple body regions | 2211 | 2129 | 96.29127 |  |  |  |  |
| T07 | Unspecified multiple injuries | 944 | 944 | 100 |  |  |  |  |
| T08 | Fracture of spine, level unspecified | 38282 | 38282 | 100 |  |  |  |  |
| T080 | Closed | 2029 | 2028 | 99.95071 |  |  |  |  |
| T081 | Open | 5 | 5 | 100 |  |  |  |  |
| T09 | Other injuries of spine and trunk, level unspecified | 101795 | 101758 | 99.96365 |  |  |  |  |
| T090 | Superficial injury of trunk, level unspecified | 543 | 543 | 100 |  |  |  |  |
| T091 | Open wound of trunk, level unspecified | 387 | 385 | 99.4832 |  |  |  |  |
| T092 | Dislocation, sprain and strain of unspecified joint and ligament of trunk | 5599 | 5599 | 100 |  |  |  |  |
| T093 | Injury of spinal cord, level unspecified | 882 | 881 | 99.88662 |  |  |  |  |
| T094 | Injury of unspecified nerve, spinal nerve root and plexus of trunk | 24 | 24 | 100 |  |  |  |  |
| T095 | Injury of unspecified muscle and tendon of trunk | 17 | 17 | 100 |  |  |  |  |
| T096 | Traumatic amputation of trunk, level unspecified | 0 | 0 | 0 |  |  |  |  |
| T098 | Other specified injuries of trunk, level unspecified | 41 | 41 | 100 |  |  |  |  |
| T099 | Unspecified injury of trunk, level unspecified | 253 | 249 | 98.41897 |  |  |  |  |
| T10 | Fracture of upper limb, level unspecified | 17498 | 17498 | 100 |  |  |  |  |
| T100 | Closed | 264 | 264 | 100 |  |  |  |  |
| T101 | Open | 11 | 11 | 100 |  |  |  |  |
| T11 | Other injuries of upper limb, level unspecified | 32866 | 32863 | 99.99087 |  |  |  |  |
| T110 | Superficial injury of upper limb, level unspecified | 748 | 748 | 100 |  |  |  |  |
| T111 | Open wound of upper limb, level unspecified | 826 | 826 | 100 |  |  |  |  |
| T112 | Dislocation, sprain and strain of unspecified joint and ligament of trunk joint and ligament of upper limb, level unspecified | 273 | 273 | 100 |  |  |  |  |
| T113 | Injury of unspecified nerve of upper limb, level unspecified | 5 | 5 | 100 |  |  |  |  |
| T114 | Injury of unspecified blood vessel of upper limb, level unspecified | 3 | 3 | 100 |  |  |  |  |
| T115 | Injury of unspecified muscle and tendon of upper limb, level unspecified | 33 | 33 | 100 |  |  |  |  |
| T116 | Traumatic amputation of upper limb, level unspecified | 27 | 26 | 96.2963 |  |  |  |  |
| T118 | Other specified injuries of upper limb, level unspecified | 23 | 23 | 100 |  |  |  |  |
| T119 | Unspecified injury of upper limb, level unspecified | 423 | 423 | 100 |  |  |  |  |
| T12 | Fracture of lower limb, level unspecified | 100 | 100 | 100 |  |  |  |  |
| T120 | Closed | 199 | 199 | 100 |  |  |  |  |
| T121 | Open | 38 | 38 | 100 |  |  |  |  |
| T13 | Other injuries of lower limb, level unspecified | 13863 | 13860 | 99.97836 |  |  |  |  |
| T130 | Superficial injury of lower limb, level unspecified | 1401 | 1401 | 100 |  |  |  |  |
| T131 | Open wound of lower limb, level unspecified | 2433 | 2432 | 99.9589 |  |  |  |  |
| T132 | Dislocation, sprain and strain of unspecified joint and ligament of lower limb, level unspecified | 556 | 556 | 100 |  |  |  |  |
| T133 | Injury of unspecified nerve of lower limb, level unspecified | 2 | 2 | 100 |  |  |  |  |
| T134 | Injury of unspecified blood vessel of lower limb, level unspecified | 5 | 5 | 100 |  |  |  |  |
| T135 | Injury of unspecified muscle and tendon of lower limb, level unspecified | 581 | 581 | 100 |  |  |  |  |
| T136 | Traumatic amputation of lower limb, level unspecified | 25 | 24 | 96 |  |  |  |  |
| T138 | Other specified injuries of lower limb, level unspecified | 18 | 18 | 100 |  |  |  |  |
| T139 | Unspecified injury of lower limb, level unspecified | 1053 | 1052 | 99.90503 |  |  |  |  |
| T14 | Injury of unspecified body region | 21253 | 21251 | 99.99059 |  |  |  |  |
| T140 | Superficial injury of unspecified body region | 128168 | 128139 | 99.97737 |  |  |  |  |
| T141 | Open wound of unspecified body region | 116129 | 116117 | 99.98967 |  |  |  |  |
| T142 | Fracture of unspecified body region | 771 | 768 | 99.61089 |  |  |  |  |
| T143 | Dislocation, sprain and strain of unspecified body region | 2354 | 2354 | 100 |  |  |  |  |
| T144 | Injury of nerve(s) of unspecified body region | 19 | 19 | 100 |  |  |  |  |
| T145 | Injury of blood vessel(s) of unspecified body region | 166 | 165 | 99.39759 |  |  |  |  |
| T146 | Injury of muscles and tendons of unspecified body region | 4764 | 4764 | 100 |  |  |  |  |
| T147 | Crushing injury and traumatic amputation of unspecified body region | 453 | 452 | 99.77925 |  |  |  |  |
| T148 | Other injuries of unspecified body region | 3642 | 3637 | 99.86271 |  |  |  |  |
| T149 | Injury, unspecified | 782 | 770 | 98.46547 |  |  |  |  |
| T15 | Foreign body on external eye | 168 | 167 | 99.40476 |  |  |  |  |
| T150 | Foreign body in cornea | 12883 | 12883 | 100 |  |  |  |  |
| T151 | Foreign body in conjunctival sac | 4569 | 4569 | 100 |  |  |  |  |
| T158 | Foreign body in other and multiple parts of external eye | 944 | 944 | 100 |  |  |  |  |
| T159 | Foreign body on external eye, part unspecified | 18739 | 18739 | 100 |  |  |  |  |
| T16 | Foreign body in ear | 7 | 7 | 100 |  |  |  |  |
| T17 | Foreign body in respiratory tract | 58925 | 58925 | 100 |  |  |  |  |
| T170 | Foreign body in nasal sinus | 2384 | 2384 | 100 |  |  |  |  |
| T171 | Foreign body in nostril | 23695 | 23695 | 100 |  |  |  |  |
| T172 | Foreign body in pharynx | 68620 | 68617 | 99.99563 |  |  |  |  |
| T173 | Foreign body in larynx | 5318 | 5318 | 100 |  |  |  |  |
| T174 | Foreign body in trachea | 284 | 282 | 99.29577 |  |  |  |  |
| T175 | Foreign body in bronchus | 361 | 361 | 100 |  |  |  |  |
| T178 | Foreign body in other and multiple parts of respiratory tract | 272 | 267 | 98.16176 |  |  |  |  |
| T179 | Foreign body in respiratory tract, part unspecified | 861 | 834 | 96.86411 |  |  |  |  |
| T18 | Foreign body in alimentary tract | 25363 | 25363 | 100 |  |  |  |  |
| T180 | Foreign body in mouth | 4194 | 4194 | 100 |  |  |  |  |
| T181 | Foreign body in esophagus | 13304 | 13304 | 100 |  |  |  |  |
| T182 | Foreign body in stomach | 5682 | 5682 | 100 |  |  |  |  |
| T183 | Foreign body in small intestine | 515 | 515 | 100 |  |  |  |  |
| T184 | Foreign body in colon | 308 | 308 | 100 |  |  |  |  |
| T185 | Foreign body in anus and rectum | 503 | 503 | 100 |  |  |  |  |
| T188 | Foreign body in other and multiple parts of alimentary tract | 432 | 432 | 100 |  |  |  |  |
| T189 | Foreign body in alimentary tract, part unspecified | 23042 | 23039 | 99.98698 |  |  |  |  |
| T19 | Foreign body in genitourinary tract | 16794 | 16794 | 100 |  |  |  |  |
| T190 | Foreign body in urethra | 58 | 58 | 100 |  |  |  |  |
| T191 | Foreign body in bladder | 41 | 41 | 100 |  |  |  |  |
| T192 | Foreign body in vulva and vagina | 2285 | 2285 | 100 |  |  |  |  |
| T193 | Foreign body in uterus[any part] | 30 | 30 | 100 |  |  |  |  |
| T198 | Foreign body in other and multiple parts of genitourinary tract | 70 | 70 | 100 |  |  |  |  |
| T199 | Foreign body in genitourinary tract, part unspecified | 113 | 113 | 100 |  |  |  |  |
| T20 | Burn and corrosion of head and neck | 2545 | 2545 | 100 |  |  |  |  |
| T200 | Burn of unspecified degree of head and neck | 2229 | 2228 | 99.95514 |  |  |  |  |
| T201 | Burn of first degree of head and neck | 3120 | 3120 | 100 |  |  |  |  |
| T202 | Burn of second degree of head and neck | 8212 | 8211 | 99.98782 |  |  |  |  |
| T203 | Burn of third degree of head and neck | 67 | 66 | 98.50746 |  |  |  |  |
| T204 | Corrosion of unspecified degree of head and neck | 34 | 34 | 100 |  |  |  |  |
| T205 | Corrosion of first degree of head and neck | 56 | 56 | 100 |  |  |  |  |
| T206 | Corrosion of second degree of head and neck | 29 | 29 | 100 |  |  |  |  |
| T207 | Corrosion of third degree of head and neck | 3 | 3 | 100 |  |  |  |  |
| T21 | Burn and corrosion of trunk | 2759 | 2755 | 99.85502 |  |  |  |  |
| T210 | Burn of unspecified degree of trunk | 1110 | 1110 | 100 |  |  |  |  |
| T211 | Burn of first degree of trunk | 1327 | 1327 | 100 |  |  |  |  |
| T212 | Burn of second degree of trunk | 6503 | 6502 | 99.98462 |  |  |  |  |
| T213 | Burn of third degree of trunk | 70 | 69 | 98.57143 |  |  |  |  |
| T214 | Corrosion of unspecified degree of trunk | 12 | 12 | 100 |  |  |  |  |
| T215 | Corrosion of first degree of trunk | 7 | 7 | 100 |  |  |  |  |
| T216 | Corrosion of second degree of trunk | 9 | 9 | 100 |  |  |  |  |
| T217 | Corrosion of third degree of trunk | 0 | 0 | 0 |  |  |  |  |
| T22 | Burn and corrosion of shoulder and upper limb, except wrist and hand | 780 | 776 | 99.48718 |  |  |  |  |
| T220 | Burn of unspecified degree of shoulder and upper limb, except wrist and hand | 1760 | 1760 | 100 |  |  |  |  |
| T221 | Burn of first degree of shoulder and upper limb, except wrist and hand | 1271 | 1271 | 100 |  |  |  |  |
| T222 | Burn of second degree of shoulder and upper limb, except wrist and hand | 9184 | 9184 | 100 |  |  |  |  |
| T223 | Burn of third degree of shoulder and upper limb, except wrist and hand | 78 | 77 | 98.71795 |  |  |  |  |
| T224 | Corrosion of unspecified degree of shoulder and upper limb, except wrist and hand | 31 | 31 | 100 |  |  |  |  |
| T225 | Corrosion of first degree of shoulder and upper limb, except wrist and hand | 19 | 19 | 100 |  |  |  |  |
| T226 | Corrosion of second degree of shoulder and upper limb, except wrist and hand | 40 | 40 | 100 |  |  |  |  |
| T227 | Corrosion of third degree of shoulder and upper limb, except wrist and hand | 7 | 7 | 100 |  |  |  |  |
| T23 | Burn and corrosion of wrist and hand | 1576 | 1574 | 99.8731 |  |  |  |  |
| T230 | Burn of unspecified degree of wrist and hand | 9335 | 9335 | 100 |  |  |  |  |
| T231 | Burn of first degree of wrist and hand | 8410 | 8410 | 100 |  |  |  |  |
| T232 | Burn of second degree of wrist and hand | 40447 | 40447 | 100 |  |  |  |  |
| T233 | Burn of third degree of wrist and hand | 424 | 424 | 100 |  |  |  |  |
| T234 | Corrosion of unspecified degree of wrist and hand | 71 | 71 | 100 |  |  |  |  |
| T235 | Corrosion of first degree of wrist and hand | 73 | 73 | 100 |  |  |  |  |
| T236 | Corrosion of second degree of wrist and hand | 150 | 150 | 100 |  |  |  |  |
| T237 | Corrosion of third degree of wrist and hand | 15 | 15 | 100 |  |  |  |  |
| T24 | Burn and corrosion of hip and lower limb, except ankle and foot | 56258 | 56247 | 99.98045 |  |  |  |  |
| T240 | Burn of unspecified degree of hip and lower limb, except ankle and foot | 2780 | 2780 | 100 |  |  |  |  |
| T241 | Burn of first degree of hip and lower limb, except ankle and foot | 2712 | 2712 | 100 |  |  |  |  |
| T242 | Burn of second degree of hip and lower limb, except ankle and foot | 19507 | 19507 | 100 |  |  |  |  |
| T243 | Burn of third degree of hip and lower limb, except ankle and foot | 241 | 241 | 100 |  |  |  |  |
| T244 | Corrosion of unspecified degree of hip and lower limb, except ankle and foot | 19 | 19 | 100 |  |  |  |  |
| T245 | Corrosion of first degree of hip and lower limb, except ankle and foot | 16 | 16 | 100 |  |  |  |  |
| T246 | Corrosion of second degree of hip and lower limb, except ankle and foot | 74 | 74 | 100 |  |  |  |  |
| T247 | Corrosion of third degree of hip and lower limb, except ankle and foot | 14 | 14 | 100 |  |  |  |  |
| T25 | Burn and corrosion of ankle and foot | 1419 | 1419 | 100 |  |  |  |  |
| T250 | Burn of unspecified degree of ankle and foot | 2193 | 2193 | 100 |  |  |  |  |
| T251 | Burn of first degree of ankle and foot | 1384 | 1384 | 100 |  |  |  |  |
| T252 | Burn of second degree of ankle and foot | 12990 | 12990 | 100 |  |  |  |  |
| T253 | Burn of third degree of ankle and foot | 190 | 190 | 100 |  |  |  |  |
| T254 | Corrosion unspecified degree of ankle and foot | 28 | 28 | 100 |  |  |  |  |
| T255 | Corrosion of first degree of ankle and foot | 9 | 9 | 100 |  |  |  |  |
| T256 | Corrosion of second degree of ankle and foot | 21 | 21 | 100 |  |  |  |  |
| T257 | Corrosion of third degree of ankle and foot | 14 | 14 | 100 |  |  |  |  |
| T26 | Burn and corrosion confined to eye and adnexa | 97 | 93 | 95.87629 |  |  |  |  |
| T260 | Burn of eyelid and periocular area | 425 | 425 | 100 |  |  |  |  |
| T261 | Burn of cornea and conjunctival sac | 1318 | 1318 | 100 |  |  |  |  |
| T262 | Burn with resulting rupture and destruction of eyeball | 4 | 4 | 100 |  |  |  |  |
| T263 | Burn of other parts of eye and adnexa | 173 | 173 | 100 |  |  |  |  |
| T264 | Burn of eye and adnexa, part unspecified | 412 | 412 | 100 |  |  |  |  |
| T265 | Corrosion of eyelid and periocular area | 178 | 178 | 100 |  |  |  |  |
| T266 | Corrosion of cornea and conjunctival sac | 2130 | 2130 | 100 |  |  |  |  |
| T267 | Corrosion with resulting rupture and destruction of eyeball | 9 | 9 | 100 |  |  |  |  |
| T268 | Corrosion of other parts of eye and adnexa | 93 | 92 | 98.92473 |  |  |  |  |
| T269 | Corrosion of eye and adnexa, part unspecified | 306 | 306 | 100 |  |  |  |  |
| T27 | Burn and corrosion of respiratory tract | 1081 | 893 | 82.6087 |  |  |  |  |
| T270 | Burn of larynx and trachea | 62 | 62 | 100 |  |  |  |  |
| T271 | Burn involving larynx and trachea with lung | 30 | 29 | 96.66667 |  |  |  |  |
| T272 | Burn of other parts of respiratory tract | 129 | 127 | 98.44961 |  |  |  |  |
| T273 | Burn of respiratory tract, part unspecified | 523 | 519 | 99.23518 |  |  |  |  |
| T274 | Corrosion of larynx and trachea | 6 | 6 | 100 |  |  |  |  |
| T275 | Corrosion involving larynx and trachea with lung | 0 | 0 | 0 |  |  |  |  |
| T276 | Corrosion of other parts of respiratory tract | 0 | 0 | 0 |  |  |  |  |
| T277 | Corrosion of respiratory tract, part unspecified | 3 | 3 | 100 |  |  |  |  |
| T28 | Burn and corrosion of other internal organs | 158 | 157 | 99.36709 |  |  |  |  |
| T280 | Burn of mouth and pharynx | 184 | 184 | 100 |  |  |  |  |
| T281 | Burn of esophagus | 22 | 22 | 100 |  |  |  |  |
| T282 | Burn of other parts of alimentary tract | 7 | 7 | 100 |  |  |  |  |
| T283 | Burn of internal genitourinary organs | 22 | 22 | 100 |  |  |  |  |
| T284 | Burn of other and unspecified internal organs | 13 | 13 | 100 |  |  |  |  |
| T285 | Corrosion of mouth and pharynx | 51 | 51 | 100 |  |  |  |  |
| T286 | Corrosion of esophagus | 91 | 91 | 100 |  |  |  |  |
| T287 | Corrosion of other parts of alimentary tract | 97 | 96 | 98.96907 |  |  |  |  |
| T288 | Corrosion of internal genitourinary organs | 0 | 0 | 0 |  |  |  |  |
| T289 | Corrosion of other and unspecified internal organs | 4 | 4 | 100 |  |  |  |  |
| T29 | Burns and corrosions of multiple body regions | 167 | 167 | 100 |  |  |  |  |
| T290 | Burns of multiple regions, unspecified degree | 654 | 653 | 99.84709 |  |  |  |  |
| T291 | Burns of multiple regions, no more than first-degree burns mentioned | 137 | 137 | 100 |  |  |  |  |
| T292 | Burns of multiple regions, no more than second-degree burns mentioned | 534 | 534 | 100 |  |  |  |  |
| T293 | Burns of multiple regions, at least one burn of third degree mentioned | 8 | 8 | 100 |  |  |  |  |
| T294 | Corrosions of multiple regions, unspecified degree | 3 | 3 | 100 |  |  |  |  |
| T295 | Corrosions of multiple regions, no more than first-degree corrosions mentioned | 2 | 2 | 100 |  |  |  |  |
| T296 | Corrosions of multiple regions, no more than second-degree corrosions mentioned | 0 | 0 | 0 |  |  |  |  |
| T297 | Corrosions of multiple regions, at least one corrosion of third degree mentioned | 0 | 0 | 0 |  |  |  |  |
| T30 | Burn and corrosion, body region unspecified | 86 | 86 | 100 |  |  |  |  |
| T300 | Burn of unspecified body region, unspecified degree | 34662 | 34657 | 99.98557 |  |  |  |  |
| T301 | Burn of first degree, body region unspecified | 3483 | 3483 | 100 |  |  |  |  |
| T302 | Burn of second degree, body region unspecified | 16874 | 16872 | 99.98815 |  |  |  |  |
| T303 | Burn of third degree, body region unspecified | 128 | 124 | 96.875 |  |  |  |  |
| T304 | Corrosion of unspecified body region, unspecified degree | 1106 | 1106 | 100 |  |  |  |  |
| T305 | Corrosion of first degree, body region unspecified | 4 | 4 | 100 |  |  |  |  |
| T306 | Corrosion of second degree, body region unspecified | 4 | 4 | 100 |  |  |  |  |
| T307 | Corrosion of third degree, body region unspecified | 2 | 2 | 100 |  |  |  |  |
| T31 | Burns classified according to extent of body surface involved | 91 | 91 | 100 |  |  |  |  |
| T310 | Burns involving less than 10％ of body surface | 1140 | 1140 | 100 |  |  |  |  |
| T311 | Burns involving 10-19％ of body surface | 139 | 139 | 100 |  |  |  |  |
| T312 | Burns involving 20-29％ of body surface | 48 | 48 | 100 |  |  |  |  |
| T313 | Burns involving 30-39％ of body surface | 52 | 52 | 100 |  |  |  |  |
| T314 | Burns involving 40-49％ of body surface | 30 | 30 | 100 |  |  |  |  |
| T315 | Burns involving 50-59％ of body surface | 22 | 22 | 100 |  |  |  |  |
| T316 | Burns involving 60-69％ of body surface | 23 | 23 | 100 |  |  |  |  |
| T317 | Burns involving 70-79％ of body surface | 11 | 10 | 90.90909 |  |  |  |  |
| T318 | Burns involving 80-89％ of body surface | 18 | 17 | 94.44444 |  |  |  |  |
| T319 | Burns involving 90％ or more of body surface | 16 | 14 | 87.5 |  |  |  |  |
| T32 | Corrosions classified according to extent of body surface involved | 56 | 56 | 100 |  |  |  |  |
| T320 | Corrosions involving less than 10％ of body Corrosions involving 10-19％ of body surface | 1 | 1 | 100 |  |  |  |  |
| T321 | Corrosions involving 10-19％ of body surface | 0 | 0 | 0 |  |  |  |  |
| T322 | Corrosions involving 20-29％ of body surface | 0 | 0 | 0 |  |  |  |  |
| T323 | Corrosions involving 30-39％ of body surface | 0 | 0 | 0 |  |  |  |  |
| T324 | Corrosions involving 40-49％ of body surface | 0 | 0 | 0 |  |  |  |  |
| T325 | Corrosions involving 50-59％ of body surface | 0 | 0 | 0 |  |  |  |  |
| T326 | Corrosions involving 60-69％ of body surface | 0 | 0 | 0 |  |  |  |  |
| T327 | Corrosions involving 70-79％ of body surface | 0 | 0 | 0 |  |  |  |  |
| T328 | Corrosions involving 80-89％ of body surface | 0 | 0 | 0 |  |  |  |  |
| T329 | Corrosions involving 90％ or more of body surface | 0 | 0 | 0 |  |  |  |  |
| T33 | Superficial frostbite | 0 | 0 | 0 |  |  |  |  |
| T34 | Frostbite with tissue necrosis | 0 | 0 | 0 |  |  |  |  |
| T35 | Frostbite involving multiple body regions and unspecified frostbite | 0 | 0 | 0 |  |  |  |  |
| T36 | Poisoning by systemic antibiotics | 0 | 0 | 0 |  |  |  |  |
| T37 | Poisoning by other systemic anti-infectives and antiparasitic | 0 | 0 | 0 |  |  |  |  |
| T38 | Poisoning by hormones and their synthetic substitutes and antagonists, NEC | 0 | 0 | 0 |  |  |  |  |
| T39 | Poisoning by nonopioid analgesics, antipyretics and antirheumatics | 0 | 0 | 0 |  |  |  |  |
| T40 | Poisoning by narcotics and psychodysleptics [hallucinogens] | 0 | 0 | 0 |  |  |  |  |
| T41 | Poisoning by anesthetics and therapeutic gases | 0 | 0 | 0 |  |  |  |  |
| T42 | Poisoning by antiepileptic, sedative-hypnotic and antiparkinsonism drugs | 0 | 0 | 0 |  |  |  |  |
| T43 | Poisoning by psychotropic drugs, NEC | 0 | 0 | 0 |  |  |  |  |
| T44 | Poisoning by drugs primarily affecting the autonomic nervous system | 0 | 0 | 0 |  |  |  |  |
| T45 | Poisoning by primarily systemic and hematological agents, NEC | 0 | 0 | 0 |  |  |  |  |
| T46 | Poisoning by agents primarily affecting the cardiovascular system | 0 | 0 | 0 |  |  |  |  |
| T47 | Poisoning by agents primarily affecting the gastrointestinal system | 0 | 0 | 0 |  |  |  |  |
| T48 | Poisoning by agents primarily acting on smooth and skeletal muscles and the respiratory system | 0 | 0 | 0 |  |  |  |  |
| T49 | Poisoning by topical agents primarily affecting skin and mucous membrane and by ophthalmological, otorhinolaryngological and dental drugs | 0 | 0 | 0 |  |  |  |  |
| T50 | Poisoning by diuretics and other and unspecified drugs, medicaments and biological substances | 0 | 0 | 0 |  |  |  |  |
| T51 | Toxic effect of alcohol | 0 | 0 | 0 |  |  |  |  |
| T52 | Toxic effect of organic solvents | 0 | 0 | 0 |  |  |  |  |
| T53 | Toxic effect of halogen derivatives of aliphatic and aromatic hydrocarbons | 0 | 0 | 0 |  |  |  |  |
| T54 | Toxic effect of corrosive substances | 0 | 0 | 0 |  |  |  |  |
| T55 | Toxic effect of soaps and detergents | 0 | 0 | 0 |  |  |  |  |
| T56 | Toxic effect of metals | 0 | 0 | 0 |  |  |  |  |
| T57 | Toxic effect of other inorganic substances | 0 | 0 | 0 |  |  |  |  |
| T58 | Toxic effect of carbon monoxide | 0 | 0 | 0 |  |  |  |  |
| T59 | Toxic effect of other gases, fumes and vapours | 0 | 0 | 0 |  |  |  |  |
| T60 | Toxic effect of pesticides | 0 | 0 | 0 |  |  |  |  |
| T61 | Toxic effect of noxious substances eaten as seafood | 0 | 0 | 0 |  |  |  |  |
| T62 | Toxic effect of other noxious substances eaten as food | 0 | 0 | 0 |  |  |  |  |
| T63 | Toxic effect of contact with venomous animals | 0 | 0 | 0 |  |  |  |  |
| T64 | Toxic effect of aflatoxin and other mycotoxin food contaminants | 0 | 0 | 0 |  |  |  |  |
| T65 | Toxic effect of other and unspecified substances | 0 | 0 | 0 |  |  |  |  |
| T66 | Unspecified effects of radiation | 0 | 0 | 0 |  |  |  |  |
| T67 | Effects of heat and light | 0 | 0 | 0 |  |  |  |  |
| T68 | Hypothermia | 0 | 0 | 0 |  |  |  |  |
| T69 | Other effects of reduced temperature | 0 | 0 | 0 |  |  |  |  |
| T70 | Effects of air pressure and water pressure | 0 | 0 | 0 |  |  |  |  |
| T71 | Asphyxiation | 0 | 0 | 0 |  |  |  |  |
| T73 | Effects of other deprivation | 0 | 0 | 0 |  |  |  |  |
| T74 | Maltreatment syndromes | 0 | 0 | 0 |  |  |  |  |
| T75 | Effects of other external causes | 0 | 0 | 0 |  |  |  |  |
| T76 | Unspecified effects of external causes | 0 | 0 | 0 |  |  |  |  |
| T77 |  | 0 | 0 | 0 |  |  |  |  |
| T78 | Adverse effects, NEC | 0 | 0 | 0 |  |  |  |  |
| T79 | Certain early complications of trauma, NEC | 293 | 293 | 100 |  |  |  |  |
| T790 | Air embolism (traumatic) | 3 | 3 | 100 |  |  |  |  |
| T791 | Fat embolism (traumatic) | 3 | 3 | 100 |  |  |  |  |
| T792 | Traumatic secondary and recurrent hemorrhage | 29 | 27 | 93.10345 |  |  |  |  |
| T793 | Post-traumatic wound infection, NEC | 414 | 414 | 100 |  |  |  |  |
| T794 | Traumatic shock | 231 | 49 | 21.21212 |  |  |  |  |
| T795 | Traumatic anuria | 0 | 0 | 0 |  |  |  |  |
| T796 | Traumatic ischemia of muscle | 344 | 343 | 99.7093 |  |  |  |  |
| T797 | Traumatic subcutaneous emphysema | 63 | 60 | 95.2381 |  |  |  |  |
| T798 | Other early complications of trauma | 12 | 12 | 100 |  |  |  |  |
| T799 | Unspecified early complication of trauma | 5 | 5 | 100 |  |  |  |  |
| T80 | Complications following infusion, transfusion and therapeutic injection | 0 | 0 | 0 |  |  |  |  |
| T81 | Complications of procedures, NEC | 0 | 0 | 0 |  |  |  |  |
| T82 | Complications of cardiac and vascular prosthetic devices, implants and grafts | 0 | 0 | 0 |  |  |  |  |
| T83 | Complications of genitourinary prosthetic devices, implants and grafts | 0 | 0 | 0 |  |  |  |  |
| T84 | Complications of internal orthopedic prosthetic devices, implants and grafts | 0 | 0 | 0 |  |  |  |  |
| T85 | Complications of other internal prosthetic devices, implants and grafts | 0 | 0 | 0 |  |  |  |  |
| T86 | Failure and rejection of transplanted organs and tissues | 0 | 0 | 0 |  |  |  |  |
| T87 | Complications peculiar to reattachment and amputation | 0 | 0 | 0 |  |  |  |  |
| T88 | Other complications of surgical and medical care, NEC | 0 | 0 | 0 |  |  |  |  |
| T90 | Sequelae of injuries of head | 0 | 0 | 0 |  |  |  |  |
| T900 | Sequelae of superficial injury of head | 31 | 31 | 100 |  |  |  |  |
| T901 | Sequelae of open wound of head | 35 | 35 | 100 |  |  |  |  |
| T902 | Sequelae of fracture of skull and facial bones | 11 | 11 | 100 |  |  |  |  |
| T903 | Sequelae of injury of cranial nerves | 1 | 1 | 100 |  |  |  |  |
| T904 | Sequelae of injury of eye and orbit | 23 | 23 | 100 |  |  |  |  |
| T905 | Sequelae of intracranial injury | 39 | 38 | 97.4359 |  |  |  |  |
| T908 | Sequelae of other specified injuries of head | 1 | 1 | 100 |  |  |  |  |
| T909 | Sequelae of unspecified injury of head | 19 | 19 | 100 |  |  |  |  |
| T91 | Sequelae of injuries of neck and trunk | 0 | 0 | 0 |  |  |  |  |
| T910 | Sequelae of superficial injury and open wound of neck and trunk | 23 | 23 | 100 |  |  |  |  |
| T911 | Sequelae of fracture of spine | 72 | 72 | 100 |  |  |  |  |
| T912 | Sequelae of other fracture of thorax and pelvis | 5 | 5 | 100 |  |  |  |  |
| T913 | Sequelae of injury of spinal cord | 46 | 46 | 100 |  |  |  |  |
| T914 | Sequelae of injury of intrathoracic organs | 1 | 1 | 100 |  |  |  |  |
| T915 | Sequelae of injury of intra-abdominal and pelvic organs | 1 | 1 | 100 |  |  |  |  |
| T918 | Sequelae of other specified injuries of neck and trunk | 0 | 0 | 0 |  |  |  |  |
| T919 | Sequelae of unspecified injury of neck and trunk | 0 | 0 | 0 |  |  |  |  |
| T92 | Sequelae of injuries of upper limb | 0 | 0 | 0 |  |  |  |  |
| T920 | Sequelae of open wound of upper limb | 6 | 6 | 100 |  |  |  |  |
| T921 | Sequelae of fracture of arm | 2 | 2 | 100 |  |  |  |  |
| T922 | Sequelae of fracture at wrist and hand level | 13 | 13 | 100 |  |  |  |  |
| T923 | Sequelae of dislocation, sprain and strain of upper limb | 31 | 31 | 100 |  |  |  |  |
| T924 | Sequelae of injury of nerve of upper limb | 0 | 0 | 0 |  |  |  |  |
| T925 | Sequelae of injury of muscle and tendon of upper limb | 2 | 2 | 100 |  |  |  |  |
| T926 | Sequelae of crushing injury and traumatic amputation of upper limb | 6 | 6 | 100 |  |  |  |  |
| T928 | Sequelae of other specified injuries of upper limb | 0 | 0 | 0 |  |  |  |  |
| T929 | Sequelae of unspecified injury of upper limb | 1 | 1 | 100 |  |  |  |  |
| T93 | Sequelae of injuries of lower limb | 0 | 0 | 0 |  |  |  |  |
| T930 | Sequelae of open wound of lower limb | 25 | 25 | 100 |  |  |  |  |
| T931 | Sequelae of fracture of femur | 17 | 17 | 100 |  |  |  |  |
| T932 | Sequelae of other fractures of lower limb | 7 | 7 | 100 |  |  |  |  |
| T933 | Sequelae of dislocation, sprain and strain of lower limb | 47 | 47 | 100 |  |  |  |  |
| T934 | Sequelae of injury of nerve of lower limb | 0 | 0 | 0 |  |  |  |  |
| T935 | Sequelae of injury of muscle and tendon of lower limb | 11 | 11 | 100 |  |  |  |  |
| T936 | Sequelae of crushing injury and traumatic amputation of lower limb | 5 | 5 | 100 |  |  |  |  |
| T938 | Sequelae of other specified injuries of lower limb | 0 | 0 | 0 |  |  |  |  |
| T939 | Sequelae of unspecified injury of lower limb | 2 | 2 | 100 |  |  |  |  |
| T94 | Sequelae of injuries involving multiple and unspecified body regions | 0 | 0 | 0 |  |  |  |  |
| T940 | Sequelae of injuries involving multiple body regions | 0 | 0 | 0 |  |  |  |  |
| T941 | Sequelae of injuries, not specified by body region | 2 | 2 | 100 |  |  |  |  |
| T95 | Sequelae of burns, corrosions and frostbite | 0 | 0 | 0 |  |  |  |  |
| T950 | Sequelae of burn, corrosion and frostbite of head and neck | 25 | 25 | 100 |  |  |  |  |
| T951 | Sequelae of burn, corrosion and frostbite of trunk | 6 | 6 | 100 |  |  |  |  |
| T952 | Sequelae of burn, corrosion and frostbite of upper limb | 25 | 25 | 100 |  |  |  |  |
| T953 | Sequelae of burn, corrosion and frostbite of lower limb | 26 | 26 | 100 |  |  |  |  |
| T954 | Sequelae of burn and corrosion classifiable only according to extent of body surface involved | 2 | 2 | 100 |  |  |  |  |
| T958 | Sequelae of other specified burn, corrosion and frostbite | 8 | 8 | 100 |  |  |  |  |
| T959 | Sequelae of unspecified burn, corrosion and frostbite | 31 | 31 | 100 |  |  |  |  |
| T96 | Sequelae of poisoning by drugs, medicaments and biological substances | 0 | 0 | 0 |  |  |  |  |
| T97 | Sequelae of toxic effects of substances chiefly nonmedicinal as to source | 0 | 0 | 0 |  |  |  |  |
| T98 | Sequelae of other and unspecified effects of external causes | 0 | 0 | 0 |  |  |  |  |
| T980 | Sequelae of effects of foreign body entering through natural orifice | 45 | 45 | 100 |  |  |  |  |
| T981 | Sequelae of other and unspecified effects of external causes | 14 | 14 | 100 |  |  |  |  |
| T982 | Sequelae of certain early complications of trauma | 1 | 1 | 100 |  |  |  |  |
| T983 | Sequelae of complications of surgical and medical care, NEC | 3 | 3 | 100 |  |  |  |  |
| ICD-10, international classification of diseases 10th version; ICISS, international classification of disease-based injury severity score, SRR, survival risk ratio | | | | |  |  |  |  |


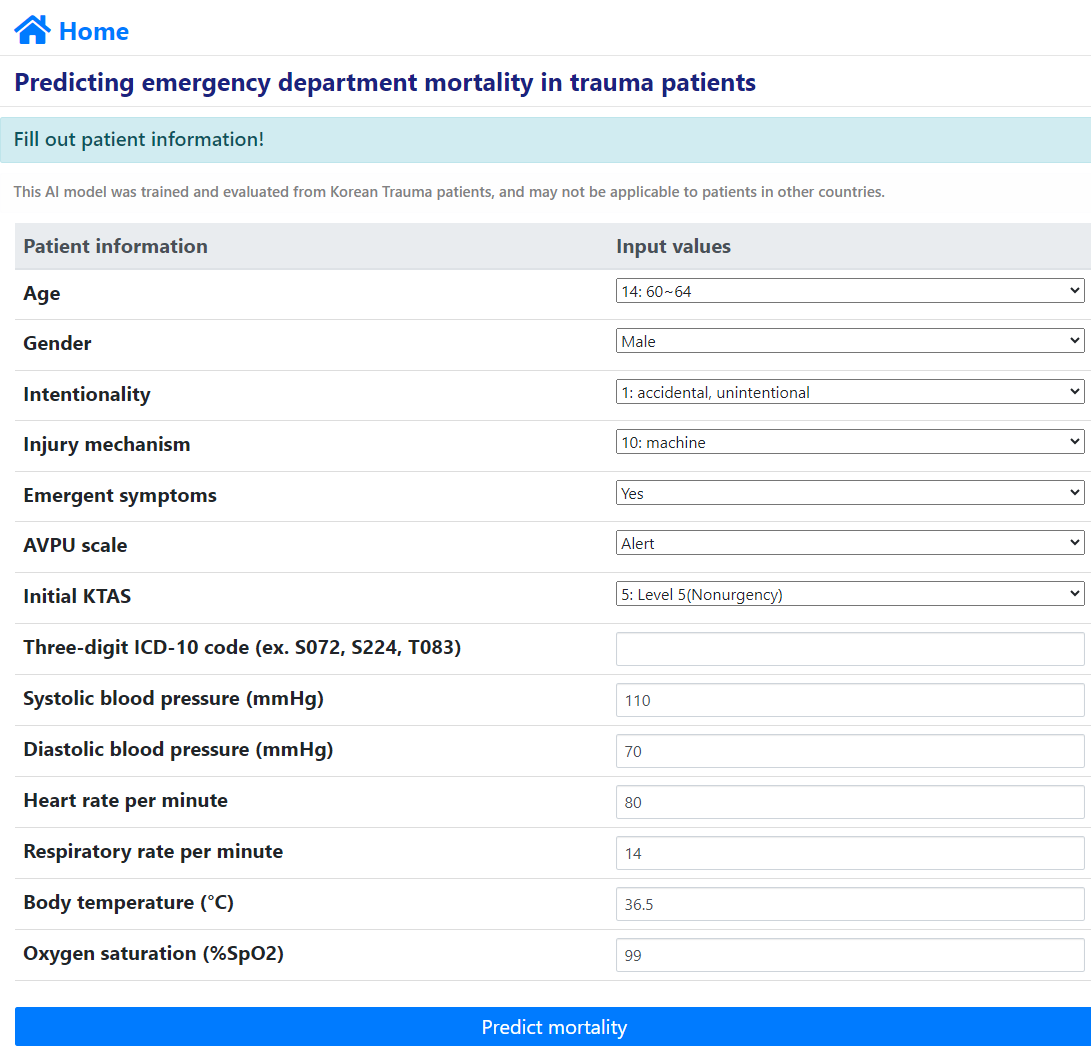


(A)


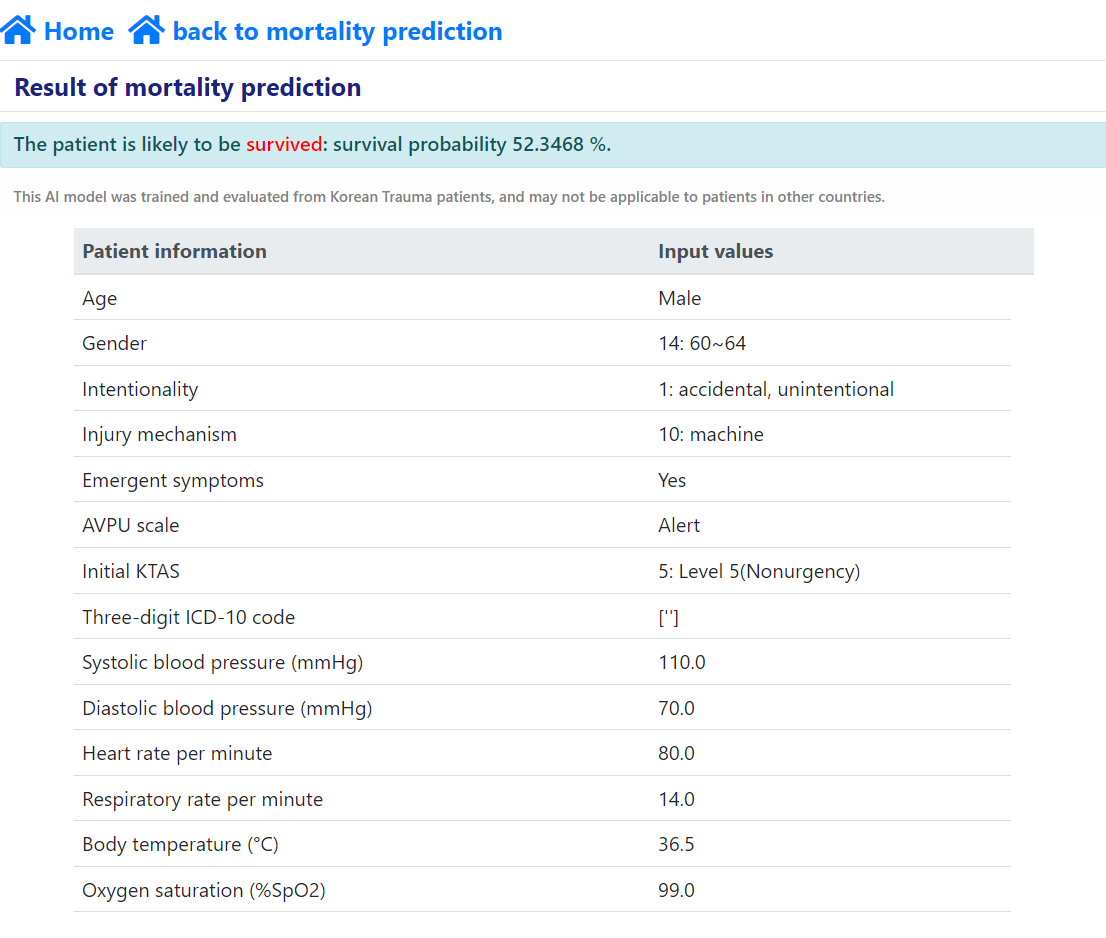

(B)

**Figure S1.** Deployed web application to provide mortality prediction in trauma patients: (A) User's web interface to enter information. (B) Prediction results with the probability of mortality.
